# Transcriptome Sequencing and Comparison of Venom Glands Revealed Intraspecific Differentiation and Expression Characteristics of Toxin and Defensin Genes in *Mesobuthus martensii* Populations

**DOI:** 10.3390/toxins14090630

**Published:** 2022-09-11

**Authors:** Zhiyong Di, Sha Qiao, Xiaoshuang Liu, Shuqing Xiao, Cheng Lei, Yonghao Li, Shaobin Li, Feng Zhang

**Affiliations:** 1 Key Laboratory of Zoological Systematics and Application of Hebei Province, College of Life Sciences, Hebei University, Baoding 071002, China; 2 Institute of Life Science and Green Development, Hebei University, Baoding 071002, China; 3 College of Life Sciences, Yangtze University, Jingzhou 434025, China

**Keywords:** intraspecific differentiation, *Mesobuthus martensii*, sexual dimorphism, toxin, transcriptome

## Abstract

*Mesobuthus martensii*, a famous and important Traditional Chinese Medicine has a long medical history and unique functions. It is the first scorpion species whose whole genome was sequenced worldwide. In addition, it is the most widespread and infamous poisonous animal in northern China with complex habitats. It possesses several kinds of toxins that can regulate different ion channels and serve as crucial natural drug resources. Extensive and in-depth studies have been performed on the structures and functions of toxins of *M*. *martensii*. In this research, we compared the morphology of *M. martensii* populations from different localities and calculated the COI genetic distance to determine intraspecific variations. Transcriptome sequencing by RNA-sequencing of the venom glands of *M*. *martensii* from ten localities and *M. eupeus* from one locality was analyzed. The results revealed intraspecific variation in the expression of sodium channel toxin genes, potassium channel toxin genes, calcium channel toxin genes, chloride channel toxin genes, and defensin genes that could be related to the habitats in which these populations are distributed, except the genetic relationships. However, it is not the same in different toxin families. *M*. *martensii* and *M. eupeus* exhibit sexual dimorphism under the expression of toxin genes, which also vary in different toxin families. The following order was recorded in the difference of expression of sodium channel toxin genes: interspecific difference; differences among different populations of the same species; differences between sexes in the same population, whereas the order in the difference of expression of potassium channel toxin genes was interspecific difference; differences between both sexes of same populations; differences among the same sex in different populations of the same species. In addition, there existed fewer expressed genes of calcium channel toxins, chloride channel toxins, and defensins (no more than four members in each family), and their expression differences were not distinct. Interestingly, the expression of two calcium channel toxin genes showed a preference for males and certain populations. We found a difference in the expression of sodium channel toxin genes, potassium channel toxin genes, and chloride channel toxin genes between *M*. *martensii* and *M. eupeus*. In most cases, the expression of one member of the toxin gene clusters distributed in series on the genome were close in different populations and genders, and the members of most clusters expressed in same population and gender tended to be the different. Twenty-one toxin genes were found with the MS/MS identification evidence of *M*. *martensii* venom. Since scorpions were not subjected to electrical stimulation or other special treatments before conducting the transcriptome extraction experiment, the results suggested the presence of intraspecific variation and sexual dimorphism of toxin components which revealed the expression characteristics of toxin and defensin genes in *M*. *martensii*. We believe this study will promote further in-depth research and use of scorpions and their toxin resources, which in turn will be helpful in standardizing the identification and medical applications of Quanxie in traditional Chinese medicine.

## 1. Introduction

*Mesobuthus martensii* is the most famous member of the family Buthidae (Arachnida: Scorpiones) in Asia. It is known for the widest distribution, the most complex habitats, and the largest biomass among scorpion species from China, as well as its extensive applications in traditional Chinese medicine. The species is distributed to latitude south of 43° N and the north sides of the Yangtze River, with its distribution bordered by the Helan Mountain and the Tengger Desert and Mo Us Desert in the west and north and limited by the sea in the east [1]. In the Chinese medicinal materials market, *M*. *martensii* is the primary component as one of the most famous animal-derived Chinese herbs, named “Quanxie”, which is the same as the stipulation of “Chinese Pharmacopoeia”.

Traditional Chinese medicine has played an important role during the outbreak of COVID-19. Quanxie was reported to have antiviral properties. “Xiang-su-hua-zhu Particle” created by Diangui Li, a great master of traditional Chinese medicine, was approved by the Hebei Drug Administration. Similarly, “Niu-huang-qing-nao-kai-qiao-wan” launched by Sihuan Aokang Pharmaceutical Co., Ltd. added Quanxie as a prescription for COVID-19. During the outbreak of SARS in 2003, the Nanshan Zhong medical team added Quanxie to both Kangyan II recipe and Kangyan III recipe (traditional Chinese medicine), with the following results: the days of hospitalization for 71 patients with SARS were 9 to 78 days (mean 27.1 ± 14.4 days). Seventy patients were healed (the healing rate was 98.6%), and one patient died; the mortality rate was 1.4% [2]. Some prescriptions of traditional Chinese medicine for treating plague, such as “Hui-sheng-zhi-bao-dan” in the “Xian-Nian-Ji” published in the Qing Dynasty, “Ji-jiu-wan” and “Bao-ying-duo-ming-dan” in the “National Formulary of Traditional Chinese Medicine”, contained Quanxie [3]. Since 1954, Keming Guo, a famous doctor of traditional Chinese medicine, used Quanxie and heat clearing and detoxifying herbs to save 34 patients with severe epidemic encephalitis type B patients. Quanxie has often been used to treat several viral diseases, with curative effects on children’s pneumonia and mumps, and inhibitory effects on influenza A virus H1N1, hand-foot-mouth disease of EV71 virus, and other viruses [4,5,6,7,8]. During the clinical application of Traditional Chinese Medicine in the treatment of viral infections, it is impossible to accurately judge the medicinal value of Quanxie as double-blind trials were not conducted; however, there is no doubt that it has long been valued by traditional Chinese medicine in such diseases.

Previous research suggested that the morphology and venom of *M*. *martensii* show intraspecific variations. Qi et al. (2004) reported differences in the body colors of the populations of *M*. *martensii* obtained from different localities [9]. For example, specimens collected from the Longhua County in Hebei Province were dark brown tergites, whereas those obtained from Baligou in Henan Province had dark red-brown tergites [9]. In other localities, *M*. *martensii* could have yellowish or pale yellow tergites [9]. Zhang & Zhu (2009) analyzed the intraspecific morphological differences of *M*. *martensii* from different localities in China; however, these differences were under subspecies level [10]. The results based on a few specimens with no gender information provided by Zhang & Zhu (2009) [10]. In addition, the LD50 values of *M*. *martensii* venom from different localities in mice were different: compared with the venom of populations obtained from Henan, Liaoning and Shandong Provinces, the LD50 value of venom of the population procured from Shanxi Province in mice was the smallest [11]. Shi et al. (2013) constructed a Bayesian evolutionary tree for species of *Mesobuthus* in China using COI and three nuclear genes, including 28 populations of *M*. *martensii*. The study revealed that *M*. *martensii* formed four branches in China: east China subclade, central north China subclade, isolated east China subclade, and widespread subclade [12]. In addition, Wang et al. (2019) analyzed the relationship between metabolic rate and the environment in 21 localities of *M*. *martensii* in China and revealed significant differences in the resting metabolic rate between sexes from the same locality and among different populations [13]. Similarly, Gao et al. (2021) reported differences in the expression patterns of toxin genes in different populations from four localities (Hebei, Henan, Shandong, and Shanxi Provinces) and genders (from Gansu Province) of *M*. *martensii* [14,15]. There exist certain cases of intraspecific variations of other species belonging to the genus *Mesobuthus*. For example, *M. eupeus*, which is a closely related species to *M*. *martensii*, has been divided into 23 subspecies [16].

A study of intraspecific variations can reveal the diversity of scorpion toxins. For instance, Abdel-Rahman et al. (2009) used polyacrylamide gel electrophoresis (SDS-PAGE) to analyze the venom of *Scorpio maurus palmatus* collected from four geographically isolated localities in Egypt [17]. The results showed differences in the expression of toxins obtained from different localities. Zhao et al. (2010) comprehensively analyzed venom transcriptomes (cDNA library) of the scorpion *Lychas mucronatus* from Hainan and Yunnan Provinces [18], revealing that the venom peptides and proteins of the same scorpion species from different geographical regions are highly diverse. Furthermore, scorpions evolved to adapt to new environments by altering the primary structure and abundance of their venom peptides and proteins. Carcamo-Noriega et al. (2018) investigated the venom collected from two distinct populations of the scorpion *Centruroides sculpturatus* that inhabit different regions of Arizona. The study reported intraspecific variations between venoms mostly in the composition and proportion of the two toxins (CsEv1 and CsEv2) [19].

The study on intraspecific variations in the transcriptome of *M*. *martensii* has a genomics foundation and a proteomics foundation. Cao et al. (2013) published the whole genome draft of *M*. *martensii* [20], which is the first sequenced whole genome of the order Scorpiones worldwide. Furthermore, the authors predicted the existence of at least 32,016 protein-coding genes, including 116 neurotoxin genes: 61 NaTx (toxins for sodium channels), 46 KTx (toxins for potassium channels), 5 ClTx (toxins for chloride channels), and 4 CaTx (toxins for ryanodine receptors) genes. Among these, 51 sodium channel toxin genes and potassium channel toxin genes were found to be clustered on 17 scaffold genes. In addition, six defensin genes were discovered by Cao et al. (2013) [20]. Subsequently, 153 fractions were isolated from the *M*. *martensii* venom by 2-DE, SDS-PAGE, and RP-HPLC, whereas 227 non-redundant protein sequences were unambiguously identified, consisting of 134 previously known and 93 unknown proteins [21]. Among 134 previously known proteins, 115 proteins were first confirmed from the *M*. *martensii* crude venom, and 19 toxins were confirmed once again, including 43 typical toxins, 7 atypical toxins, 12 venom enzymes, and 72 cell-associated proteins [21]. Li et al. (2016) comprehensively reviewed the diversity of toxin types, structures, and functions of *M*. *martensii* followed by a study on the genome and proteome of this species, and potential importance of scorpion toxins in medicine, including chronic pain relief, antitumor properties, anti-infection characteristics, and treatment of autoimmune diseases [22,23,24,25].

## 2. Results

### 2.1. Morphological Intraspecific Differentiation of Mesobuthus martensii

#### 2.1.1. Color

**Figure 1:**
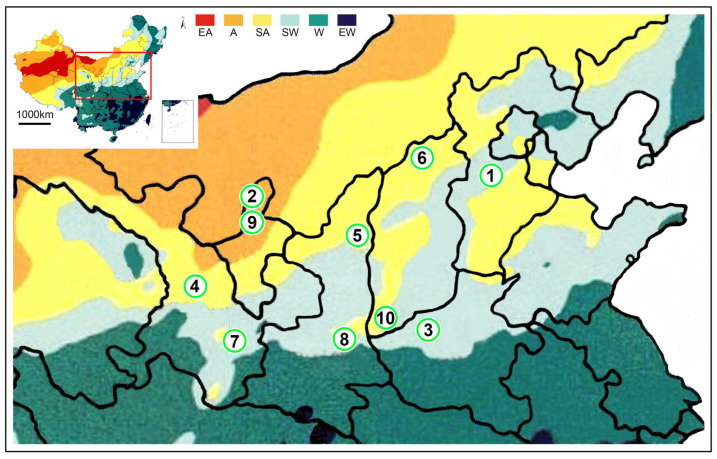
The distribution of populations of *Mesobuthus martensii* and *M. eupeus* from sub-wet areas, sub-arid areas, and arid areas in this study. *M*. *martensii*: 1, BD, Baoding; 2, HL, Helan Mountain (Helan); LY, Luoyang (3, LYF, females from Luoyang; same as 3′, LYM, males from Luoyang); LZ, Lanzhou (4, LZF, females from Lanzhou); 5, SD, Suide; SZ, Shuozhou (6, SZF, females from Shuozhou; same as 6′-SZM, males from Shuozhou); 7, TS, Tianshui; WN, Weinan (8, WNF, females from Weinan; same as 8′-WNM, males from Weinan); WZ, Wuzhong (9, WZF, females from Wuzhong); 10, YC, Yuncheng. *M. eupeus*: ME, from Yinchuan (11, MEF, females of from Yinchuan; same as 11′, MEM, males from Yinchuan, overlapping with the locality of 2 (HL, Helan)). EA, Extreme arid; A, Arid; SA, Sub-arid; SW, Sub-wet; W, Wet; EW, Extreme wet.

**Figure 2:**
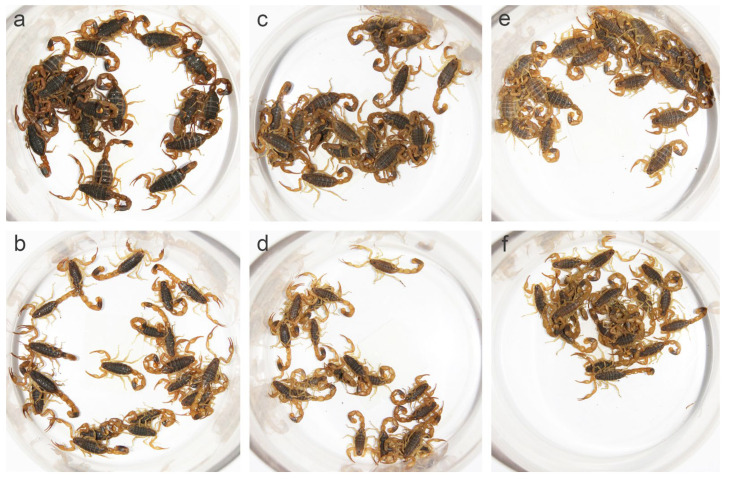
Color differences in *Mesobuthus martensii* populations from Luoyang (sub-wet area), Suide (sub-arid area), and Helan (arid area). (**a**,**b**) Females and males from Luoyang; (**c**,**d**) females and males from Suide; and (**e**,**f**) females and males from Helan.

As the color of specimens changed gradually after soaking in alcohol, we observed the live scorpions and fresh specimens obtained from different localities (Figure S1). Populations from arid areas were lighter than those from sub-arid and sub-wet areas. These differences were particularly significant in the carapace, tergites, and segments of metasoma. Most individuals from Luoyang and Baoding had black or black-brown carapace and tergites, whereas individuals from Lanzhou and Helan were yellow-brown in the same parts, which was identical in both sexes, but especially remarkable in females (Figure 1, Figure 2 and Figure S1). In addition, specimens from Luoyang and Baoding had yellow metasoma, yellowish brown carina, and yellowish appendages, whereas specimens from Lanzhou and Helan were lighter (Figure 2). This is consistent with the finding reported by Qi et al. (2004) [9]. Under the background of different annual precipitation and annual temperature in the eastern and western regions, different habitats were formed in these locations, thus promoting different physiological traits linked with evolutionary fitness among populations.

#### 2.1.2. Body Size

**Figure 3:**
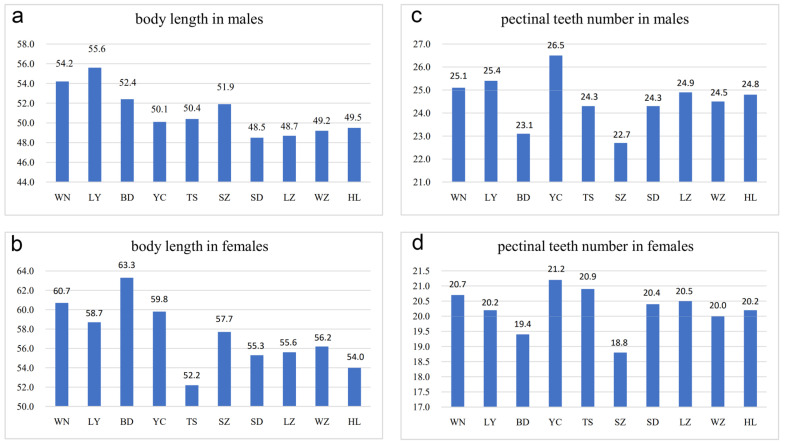
Differences in body length and pectinal teeth number among *Mesobuthus martensii* populations from sub-wet (WN, LY, BD, YC, TS), sub-arid (SZ, SD, LZ), and arid (WZ, HL) areas in China (Tables S1 and S2, means ± SD, *p* < 0.001 using a Kruskal–Wallis H test). (**a**) Body length in males; (**b**) body length in females; (**c**) pectinal teeth number in males; and (**d**) pectinal teeth number in females. BD, Baoding; HL, Helan; LY, Luoyang; LZ, Lanzhou; SD, Suide; SZ, Shuozhou; TS, Tianshui; WN, Weinan; WZ, Wuzhong; YC, Yuncheng.

Adult individuals from arid and sub-arid areas were smaller than those from sub-wet areas (Figure 3a,b). For example, the average body length of females from Baoding and Weinan was 63.3 mm (10 adults) and 60.7 mm (10 adults), respectively, whereas the average body length of females from Helan and Wuzhong was 54.0 mm (10 adults) and 56.2 mm (10 adults), respectively. The average body length of specimens from Helan, Lanzhou, Suide, and Wuzhong was similar but smaller than populations from wetter or warmer areas. As an exception, females from Tianshui, although living in the sub-wet area, had the smallest average size (average was 52.2 mm in 10 adults) which could be related to lower temperatures and lower humidity as well as a more barren habitat in Tianshui as compared to the other four populations from sub-wet areas (Baoding, Luoyang, Weinan and Yuncheng) (Figure 3a,b).

#### 2.1.3. Pectinal Teeth

Pectinal teeth constitute an important taxonomic feature of the order Scorpiones, especially in the genus *Mesobuthus*. Sun & Sun (2011) reported the difference in the number of pectinal teeth between the two subspecies, *M. caucasicus intermedius* (20–25 in females and 26–30 in males) and *M. caucasicus przewalskii* (15–19 in females and 19–23 in males) [26]. Qi et al. (2004) recorded the pectinal teeth count in *M. martensii*: 21–26 in males and 17–22 in females [9], which could be the common range in populations from Hebei Province, Henan Province, Inner Mongolia Autonomous Region, Liaoning Province, and Shanxi Province.

**Figure 4:**
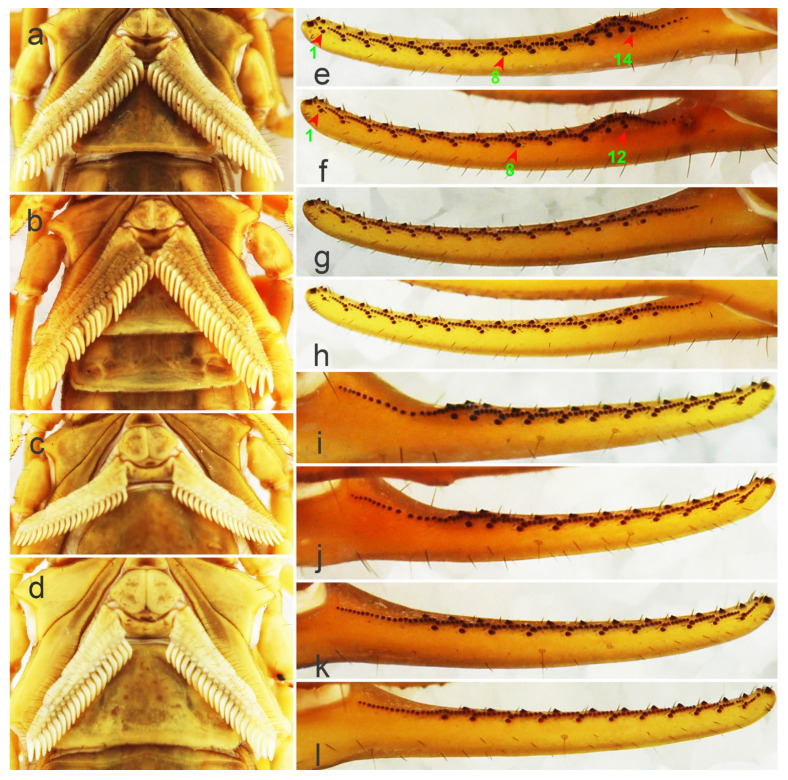
The pectines, moveable fingers, and fixed fingers of *Mesobuthus martensii* from Weinan and Helan. (**a**) Pectines of a male from Weinan; (**b**) pectines of a male from Helan; (**c**) pectines of a female from Weinan; (**d**) pectines of a female from Helan; (**e**) moveable finger of a male from Weinan showing 14 large granules, the numbers “1, 8, 14” are the serial numbers of large granules; (**f**) moveable finger of a male from Helan showing 12 large granules, the numbers “1, 8, 12” are the serial numbers of large granules; (**g**) moveable finger of a female from Weinan showing large granules; (**h**) moveable finger of a female from Helan showing large granules; (**i**) fixed finger of a male from Weinan showing large granules; (**j**) fixed finger of a male from Helan showing large granules; (**k**) fixed finger of a female from Weinan showing large granules; (**l**) fixed finger of a female from Helan showing large granules.

In this study, the number of pectinal teeth (PTN) was highly diverse among different populations (Figure 3c,d and Figure 4a–d). In males from Helan, PTN was 21–29 (average was 24.8 in 36 pectines), whereas in females, PTN was 18–22 (average was 20.2 in 42 pectines). In males from Wuzhong, PTN was 21–27 (average was 24.5 in 22 pectines) and 18–22 (average was 20.0 in 54 pectines) in females. In males from Yuncheng, PTN was 23–29 (average was 26.5 in 34 pectines) and 19–23 (average was 21.2 in 57 pectines) in females. In males from Weinan, PTN was 22–28 (average was 25.1 in 66 pectines) and 19–22 (average was 20.7 in 46 pectines) in females. Statistically, it indicates that the number of pectinal teeth in arid areas was slightly less than those in sub-wet areas, whereas Baoding is an exception, implying the intraspecific variation in pectines in *M. martensii* (Figure 3c,d and Figure 4a–d).

#### 2.1.4. Large Granules of Fingers

**Figure 5:**
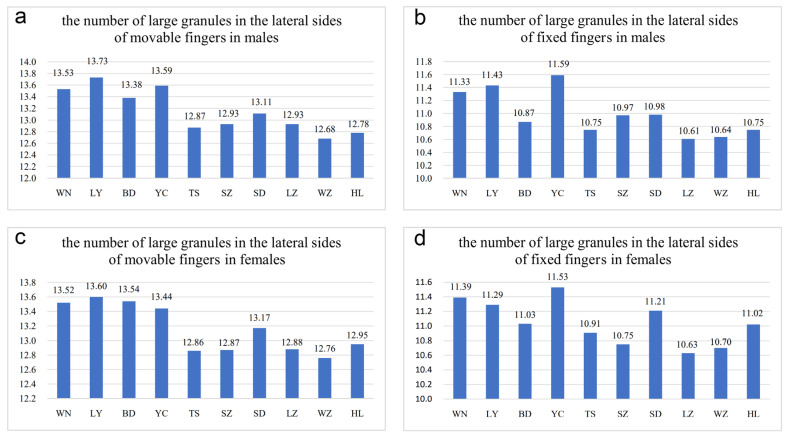
Differences in the number of large granules in the lateral sides of movable and fixed fingers among different *Mesobuthus martensii* populations from sub-wet (WN, LY, BD, YC, TS), sub-arid (SZ, SD, LZ), and arid (WZ, HL) areas in China (Tables S1 and S2: means ± SD, *p* < 0.001 using a Kruskal-Wallis H test). (**a**) The number of large granules in the lateral sides of movable fingers in males; (**b**) number of large granules in the lateral sides of fixed fingers in males; (**c**) the number of large granules in the lateral sides of movable fingers in females; (**d**) number of large granules in the lateral sides of fixed fingers in females. BD, Baoding; HL, Helan; LY, Luoyang; LZ, Lanzhou; SD, Suide; SZ, Shuozhou; TS, Tianshui; WN, Weinan; WZ, Wuzhong; YC, Yuncheng.

The number of oblique rows of movable finger teeth is another important identification characteristic of *Mesobuthus*. Qi et al. (2004) described movable and fixed fingers in both males and females of *M. martensii* composed of 12 oblique rows of granules [9]. Sun & Sun (2011) reported this characteristic in *M. caucasicus intermedius* (dentate margins of movable and fixed fingers with 12 and 11 oblique rows of granules, respectively) and *M. caucasicus przewalskii* (dentate margins of movable and fixed fingers with 11 and 10 oblique rows of granules, respectively) [26]. We found that slightly different teeth row shapes of *M. martensii* affected the calculation of number of teeth row by researchers. For example, individuals from Weinan and Helan (Figure 4e–l) showed differences in the shape of their oblique teeth rows that affected accurate recording of the number of teeth rows. Instead, we recorded the number of large granules in the lateral sides of movable fingers and fixed fingers (GMN & GFN, Figure 5).

In this study, the number of GMN and GFN in males was 12–14 (average was 12.78 in 36 specimens) and 10–12 (average was 10.75 in 36 specimens) in individuals from Helan, whereas this number was 12–14 (average was 12.95 in 42 specimens) and 10–12 (average was 11.02 in 41 specimens) in females, respectively. In the individuals from Wuzhong, the number of GMN and GFN in males was 12–14 (average was 12.68 in 22 specimens) and 10–11 (average was 10.64 in 22 specimens), but 11–14 (average was 12.76 in 54 specimens) and 10–12 (average was 10.70 in 54 specimens) in females. In individuals from Yuncheng, the number of GMN and GFN in males was 12–15 (average was 13.59 in 34 specimens) and 10–13 (average was 11.59 in 34 specimens), 12–14 (average was 13.44 in 55 specimens) and 10–12 (average was 11.53 in 57 specimens) in females. In individuals from Luoyang, the number of GMN and GFN in males was 11–15 (average was 13.73 in 44 specimens) and 11–12 (average was 11.43 in 44 specimens), 12–14 (average was 13.60 in 48 specimens) and 10–12 (average was 11.29 in 48 specimens) in females. This indicated that the number of large granules in the lateral sides of movable and fixed fingers in arid areas was slightly less than that in sub-wet areas and was similar to pectinal teeth Tianshui is an exception. The difference was significant, and it implies intraspecific variation in *M. martensii*.

### 2.2. Genetic Distance Revealed That All Populations from the Reported Distribution Range Belong to Mesobuthus martensii

Mirshamsi et al. (2010) reported that the intraspecific genetic distance of *M*. *eupeus*, the geographical closest relative of *M. martensii*, ranged from 4% to 7% [27]. We analyzed the genetic distance of COI sequences of *M. martensii* from 44 localities and the result was 0.2% to 6.2% (Supplementary Information S1). These 44 localities covered the representative localities of the distribution area of *M. martensii*, supporting that the 10 populations of wild *M. martensii* in this research belonged to the same species.

### 2.3. Comparison of Venom Gland Transcriptomes Revealed Intraspecific Variations in the Expression of Toxin Genes in Mesobuthus martensii

To reveal the difference in venoms collected from different populations of *M*. *martensii* from different localities, we sequenced the transcriptomes of mixed venom glands of both sexes from five wild populations (Baoding, Yuncheng, Tianshui, Suide, and Helan). Supplementary Information S2 includes the FPKM of toxin genes of different populations of *M*. *martensii* and *M*. *eupeus*.

#### 2.3.1. Expression of Sodium Channel Toxin Genes in Different Populations

**Figure 6:**
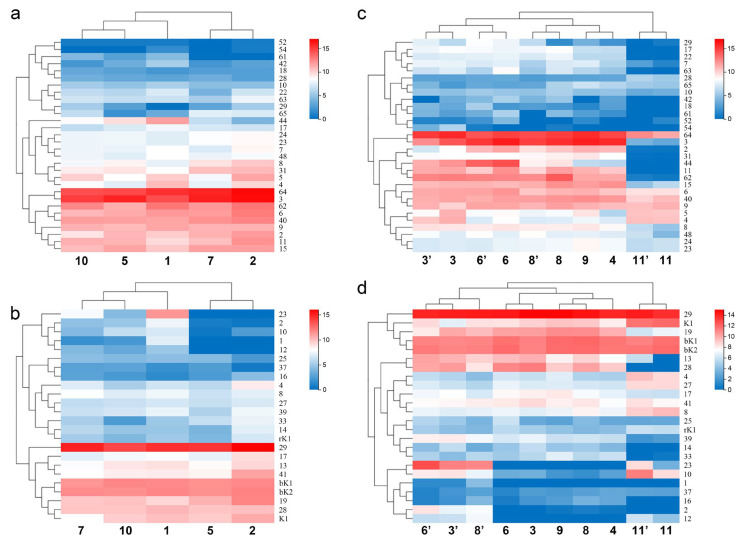
Population and gender differences in the expression of sodium channel toxin genes and potassium channel toxin genes of *Mesobuthus martensii* in China. (**a**,**b**) Heat map showing the difference in the gene expression of sodium channel toxin genes and potassium channel toxin genes among the populations of *M*. *martensii*; (**c**,**d**) heat map showing the difference in the gene expression of sodium channel toxin genes and potassium channel toxin genes between sexes of *M*. *martensii* and *M. eupeus*. Populations of *M*. *martensii* present in X axis: 1, BD, Baoding; 2, HL, Helan; LY, Luoyang (3, LYF, females from Luoyang; 3′, LYM, males from Luoyang); LZ, Lanzhou (4, LZF, females from Lanzhou); 5, SD, Suide; SZ, Shuozhou (6, SZF, females from Shuozhou; 6′, SZM, the males from Shuozhou); 7, TS, Tianshui; WN, Weinan (8, WNF, females from Weinan; 8′, WNM, males from Weinan); WZ, Wuzhong (9, WZF, females from Wuzhong); 10, YC, Yuncheng. The population of *M. eupeus* present in X axis: ME, Yinchuan (11, YCF, females of from Yinchuan; 11′, YCM, the males from Yinchuan). Numbers on Y axis represent gene names, (**a**,**b**) BmKNaTx; (**c**,**d**) BmKaKTx, bK-BmKbKTx, rK-BmKrKTx, K-BmKKTx. Please see Supplementary Information S2 for more details. The expression level was presented as log2(FPKM+1).

Sodium channel scorpion toxins exist widely in the order Scorpiones. It belongs to the long-chain scorpion neurotoxin, which contains 58 to 76 amino acid residues [22,23]. It is a molecular weapon that plays a major role in scorpion venom. When a scorpion injects its venom into its prey, sodium channel toxins make its prey produce spasms or undergo paralysis. The difference in the expression of 30 sodium channel toxin genes (including four putative new members: MMa12627 (BmKNaTx62), MMa29116 (BmKNaTx63), MMa34629 (BmKNaTx64) and MMa38588 (BmKNaTx65)) from different populations showed intraspecific variations (Figure 6a).

The expression (FPKM value) of MMa23370 (BmKNaTx44) in Helan and Tianshui was 16.08 and 89, respectively, whereas the expression in Yuncheng, Suide and Baoding was 320.37, 792.47, and 3300.75, respectively. The difference in the expression between Baoding and Helan differed by 205 times. The expression between Helan and Tianshui was close, and that between Yuncheng and Suide was close. The expression of MMa38588 (BmKNaTx65) in Helan and Tianshui was 153.82 and 188.08 and Suide was 2.89. The difference between the maximum and the minimum expression was 65 times. The expression of MMa53032 (BmKNaTx29) in Baoding, Suide, and Tianshui was 0.14, 6.26, and 7.69, respectively, and Helan and Yuncheng was 42.87 and 44.66. The expression of MMa20191 (BmKNaTx42) in Tianshui, Yuncheng, and Helan was 3.62, 3.9, and 7.06, respectively; whereas in Suide and Baoding, the expression was 11.58 and 46.72 respectively. The difference between the maximum and the minimum expression was 13 times. In addition to the intraspecific differentiation in the expression of toxin genes, the heat map of gene expression of sodium channel toxins showed that the closer the localities of populations, the higher the similarity: Helan and Tianshui were present in one subclade, while Baoding, Yuncheng and Suide were together (Figure 6a). This may suggest a link to the relatedness of populations in *M. martensii*.

#### 2.3.2. Expression of Potassium Channel Toxin Genes in Different Populations

Potassium channel scorpion toxins act on the potassium channel protein of the cell membrane. They act on different types of potassium channels and exert various biological functions [22,23]. The difference in the expression of 24 potassium channel toxin genes from different populations showed intraspecific variations too (Figure 6b).

The expression of MMa16285 (BmKaKTx1) in Helan and Suide was 0, in Tianshui it was 2.48, 3.72 in Yuncheng, and in Baoding it was 133.1. The expression of MMa16284 (BmKaKTx2) in Helan and Suide was 0.33 and 0.66 respectively, in Tianshui it was 22.11, in Yuncheng it was 27.83, and in Baoding it was 175.59. The expression of MMa35044 (BmKaKTx10) in Helan and Suide was 4.42 and 0.37, respectively. In Tianshui it was 16.67, in Yuncheng it was 66.96, and in Baoding it was 109.7. The expression of MMa35043 (BmKaKTx12) in Helan and Suide was 0; in Yuncheng it was 7.66; in Tianshui it was 15.89; and in Baoding it was 27.89. The expression of MMa05343 (BmKaKTx23) in Helan and Suide was 0, in Yuncheng was 16.99, in Tianshui was 218.82, and in Baoding was 2490.09. The expression of MMa34788 (BmKaKTx33) in Yuncheng was 4.28, in Baoding was 29.87, in Tianshui was 37.86, in Suide was 71.39, and in Helan was 169. In addition to the intraspecific differences in the expression of toxin genes, similar to sodium channel toxin genes, the heat map of gene expression of potassium channel toxins showed that the closer the localities of populations, the higher the similarity. The difference in the gene expression of sodium channel toxin genes was sorted by regions: Helan and Suide, one arid and one sub-arid area with average annual temperature below 10 °C, present in one subclade, and Baoding, Tianshui and Yuncheng, three sub-wet areas with average annual temperature above 10 °C in another subclade (Figure 6b). Interestingly, it also seems to be related to the humidity and temperature of the localities (Figure 1).

#### 2.3.3. Expression of Calcium Channel Toxin Genes, Chloride Channel Toxin Genes, and Defensin Genes in Different Populations

**Figure 7:**
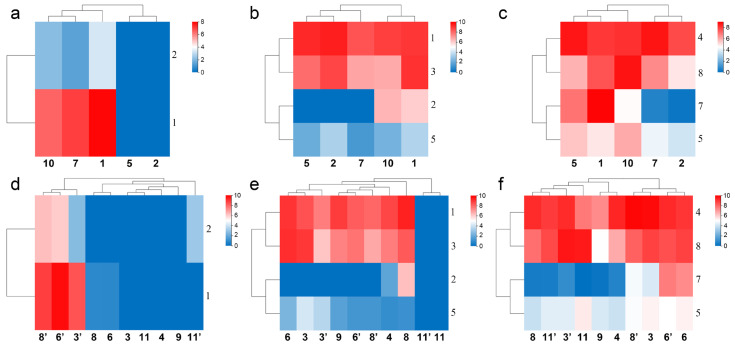
Populations and gender differences in the gene expression of calcium channel toxin genes, chloride channel toxin genes, and defensin genes of *M. martensii* in China. (**a**–**c**) Heat maps of expression differences of calcium channel toxin genes, chloride channel toxin genes, and defensin genes in different populations of *M*. *martensii*; (**d**–**f**) heat maps of expression differences of calcium channel toxin genes, chloride channel toxin genes, and defensin genes in different sexes of *M*. *martensii* and *M. eupeus*. Populations of *M*. *martensii* present in X axis: 1, BD, Baoding; 2, HL, Helan; LY, Luoyang (3, LYF, females from Luoyang; 3′, LYM, males from Luoyang); LZ, Lanzhou (4, LZF, females from Lanzhou); 5, SD, Suide; SZ, Shuozhou (6, SZF, females from Shuozhou; 6′, SZM, males from Shuozhou); 7, TS, Tianshui; WN, Weinan (8, WNF, females from Weinan; 8′, WNM, males from Weinan); WZ, Wuzhong (9, WZF, females from Wuzhong); 10, YC, Yuncheng. The population of *M. eupeus* present in X axis: ME, Yinchuan (11, YCF, females of from Yinchuan; 11′ YCM, males from Yinchuan). Numbers on Y axis represent gene names, (**a**,**d**) BmKCaTx; (**b**,**e**) BmKClTx; (**c**,**f**) BmKDfsin. Please see Supplementary Information S2 for more details. The expression level was presented as log2(FPKM+1).

The structure and function of calcium channel scorpion toxins and chloride channel scorpion toxins have not been widely studied, except for CTX [22,23]. There were just 2–4 expressed genes available for comparison in every gene family. Similar to sodium channel and potassium channel toxin genes, differences in their gene expression and defensin genes also showed intraspecific variations (Figure 7a–c). The expression of MMa44674 (BmKClTx2) in Helan, Suide, and Tianshui was 0, in Yuncheng was 86.45, and in Baoding was 61.93. The expression of MMa15573 (BmKCaTx1) in Helan and Suide was 0, in Yuncheng was 85.4, in Tianshui was 128.08, and in Baoding was 238.5. The expression of MMa48745 (BmKCaTx2) in Helan and Suide was 0, in Yuncheng was 3.42, in Tianshui was 1.68, and in Baoding was 9.12. Six defensin genes of *M*. *martensii* were reported [20]. Two new defensin genes MMa09285 (BmKDfsin7) and MMa39355 (BmKDfsin8) were putative (Supplementary Information S3). The expression of MMa09285 (BmKDfsin7) in Helan and Tianshui was 0.15 and 0.51, in Yuncheng was 22.73, in Suide was 124.08, and in Baoding was 489.35. Unlike the sodium channel and potassium channel toxin genes, the heat map of gene expression of these genes did not gather according to the distribution of populations conformably; however, it suggests a relationship between clustering and humidity: Helan and Suide or Tianshui gathered in one subclade (Figure 7a,c), and Baoding and Yuncheng, or Baoding, Tianshui, and Yuncheng gathered in another subclade (Figure 7a,b). It is important to note that fewer genes may not reflect true intraspecific differentiation in *M. martensii*.

### 2.4. Expression of Toxin Genes Indicated Sexual Dimorphism of Venom in Mesobuthus martensii

To reveal the difference in venoms of different sexes, we sequenced the transcriptomes of both sexes of populations from Luoyang, Shuozhou, and Weinan of *M*. *martensii* and *M. eupeus* from Yinchuan and the females of *M*. *martensii* from Wuzhong and Lanzhou. Supplementary Information S2 includes the FPKM of toxin genes of different sexes in the gene expression of *M*. *martensii* and *M*. *eupeus*.

#### 2.4.1. Expression Differences of Sodium Channel Toxin Genes in Different Populations Are More than That in Different Sexes from the Same Population

By clustering with the differences in the expression of toxin genes of both sexes of *M. eupeus*, it was found that the difference in the expression of sodium channel toxin genes showed the following trend: interspecific difference > differences among different populations of the same species > differences between the sexes of the same population (Figure 6c).

The expression of MMa17864 (BmKNaTx5) in males from Luoyang was 413.1, and in females from Luoyang it was 2134.35, a difference that was five times larger in females than males. The expression of MMa29117 (BmKNaTx11) in males from Shuozhou was 1184.93, and in females from Shuozhou it was 4641.17, a difference that was four times larger in females than in males. In males from Weinan it was 974.72, and in females from Weinan it was 3305.2, a difference that was three times larger in females than males. Therefore, the expression of these sodium channel toxin genes of females was higher than that in males. The expression of MMa35303 (BmKNaTx18) in females from Shuozhou was 3.89, and in males from Shuozhou it was 47.85, a difference that was 12 times larger in males than females. In females from Luoyang it was 8.97, and in males from Luoyang it was 17.6, a difference that was two times larger in males than females. This is an example for the expression of sodium channel toxin genes of males was higher than that of females. Some similar examples are as follows. The expression of MMa53032 (BmKNaTx29) in females from Weinan was 2.18, and in males from Weinan it was 81.24, a difference that was 37 times larger in males as compared to females. The expression of MMa20191 (BmKNaTx42) in males from Luoyang was 0, and in females from Luoyang it was 11.43. The expression of MMa38588 (BmKNaTx65) in males from Weinan was 8.8, and in females from Weinan it was 55.85, the difference was six times larger in females than in males. In females from Luoyang, the expression was 4.42, and in males from Luoyang it was 16.73, a difference that was four times larger in males than in females. The expression of MMa14634 (BmKNaTx61) in males from Weinan was 0, and in females from Weinan it was 20.93. For males from Shuozhou it was 12.04, and for females from Shuozhou it was 52.42; the difference was four times larger in females than in males. In females from Luoyang gene expression was 6.05, and in males from Luoyang it was 16.06; the difference was three times larger in males than in females. The expression of MMa29116 (BmKNaTx63) in males from Shuozhou was 58.95, and in females from Shuozhou was 394.8, the difference was seven times larger in females than in males. In males from Weinan gene expression was 35.93, and in females from Weinan it was 144.83; the difference was four times larger in females than males.

In addition to the intraspecific differentiation in the expression of toxin genes, the heat map of gene expression of sodium channel toxins in different sexes from the different populations and species also showed that the closer the localities of populations, the higher the similarity: Lanzhou (sub-arid area) and Wuzhong (arid area) were present in one subclade, while Luoyang and Shuozhou (two sub-wet areas) were together (Figure 6c). It is important to note that although Weinan (sub-wet area) is closer to Shuozhou, the gene expression of its population is clustered with that of Wuzhong and Lanzhou. This may suggest a link to the relatedness of populations in *M. martensii*.

#### 2.4.2. Expression Differences of Potassium Channel Toxin Genes in the Same Sexes from Different Populations of the Same Species Are Less than That in Different Sexes from the Same Population

The expression of potassium channel toxin genes also showed intraspecific sexual dimorphism. Clustering with the expression difference of potassium channel toxin genes of *M. eupeus*: interspecific difference > differences between both sexes of same populations > same-sex of different populations of same species (Figure 6d).

The expression of MMa16285 (BmKaKTx1) in females from Weinan was 0, and in males it was 15.44. There was no expression of MMa16285 (BmKaKTx1) in males and females of *M. eupeus*. The expression of MMa16284 (BmKaKTx2) in females from Luoyang was 0, in males was 112.96; in females from Weinan was 0.66, in males was 158.65; in females from Shuozhou was 0, in males was 316.69; in females, the expression was very low or no expression; in males, the expression was high. There was no expression of MMa16284 (BmKaKTx2) in males and females of *M. eupeus*. The expression of MMa35044 (BmKaKTx10) in females from Luoyang was 9.18, and in males it was 321.87, the difference was 35 times; that in females from Shuozhou was 8, and in males was 271.67, the difference was 34 times; in females from Weinan it was 2.09, in males was 120.26, the difference was 58 times; in females, the expression was low; in males, the expression was high. In *M. eupeus* females, the expression of MMa35044 (BmKaKTx10) was 370.92; in males, it was 2481.21. The expression of MMa35043 (BmKaKTx12) in females from Luoyang was 0, in males was 57.88; that in females from Shuozhou was 0, in males was 52.03; in females from Weinan was 0, in males was 137.46; the potassium channel toxin gene of female *M. martensii* was not expressed; in males, it was highly expressed. In *M. eupeus* females, the expression was 18.76; in males, it was 61.07. The expression of MMa05343 (BmKaKTx23) in females from Luoyang was 0; in males, it was 2424.3; in females from Shuozhou, it was 0; in males, it was 6707.88; in females, from Weinan it was 0; in males, it was 2095.2; the potassium channel toxin gene of female *M. martensii* was not expressed; but in males, it was highly expressed. In *M. eupeus* females, the expression of MMa05343 (BmKaKTx23) was 9.11 and in males, it was 368.02.

Although both sexes of one population gathered in different subclades, but same sex of Luoyang and Shuozhou (two close sub-wet areas) together respectively (Figure 6d). It may suggest a link to the relatedness or habitats of populations in *M. martensii*.

#### 2.4.3. Expression Differences of Calcium Channel Toxin Genes, Chloride Channel Toxin Genes, and Defensin Genes in Sexes from Different Populations

The expression of calcium channel toxin genes suggested a population difference (Figure 7a) and sexual dimorphism (Figure 7d). Clustering with the expression difference of calcium channel toxin genes of *M. eupeus* showed male preference, all females of different populations of *M*. *martensii* and both sexes of *M. eupeus* showed low expression (Figure 7d). Clustering with the expression difference of toxin genes of both sexes of *M. eupeus* showed a difference in the expression of chloride channel toxin genes in different populations (Figure 7b) and different sexes (Figure 7e) of *M*. *martensii*; however, no difference was observed in both sexes of *M. eupeus*: interspecific difference > intraspecific differences, whereas neither of the same sex of different populations or both sexes of the same population of *M*. *martensii* showed more similarity (Figure 7e). All four chloride channel toxin genes showed no expression in *M. eupeus*, suggesting significant interspecific differences with *M*. *martensii*, whereas the two chloride channel toxin genes showed low expression in the latter. There is no logical explanation for the heat map showing the differences in the expression of defensin genes, except that both sexes from Shuozhou gathered in one subclade (Figure 7f). The expression of MMa44674 (BmKClTx2) in males from Weinan was 0; in females, it was 75.28; in males and females from Luoyang and Shuozhou, it was 0. The expression of MMa00982 (BmKClTx3) in males from Luoyang was 69.34; in females, it was 483.25, the difference was seven times. The expression of MMa15573 (BmKCaTx1) in females from Luoyang was 0, whereas in males, it was 337.25; in females, it was 0.8 from Shuozhou, whereas in males, it was 880.28; in females from Weinan, it was 0.83, whereas in males, it was 434.7; in females of *M. martensii*, the expression was extremely low or no expression; in males, it was high. In *M. eupeus*, the females did not show expression. This indicated that calcium channel toxin genes were not expressed in females. The expression of MMa48745 (BmKCaTx2) in females from Luoyang was 0, whereas in males, was 5.25; in females from Shuozhou, it was 0, whereas in males, it was 63.44; in females from Weinan, it was 0, whereas in males, it was 76.54; in females of *M. martensii*, it was not expressed; in males, it was low or high. In *M. eupeus*, the females did not express it. This indicated that calcium channel toxin genes were not expressed in females. The expression of MMa09285 (BmKDfsin7) in males from Luoyang was 1.01; in females, it was 18.12; the difference was 18 times. The expression of MMa09285 (BmKDfsin7) in males from Weinan was 27.12; in females, the expression was 0.33.

### 2.5. Expression of Toxin Genes in the Same Cluster of Mesobuthus martensii Genome

**Figure 8:**
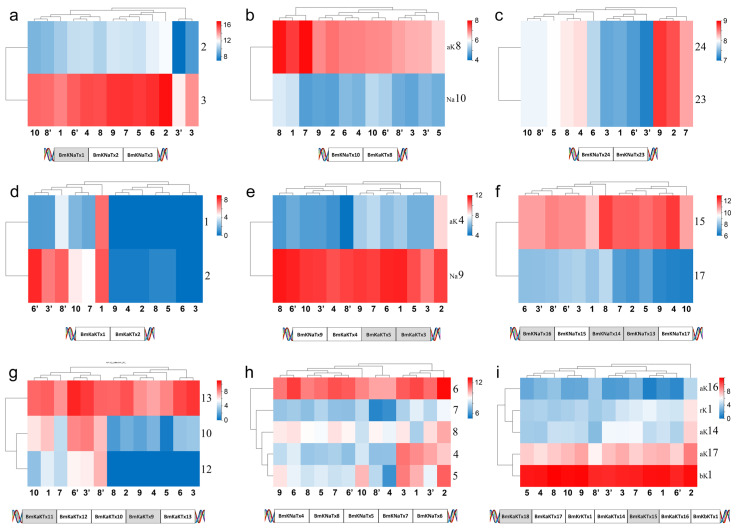
Expression of toxin gene cluster members in the *Mesobuthus martensii* genome, shows that the expression of one gene in the cluster was close in different populations and genders, and the members of most clusters expressed in same population and gender tended to be the different. The silent genes (with shadow in the schematic diagram of the sequence of genes on DNA) was the same in different populations and genders. The populations of *M*. *martensii* present in X axis: 1, BD, Baoding; 2, HL, Helan; LY, Luoyang (3, LYF, females from Luoyang; 3′, LYM, males from Luoyang); LZ, Lanzhou (4, LZF, females from Lanzhou); 5, SD, Suide; SZ, Shuozhou (6, SZF, females from Shuozhou; 6′, SZM, males from Shuozhou); 7, TS, Tianshui; WN, Weinan (8, WNF, females from Weinan; 8′, WNM, males from Weinan); WZ, Wuzhong (9, WZF, females from Wuzhong); 10, YC, Yuncheng. Population of *M. eupeus* present in X axis: ME, Yinchuan (11, MEF, females from Yinchuan; 11′, MEM, males from Yinchuan). Numbers the Y axis represents gene names, (**a**,**c**,**f**,**h**) BmKNaTx; (**d**,**g**) BmKaKTx; (**b**,**e**,**i**) Na-BmKNaTx, aK-BmKaKTx, bK-BmKbKTx, and rK-BmKrKTx. Please see Supplementary Information S2 for more details. The expression level was presented as log2(FPKM+1).

Nine of 17 toxin gene clusters reported in the genome sequencing of *M. martensii* showed expression [20,28]. The expression of one gene in the cluster was close in different populations and genders whereas it is not same in different members in the same populations and genders (Figure 8a–c,e,f,i). In the case of selective expression in members of the same cluster, certain members did not express in the normal state but were expressed either as silent or low in different populations or genders (Figure 8a,d,g,h). In addition, there are several cases in which the members of certain gene clusters were not expressed, and it is unclear why they are or are not expressed. (1) BmKNaTx1 was absent; and BmKNaTx3 was relatively highly expressed, whereas BmKNaTx2 was lowly expressed in *M*. *martensii* (Figure 8a). (2) The expression of BmKaKTx1 and BmKaKTx2 in females was very low or even not present in *M*. *martensii*, except the population from Baoding; however, the expression was relatively high in males (Figure 8d). (3) The expression difference of BmKNaTx4, BmKNaTx8, BmKNaTx5, and BmKNaTx7, and BmKNaTx6 in *M*. *martensii* was large (Figure 8h). (4) The expression of BmKNaTx9 was high relatively, whereas the expression of BmKaKTx5 and BmKaKTx3 was absent and BmKaKTx4 was low (Figure 8e). (5) The expression of and BmKaKTx8 was relatively high, whereas the expression of BmKNaTx10 was low in *M*. *martensii* (Figure 8b). (6) The expression of BmKNaTx15 was high relatively and BmKNaTx17 was low, whereas BmKNaTx16, BmKNaTx14, and BmKNaTx13 were not expressed in *M*. *martensii*; the order of this cluster is BmKNaTx16, BmKNaTx15, BmKNaTx14, BmKNaTx13, and BmKNaTx17 (Figure 8f). (7) The expression of BmKaKTx12 and BmKaKTx10 in females was very low or no expression in *M*. *martensii*, whereas the expression was relatively high in males. BmKaKTx13 was highly expressed relatively in different populations and genders, whereas BmKaKTx11 and BmKaKTx9 were not expressed in *M*. *martensii*. The order of this cluster is BmKaKTx11, BmKaKTx12, BmKaKTx10, BmKaKTx9, and BmKaKTx13 (Figure 8g). (8) The expression of BmKaKTx17 was high relatively, that of BmKrKTx1, BmKaKTx14, and BmKaKTx16 was low, and that of BmKbKTx1 was high; whereas BmKaKTx18, BmKaKTx15 were not expressed. The order of this cluster was BmKaKTx18, BmKaKTx17, BmKrKTx1, BmKaKTx14, BmKaKTx15, BmKaKTx16, and BmKbKTx1 (Figure 8i). (9) The expression of BmKNaTx24 and BmKNaTx23 was the same in same population (Figure 8c).

### 2.6. The Protein Evidences of Expressed Toxin Genes in Mesobuthus martensii

The protein evidences of expressed toxin genes, the multiple sequence alignment of four putative new toxin genes named following Cao et al., (2013) [20] (MMa12627 (BmKNaTx62), MMa29116 (BmKNaTx63), MMa34629 (BmKNaTx64) and MMa38588 (BmKNaTx65)), and the peptide sequences from *M*. *martensii* venom samples were provided (Supplementary Information S3). Twenty-one expressed genes with the MS/MS identification evidences of the *M*. *martensii* venom samples separated by 2-DE, SDS-PAGE, and RP-HPLCa by Xu et al., (2014) [21]. Five genes with yellow shadow have the inconclusive evidences due to the inconsistency of an amino acid residue between the multiple alignment sequences of putative toxin genes and the peptide sequences from Xu et al. (2014) [21] (Supplementary Information S3).

### 2.7. Validation of RNA-Seq Data by RT-qPCR Analysis

Two expressed genes namely MMa05343 (BmKaKTx23) and MMa35043 (BmKaKTx12) with |log2((♂-FPKM+1)/(♀-FPKM+1))| ≥ 5 from Shuozhou and Weinan populations, and eight expressed genes, namely, MMa29117 (BmKNaTx11), MMa34788 (BmKaKTx33), MMa17863 (BmKNaTx8), MMa17863-1 (BmKNaTx8-1), MMa17863-2 (BmKNaTx8-2), MMa21265 (BmKaKTx4), MMa21265-1 (BmKaKTx4-1), and MMa21265-2 (BmKaKTx4-2) from Helan (HL) and Baoding (BD) populations with |log2((HL-FPKM+1) /(BD-FPKM+1))| ≥ 2 were selected. Among them, -1 and -2 indicate that the gene had multiple different transcripts. The transcriptome data were verified by RT-qPCR analysis.

**Figure 9:**
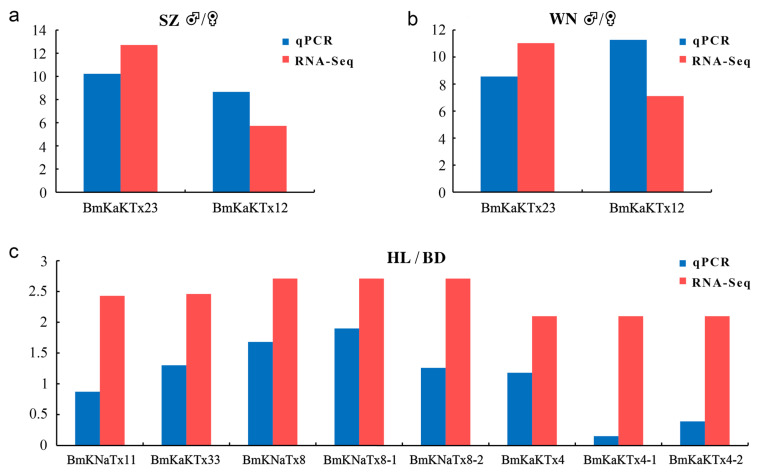
Comparison of gene expression data obtained by RT-qPCR and RNA-seq. (**a**) Differential expression multiple of RT-qPCR and RNA-seq in both sexes of Shuozhou population; (**b**) differential expression multiple of RT-qPCR and RNA-seq in both sexes of Weinan population; (**c**) differential expression multiple of RT-qPCR and RNA-seq in Baoding and Helan populations. BD, Baoding; HL, Helan; SZ, Shuozhou; WN, Weinan. Y asix represents the expression level of genes, obtained by RT-qPCR was presented as: |log2(♂-RE/♀-RE)|; obtained by RNA-seq was presented as: (**a**,**b**) value = |log2((♂-FPKM+1)/(♀-FPKM+1))|, (**c**) value = |log2((HL-FPKM+1)/(BD-FPKM+1))|. RE, relative expression level.

In this study, we compared the expression data obtained by RT-qPCR (blue column) and RNA-seq (red column). The ♂/♀ expression level ratio of gene MMa05343 (BmKaKTx23) from Shuozhou (SZ) by RT-qPCR was 10.22, and the value in FPKM by RNA-seq was 12.71 (Figure 9a). The ♂/♀ expression level ratio of gene MMa35043 (BmKaKTx12) from Shuozhou by RT-qPCR was 8.66, and the value in FPKM by RNA-seq was 5.73 (Figure 9a). The ♂/♀ expression level ratio of gene MMa05343 (BmKaKTx23) from Weinan (WN) ♂/♀ by RT-qPCR was 8.56, and the value in FPKM by RNA-seq was 11.03 (Figure 9b). The ♂/♀ expression level ratio of gene MMa35043 (BmKaKTx12) from Weinan by RT-qPCR was 11.27, and the value in FPKM by RNA-seq was 7.11 (Figure 9b). The Helan (HL)/Baoding (BD) expression level ratio of gene MMa29117 (BmKNaTx11) by RT-qPCR was 0.87, and the value in FPKM by RNA-seq was 2.43 (Figure 9c). The HL/BD expression level ratio of gene MMa34788 (BmKaKTx33) by RT-qPCR was 1.30, and the value in FPKM by RNA-seq was 2.46. The HL/BD expression level ratio of gene MMa17863 (BmKNaTx8) by RT-qPCR was 1.68, and the value in FPKM by RNA-seq was 2.71. The HL/BD expression level ratio of gene MMa17863-1 (BmKNaTx8-1) by RT-qPCR was 1.90, while the HL/BD expression level ratio of gene MMa17863-2 (BmKNaTx8-2) by RT-qPCR was 1.26 (Figure 9c). The HL/BD expression level ratio of gene MMa21265 (BmKaKTx4) by RT-qPCR was 1.18, and the value in FPKM by RNA-seq was 2.10. The HL/BD expression level ratio of gene MMa21265-1 (BmKaKTx4-1) by RT-qPCR was 0.15, while the HL/BD expression level ratio of gene MMa21265-2 (BmKaKTx4-2) by RT-qPCR was 0.39.

The analysis revealed that the expression trend of the selected expressed genes was basically the same as that of RNA-seq; however, the expression multiples were slightly different, indicating that the results of RNA-seq in this study had good accuracy and credibility.

## 3. Discussion

*M*. *martensii* is widely distributed in the vast and complex habitats of northern China. Intraspecific variation majorly contributes to their survival and adaptation. The results revealed differences in intraspecific variations in the morphology, color, toxin gene expression, and defensin gene expression in *M*. *martensii*. In response to the complex living environment, different populations are subjected to variable ecological factors and dual selection pressures from biological and abiotic factors.

*M. martensii* is an important component of traditional Chinese medicine, with a long medical history and unique functions. It has several kinds of toxins that can regulate manifold ion channels specifically and can serve as a crucial natural drug resources. In addition, its defensins play an important role in its survival and adaptation.

In recent years, intemperance in excessive collection and difficulty in artificial reproduction have led to severe destruction of natural resources and a decline in the population of *M. martensii*. Fake medicines containing other species belonging to the same genus and even different families or genera are considerably common in traditional Chinese medicinal materials. Closely related species of *M. martensii* from the same genus are widespread and similar to each other, whereas genuine medicinal materials of *M. martensii* have a large region of distribution. Intraspecific differentiation without accurate identification criteria has led to complicated medicinal material sources of Quanxie. It has severely affected accurate clinical prescription and medical research in the area of traditional Chinese medicine. Investigating its medicinal material resources and revealing its intraspecific differentiation, defensin gene and toxin gene resources will promote further in-depth research for the use of scorpions and their defensive toxins which in turn will be helpful in standardizing the identification and medical applications of Quanxie in traditional Chinese medicine.

Scorpions, as cold-blooded animals, rely on their own behavior to regulate body heat emission or absorb heat from the external environment to raise their body temperature. So the ambient temperature affects their metabolism. Therefore, it’s likely that their body size (directly proportional to body weight) is also influenced by ambient temperature. In addition, it is limited by the situation of prey (richness and size, etc.). The color of their body is similar to the surrounding environment which is more conducive to the “waiting” hunting method, whereas the surrounding environment is closely related to temperature and humidity. For pectinal teeth, we speculate that the habitat in high humidity areas are more complex, whereas it is desolate and simple in low humidity areas. The size and number of pectinal teeth are related to chemical sensors. Since the airflow in complex areas is less than that in the open and desolate areas, scorpion species require a better sense of smell. However, variations in the number of large granules in the lateral sides of moveable and fixed fingers are difficult to contact with external factors. It could only be related to the size of the body as the number of large granules in the lateral sides of the moveable and fixed fingers corresponds to the number of pectinal teeth and the size of the body.

Under normal conditions, we could identify only 60 toxin genes in different populations, accounting for only about 50% of the known toxin gene members in the genome. There exist certain differences among different populations. It is not known why only these toxin genes were expressed and what is the biological significances of the difference in the expression. It is a new mystery and interesting subject. We found 21 toxin genes using MS/MS of *M*. *martensii* venom samples separated by 2-DE, SDS-PAGE, and RP-HPLCa by Xu et al. (2014) [21]. This suggests the biological significance of the differential expression of toxin genes.

We found that the difference in the expression of sodium channel toxin genes (Figure 6c) among different populations was conserved (populations conservation), suggesting that the expression characteristics of toxins are related to the geographical distribution and genetic relationship, whereas the expression difference in potassium channel toxin genes (Figure 6d) and calcium channel toxin genes (Figure 7d) between the two sexes was conserved (gender conservation), revealing the sexual dimorphism of toxin expression. In addition, sexual dimorphism of toxin gene expression was observed in the sexual venom gland transcriptomes of *M. eupeus* (Figure 6c,d and Figure 7d,f), suggesting that sexual dimorphism of toxin gene expression could be a common feature of species belonging to the order Scorpiones. Heat maps showed differences in the gene expression of calcium channel toxin genes, chloride channel toxin genes, and defensin genes in different sexes of *M. martensii* and *M. eupeus*, although there were too few genes (2–4) available for comparison. Hierarchical clustering did not reflect accurate results about the relationship of interspecific and intraspecific differences such as those of the toxin genes encoding for sodium channels and potassium channels. Interestingly, the expression of two calcium channel toxin genes showed preference for males and certain populations.

It was reported that 51 toxin genes encoding for sodium and potassium channels of *M*. *martensii* were linked into up to 17 gene clusters [20,28]. In this study, eight clusters of toxin genes were found to be not expressed under normal conditions and nine clusters were expressed. Among these, the expression of a member of one gene cluster is consistent in different populations or genders. In addition, the level of expression of each member of the same gene cluster was generally inconsistent, including certain members was not expressed. The expression characters of potassium channel toxin gene clusters and sodium channel toxin gene clusters are the same approximately; however, certain gene members vary in different sexes. This revealed selective silencing, low expression, or high expression among gene cluster members.

It is worth noting that it is not uncertain whether the expression level data of toxin genes and antimicrobial peptide gene families of different populations or sexes of *M*. *martensii* from different localities are significant, which may be the major weakness of this paper to accurately reveal intraspecific differentiation. The data of gene expression levels used for hierarchical clustering of the different populations or sexes did not yield significantly different results in the t-test, even if the standardization process was performed in advance. This may be due to excessive differences in expression levels among the genes in each population or each sex of one population (Supplementary Information S2). However, as we have observed, the difference of color characters among some populations is noticeable and the sexual dimorphism of morphological structures in each population is apparent. The body length of adult individuals from arid and sub-arid areas are smaller than those from sub-wet areas, and the average values of the number of pectinal teeth and the number of large granules in the lateral sides of the pedipalp chela fingers were smaller in the former. By Kruskal–Wallis H test (KW test), there were significant differences in body length, number of pectinal teeth, and number of large granules in the lateral sides of movable and fixed fingers between males or females of *M*. *martensii* from different localities (*p* < 0.001; Tables S1 and S2). Therefore, in terms of morphological characteristics, the populations we selected are significantly different. Due to the wide distribution range and complex habitat of this species, a more extensive sampling is clearly needed for rigorously examining intraspecific diversification and evolution in *M*. *martensii* [12]. Intraspecific resting metabolic rate, a key physiological trait linked with evolutionary fitness, shows that variation exists in between sexes and among populations of *M*. *martensii* that is closely related to the local mean temperature and mean annual days of rainfall [13]. Our findings in the expression differences of toxin gene families between genders and among populations of *M*. *martensii* suggest a similar case to the relevance to the environment to resting metabolic rate.

## 4. Materials and Methods

### 4.1. Collection of Mesobuthus martensii

**Figure 10:**
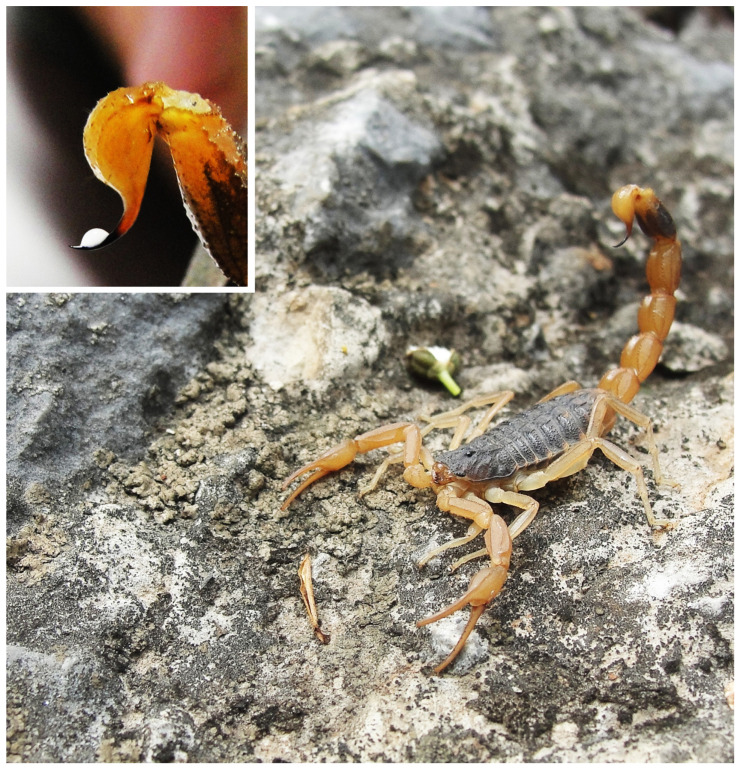
*Mesobuthus martensii* from Nan yang, Henan Province, China; the small picture is its venom gland and venom.

The specimens of wild *M. martensii* were collected from the Gansu Province, Hebei Province, Henan Province, Ningxia Hui Autonomous Region, Shaanxi Province, and Shanxi Province. The localities of specimens were in the sub-wet zone (light green area), sub-arid zone (light yellow area), and arid zone (yellow area) (Figure 1, Figure 10 and Figure S1) and were kept in 75% alcohol. Specimens were deposited in the Museum of Hebei University (MHBU), Baoding, China. The living specimens were raised in the laboratory at room temperature (about 25 °C) temporarily. Geographical names and their abbreviations are also used to represent populations, except when introducing geographical locations and explaining distance relationships between localities.

### 4.2. Morphological Study

Measurements were taken using the Motic-K700 microscope (Motic China Group Co., Ltd., Xiamen, China, with accurate micrometric ocular). All materials were randomly selected from healthy adult individuals. All measurements were in millimeters. The terminology followed was described by Hjelle (1990) [29]. The measurement methods followed were those described by Sissom et al. (1990) [30]. Photographs were acquired by the macro camera Canon 650D (with a microlens). The Division map of arid and wet areas in China is based on Zhang et al. (2016) [31]. The dry humidity division of China followed the procedure described by the National Meteorological Information Center [32].

The body length, number of pectinal teeth, and number of large granules in the lateral sides of the pedipalp chela fingers of randomly selected adult individuals were measured. Kruskal–Wallis H test in SPSS24.0 (IBM, New York, NY, USA) software was used to analyze the overall differences of the characteristics of the scorpions from different localities according to the characteristic values of the scorpions.

### 4.3. Genetic Distance Calculation

We downloaded the COI sequence of wild *M. martensii* from 44 localities uploaded by Shi et al. (2013) in NCBI [12,33]. The coverage area of these localities included the collection sites of *M. martensii*. Next, sequence alignment was performed using ClustalX1.83 (Bioedit Company, IN, USA), and genetic distance was analyzed using MEGA6.0 (Tamura et al., 2013) [34].

### 4.4. Transcriptome Extraction, Sequencing, Annotation, and Gene Analysis

We used pooled scorpion venom glands for the study rather than replicates of individuals or replicates of pools. The number of adult individuals used for each pool (transcriptome) was as followed: *M. martensii*: 1-BD, 4 females and 4 males from Baoding; 2-HL, 2 females and 1 male from Helan; 3-LYF, 9 females from Luoyang; 3′-LYM, 9 males from Luoyang; 4-LZF, 7 females from Lanzhou; 5-SD, 8 females and 3 males from Suide; 6-SZF, 9 females from Shuozhou; 6′-SZM, 9 males from Shuozhou; 7-TS, 5 females and 3 males from Tianshui; 8-WNF, 9 females from Weinan; 8′-WNM, 7 males from Weinan; 9-WZF, 11 females from Wuzhong; 10-YC, 5 females and 5 males from Yuncheng. *M*. *eupeus*: 11-MEF, 9 females from Yinchuan; 11′-MEM, 10 males from Yinchuan. The relevant results of expression of toxin genes in the following text are mean toxin gene expression.

The transcriptomes were extracted by the Trizol method, and the quality of the extracted nucleic acid was determined. Sequencing and splicing were performed using the BGISEQ-500 analysis platform of BGI (Beijing Genomics Institute, Beijing, China), whereas its annotation was done by referring to the genome of *M. martensii* [35]. We defined expressed genes using the following criteria: FPKM ≥ 10 in any one population (FPKM not less than 10 in at least one population). Unless otherwise stated, the unit of expression level in our analyses is FPKM. Online BLAST annotated possible new genes and named them according to the discovery and verification information of *M. martensii* toxin genes provided by Cao et al. (2013) [20]. The heat maps were produced online by BGI. Based on the results of the detection of differential genes, a hierarchical cluster analysis was performed on concatenated differential genes, using the heatmap in R [36]. The gene cluster analysis was based on the method described by Cao et al. (2013) [20].

### 4.5. Proteomic Identification of Expressed Toxin Genes

The peptide sequence of expressed toxin genes was aligned with the MS/MS identification protein evidence of *M. martensii* venom samples separated by 2-DE, SDS-PAGE, and RP-HPLC [21,24] by ClustalX1.83, as well as the local blast.

### 4.6. RT-qPCR Analysis

RT-qPCR analysis was performed to detect the expression of 10 genes in scorpion venom gland tissues. The RT-qPCR primers were synthesized at the Invitrogen Company (Beijing, China). Scorpion GAPDH was used as the housekeeping gene. PCR amplification was conducted in an ABI 7500 real-time PCR system using the following program: 30 s at 95 °C; 40 cycles (95 °C for 5 s, 60 °C for 40 s [collecting fluorescence]). To establish the melting curve of PCR products, amplification of the product was done at 95 °C for 10 s, 60 °C for 60 s, 95 °C for 15 s. They were subsequently slowly heated from 60 °C to 99 °C (ramp rate is 0.05 °C/s). Data were analyzed using Ct values and the 2^−ΔΔCT^ method [37].

## Data Availability

Not applicable.

## Supplementary Materials

The following supporting information can be downloaded at: https://www.mdpi.com/article/10.3390/toxins14090630/s1, Figure S1: The *Mesobuthus martensii* populations and its relatives from sub-wet area, sub-arid area, and arid area. The populations of *M*. *martensii*: 1 & 1’, the females and males from Baoding; 2&2’, the females and males from Helan; 3&3’, the females and males from Luoyang; 4&4’, the females and males from Lanzhou; 5&5’, the females and males from Suide; 6&6’, the females and males from Shuozhou; 7&7’, the females and males from Tianshui; 8&8’, the females and males from Weinan; 9&9’, the females and males from Wuzhong; 10&10, the females and males from Yuncheng. The populations of *M. eupeus*: 11&11’, the females and males from Yinchuan; 12&12’, the females and males from Zhongwei (between Lanzhou and Yinchuan). Table S1: Measurement data of the male *Mesobuthus* *martensii* (means ± SD, **: *p* < 0.001, Kruskal-Wallis H test (KW test)) Body length (BL), number of adult individuals (NI), number of pectinal teeth (NP), number of large granules in the lateral sides of movable fingers (NM), number of large granules in the lateral sides of fixed fingers (NF). BD, Baoding; HL, Helan; LY, Luoyang; LZ, Lanzhou; SD, Suide; SZ, Shuozhou; TS, Tianshui; WN, Weinan; WZ, Wuzhong; YC, Yuncheng. Table S2: Measurement data of the female *Mesobuthus* *martensii* (means ± SD, **: *p* < 0.001, Kruskal-Wallis H test (KW test)) Body length (BL), number of adult individuals (NI), number of pectinal teeth (NP), number of large granules in the lateral sides of movable fingers (NM), number of large granules in the lateral sides of fixed fingers (NF). BD, Baoding; HL, Helan; LY, Luoyang; LZ, Lanzhou; SD, Suide; SZ, Shuozhou; TS, Tianshui; WN, Weinan; WZ, Wuzhong; YC, Yuncheng. Supplementary information S1: The genetic distance of COI sequences of 44 populations of *Mesobuthus martensii* in China is 0.2% to 6.2%. These 44 localities cover the representative sites of the distribution area of *M*. *martensii* in China [12], so it supports that the wild populations in this study of *M. martensii* belong to the same species. Supplementary information S2: The FPKM of toxin and defensin genes of different populations and sexes in the gene expression of *Mesobuthus martensii* (MM) and *Mesobuthus eupeus* (ME) in China. The populations of *M. martensii*: 1-BD, Baoding; 2-HL, Helan; LY, Luoyang (3-LYF, the females from Luoyang; 3’-LYM, the males from Luoyang); LZ, Lanzhou (4-LZF, the females from Lanzhou); 5-SD, Suide; SZ, Shuozhou (6-SZF, the females from Shuozhou; 6’-SZM, the males from Shuozhou); 7-TS, Tianshui; WN, Weinan (8-WNF, the females from Weinan; 8’-WNM, the males from Weinan); WZ, Wuzhong (9-WZF, the females from Wuzhong); 10-YC, Yuncheng. The population of *M*. *eupeus*: ME, Yinchuan (11-YCF, the females of from Yinchuan; 11’-YCM, the males from Yinchuan). The genes with green shadow are that have the MS/MS identification evidences of the *M*. *martensii* venom samples separated by 2-DE, SDS-PAGE and RP-HPLCa by Xu et al. (2014) [21]. Supplementary information S3: S3.1 The multiple sequence alignment of four putative new toxin genes (MMa12627 (BmKNaTx62), MMa29116 (BmKNaTx63), MMa34629 (BmKNaTx64) and MMa38588 (BmKNaTx65)), two predicted defensin genes (MMa09285 (BmKDfsin7) and MMa39355 (BmKDfsin8)) and their homologous genes; S3.2 The protein evidences of expressed toxin genes; S3.3 The peptide sequences with serial numbers in S3.2 from MS/MS identification of the *M*. *martensii* venom samples separated by 2-DE, SDS-PAGE and RP-HPLCa (the original data was from Xu et al. (2014)) [21].
