# Supplementary material for: Transcriptome Sequencing and Comparison of Venom Glands Revealed Intraspecific Differentiation and Expression Characteristics of Toxin and Defensin Genes in Mesobuthus martensii Populations
Source: Toxins (Basel). 2022 Sep 11;14(9):630. doi: 10.3390/toxins14090630 (PMC9503625; doi:10.3390/toxins14090630)
Supplement: Supplementary file 1 [file toxins-14-00630-s001.zip › Supplementary Figure S1 Table S1 Table S2.pdf]

Article

Transcriptome Sequencing and Comparison of Venom Glands Revealed Intraspecific Differentiation and Expression Characteristics of Toxin and Defensin Genes in *Mesobuthus martensii* Populations

Zhiyong Di, Sha Qiao, Xiaoshuang Liu, Shuqing Xiao, Cheng Lei, Yonghao Li, Shaobin Li and Feng Zhang

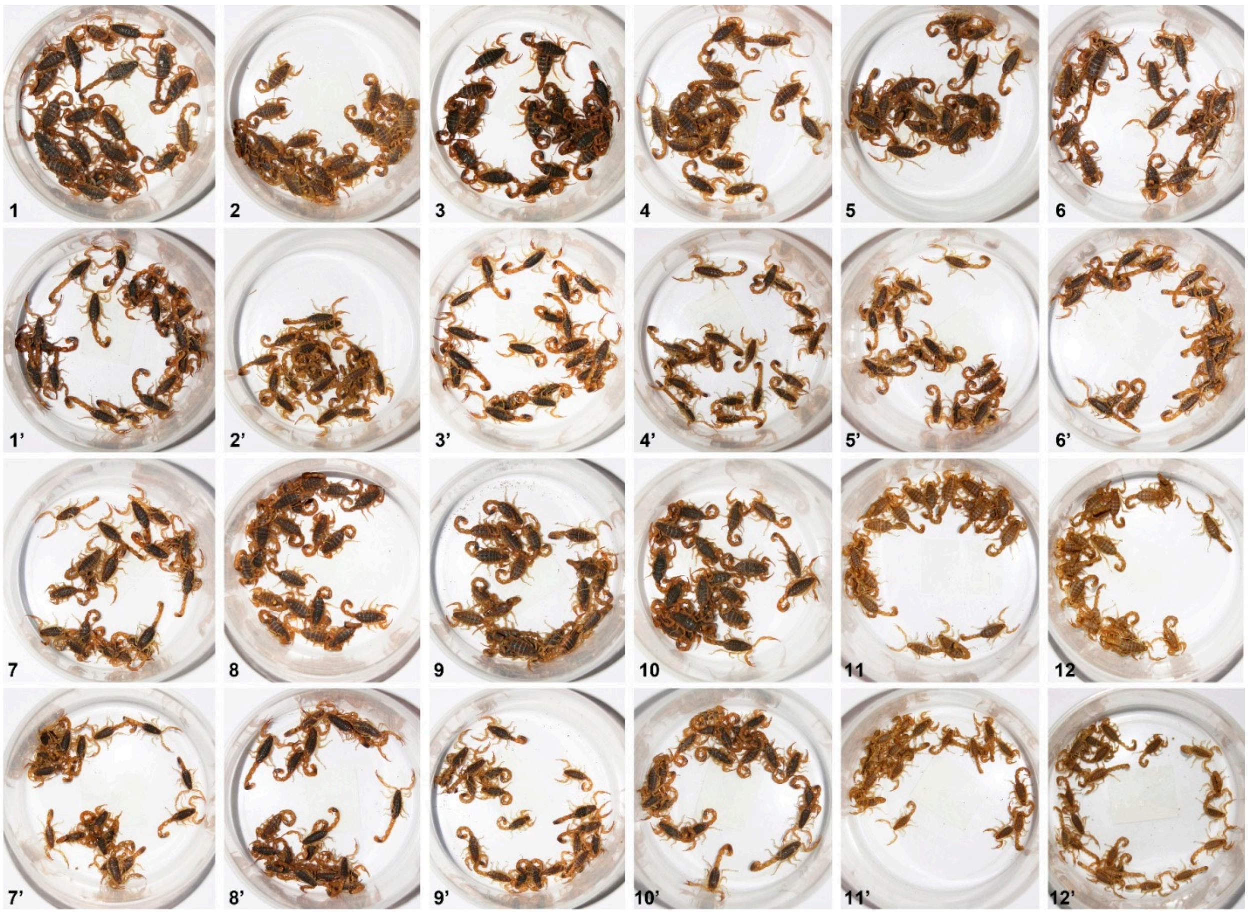

Figure S1. The *Mesobuthus martensii* populations and its relatives from sub-wet area, sub-arid area, and arid area. The populations of *M. martensii*: 1 & 1′, the females and males from Baoding; 2&2′, the females and males from Helan; 3&3′ , the females and males from Luoyang; 4&4′, the females and males from Lanzhou; 5&5′, the females and males from Suide; 6&6′, the females and males from Shuozhou; 7&7′, the females and males from Tianshui; 8&8′, the females and males from Weinan; 9&9′, the females and males from Wuzhong; 10&10, the females and males from Yuncheng. The populations of *M. eupeus*: 11&11′, the females and males from Yinchuan; 12&12′, the females and males from Zhongwei (between Lanzhou and Yinchuan).

Table S1. Measurement data of the male *Mesobuthus martensii* (means±SD, **: $p < 0.001$, Kruskal-Wallis H test (KW test)) Body length (BL), number of adult individuals (NI), number of pectinal teeth (NP), number of large granules in the lateral sides of movable fingers (NM), number of large granules in the lateral sides of fixed fingers (NF). BD, Baoding; HL, Helan; LY, Luoyang; LZ, Lanzhou; SD, Suide; SZ, Shuozhou; TS, Tianshui; WN, Weinan; WZ, Wuzhong; YC, Yuncheng.

	WN	LY	BD	YC	TS	SZ	SD	LZ	WZ	HL	KW test	
NI	66	48	32	34	56	59	46	44	22	36	*p*	
BL	54.200±3.615	55.600±3.836	52.400±4.274	50.100±3.035	50.400±1.265	51.900±3.604	48.500±2.014	48.700±2.497	49.222±2.224	49.500±2.121	.000	**
NP	25.091±1.200	25.375±1.214	23.094±1.376	26.500±1.562	24.268±1.272	22.746±1.169	24.304±1.051	24.909±1.074	24.545±1.224	24.750±1.519	.000	**
NM	13.529±.657	13.727±.660	13.375±.492	13.588±.701	12.873±.695	12.932±.487	13.109±.674	12.933±.495	12.682±.568	12.778±.485	.000	**
NF	11.328±0.561	11.432±.501	10.871±.500	11.588±0657	10.745±.552	10.967±.367	10.978±.500	10.609±.493	10.636±.492	10.750±.500	.000	**

Table S2. Measurement data of the female *Mesobuthus martensii* (means±SD, **: $p < 0.001$, Kruskal-Wallis H test (KW test)) Body length (BL), number of adult individuals (NI), number of pectinal teeth (NP), number of large granules in the lateral sides of movable fingers (NM), number of large granules in the lateral sides of fixed fingers (NF). BD, Baoding; HL, Helan; LY, Luoyang; LZ, Lanzhou; SD, Suide; SZ, Shuozhou; TS, Tianshui; WN, Weinan; WZ, Wuzhong; YC, Yuncheng.

	WN	LY	BD	YC	TS	SZ	SD	LZ	WZ	HL	KW test	
NI	46	47	39	57	35	52	58	61	54	42	*p*	
BL	60.700±2.058	58.700±2.830	63.300±3.401	59.800±5.138	52.200±2.348	57.700±3.592	55.300±2.263	55.600±2.503	56.200±2.530	54.400±1.633	.000	**
NP	20.674±.871	20.213±.977	19.410±.966	21.246±1.040	20.886±1.451	18.827±.834	20.362±.831	20.475±1.074	19.981±.900	20.167±1.034	.000	**
NM	13.521±.652	13.604±.536	13.538±.822	13.436±.631	12.861±.487	12.870±.702	13.172±.653	12.883±.555	12.759±.642	12.952±.539	.000	**
NF	11.391±.537	11.292±.544	11.026±.628	11.526±.601	10.914±.374	10.750±.622	11.207±.554	10.629±.683	10.704±.571	11.024±.474	.000	**
