# Supplementary material for: Transcriptome Sequencing and Comparison of Venom Glands Revealed Intraspecific Differentiation and Expression Characteristics of Toxin and Defensin Genes in Mesobuthus martensii Populations
Source: Toxins (Basel). 2022 Sep 11;14(9):630. doi: 10.3390/toxins14090630 (PMC9503625; doi:10.3390/toxins14090630)
Supplement: Supplementary file 1 [file toxins-14-00630-s001.zip › Supplementary Information S3.pdf]

Supplementary Information S3

S3.1 The multiple sequence alignment of four putative new toxin genes (MMa12627 (BmKNaTx62), MMa29116 (BmKNaTx63), MMa34629 (BmKNaTx64) and MMa38588 (BmKNaTx65)), two predicted defensin genes (MMa09285 (BmKDfsin7) and MMa39355 (BmKDfsin8)) and their homologous genes

S3.2 The protein evidences of expressed toxin genes

S3.3 The peptide sequences with serial numbers in S3.2 from MS/MS identification of the *M. martensii* venom samples separated by 2-DE, SDS-PAGE and RP-HPLCa (the original data was from Xu et al. (2014)) [21]

S3.1 The multiple sequence alignment of four putative new toxin genes (MMa12627 (BmKNaTx62), MMa29116 (BmKNaTx63), MMa34629 (BmKNaTx64) and MMa38588 (BmKNaTx65)), two predicted defensin genes (MMa09285 (BmKDfsin7) and MMa39355 (BmKDfsin8)) and their homologous genes

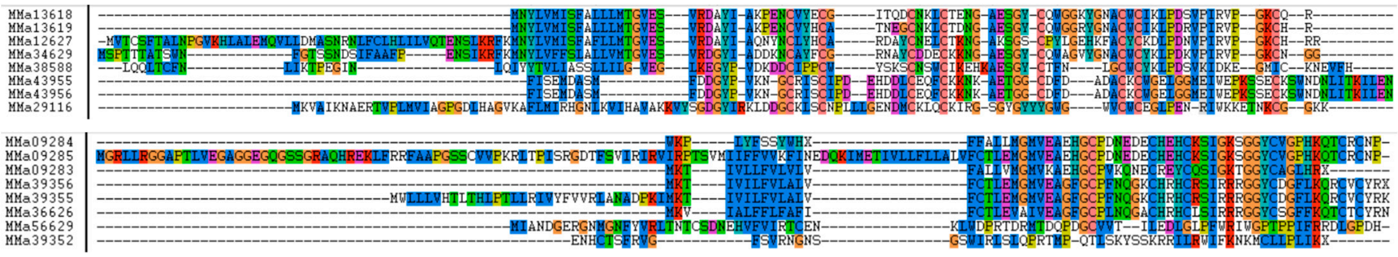

S3.2 The protein evidences of expressed toxin genes

Twenty-one expressed genes with the MS/MS identification evidences of the *M. martensii* venom samples separated by 2-DE, SDS-PAGE and RP-HPLCa by Xu et al. (2014) [21]. Five genes with yellow shadow have the inconclusive evidences due to the inconsistency of an amino acid residue between the multiple alignment sequences of putative toxin genes and the peptide sequences from Xu et al. (2014) [21]

Name **Gene ID**

BmKNaTx3 MMa13619

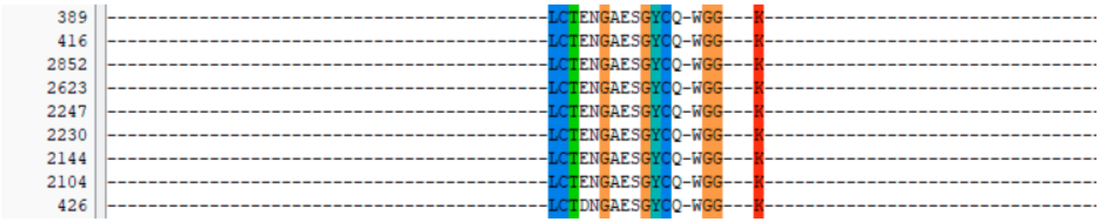

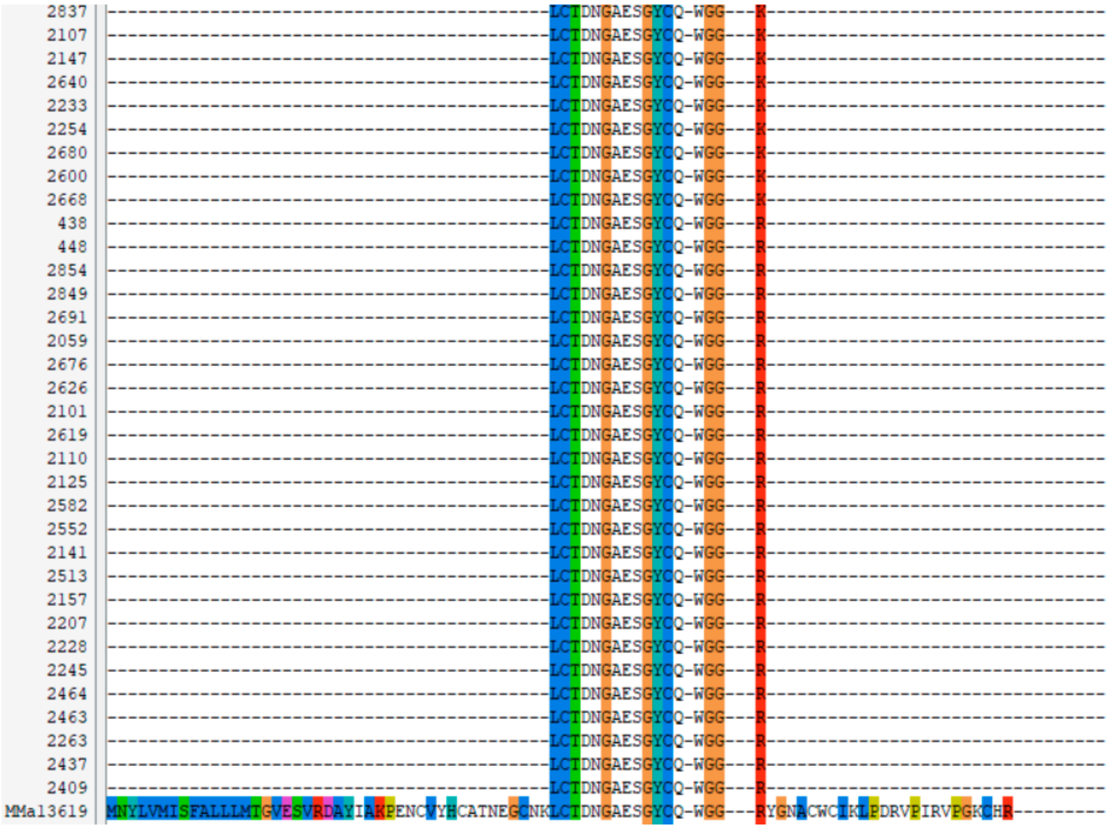

BmKNaTx2 MMa13616

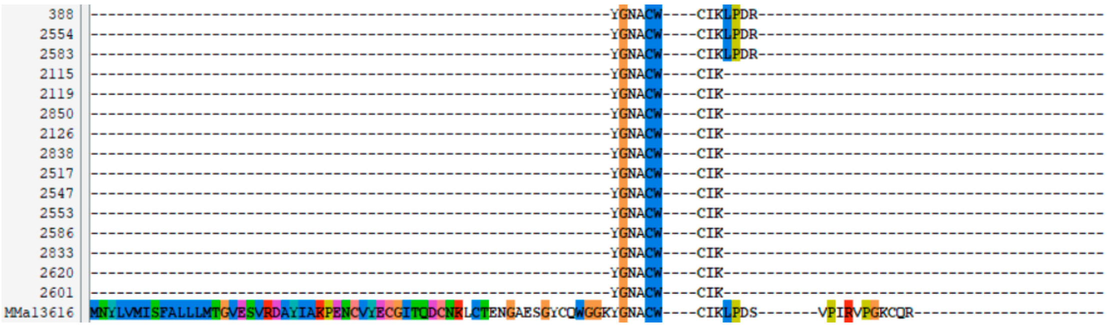

BmKNaTx4 MMa17865

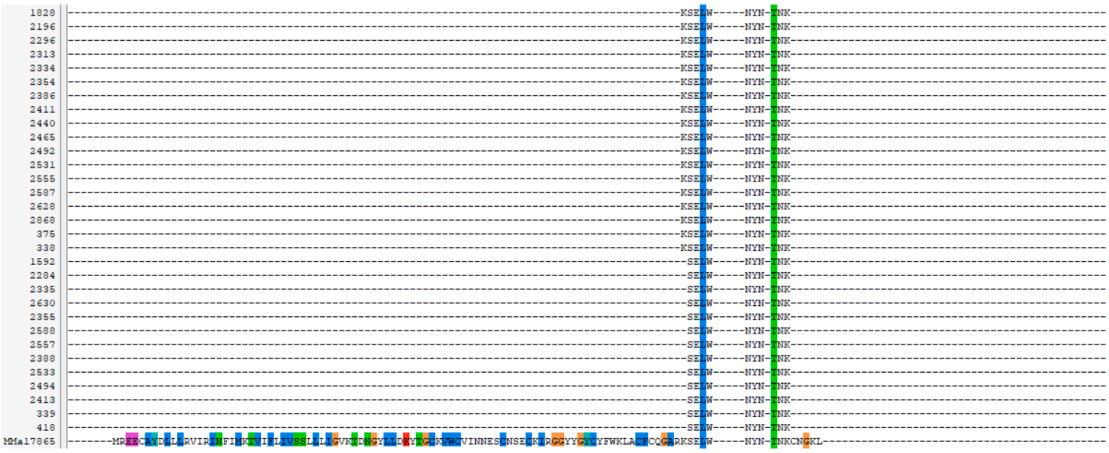

BmKNaTx5 MMa17864

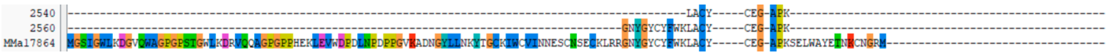

BmKNaTx6 MMa17854

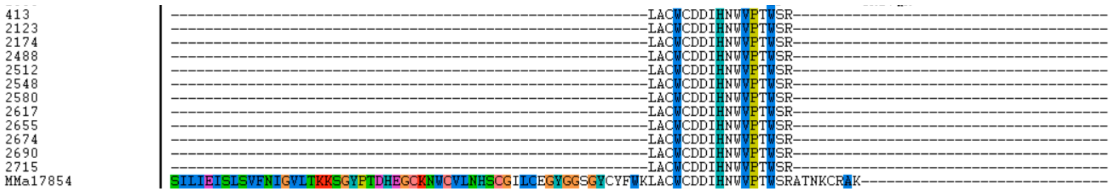

BmKNaTx7 MMa17853

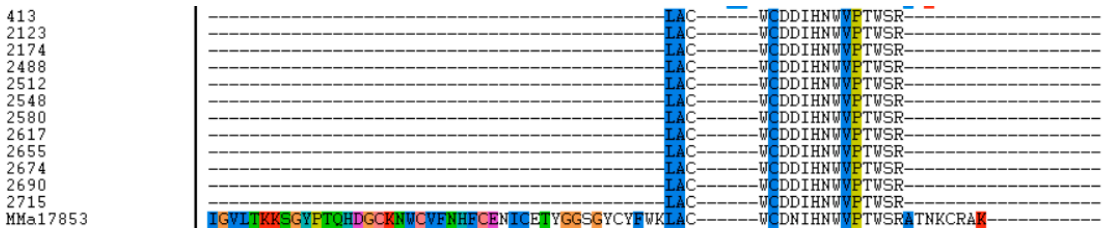

BmKNaTx8 MMa17863

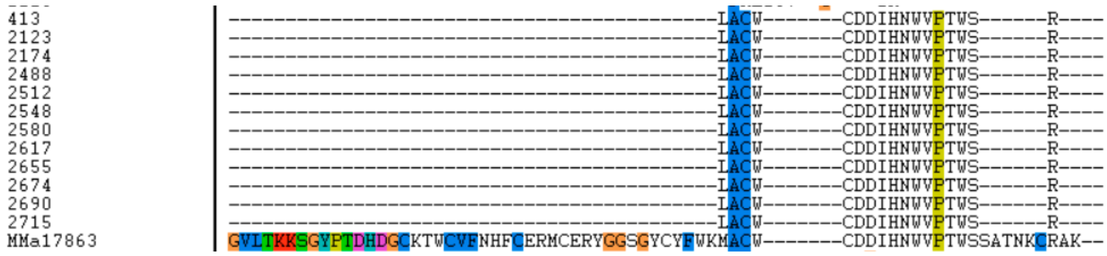

BmKNaTx11 MMa29117

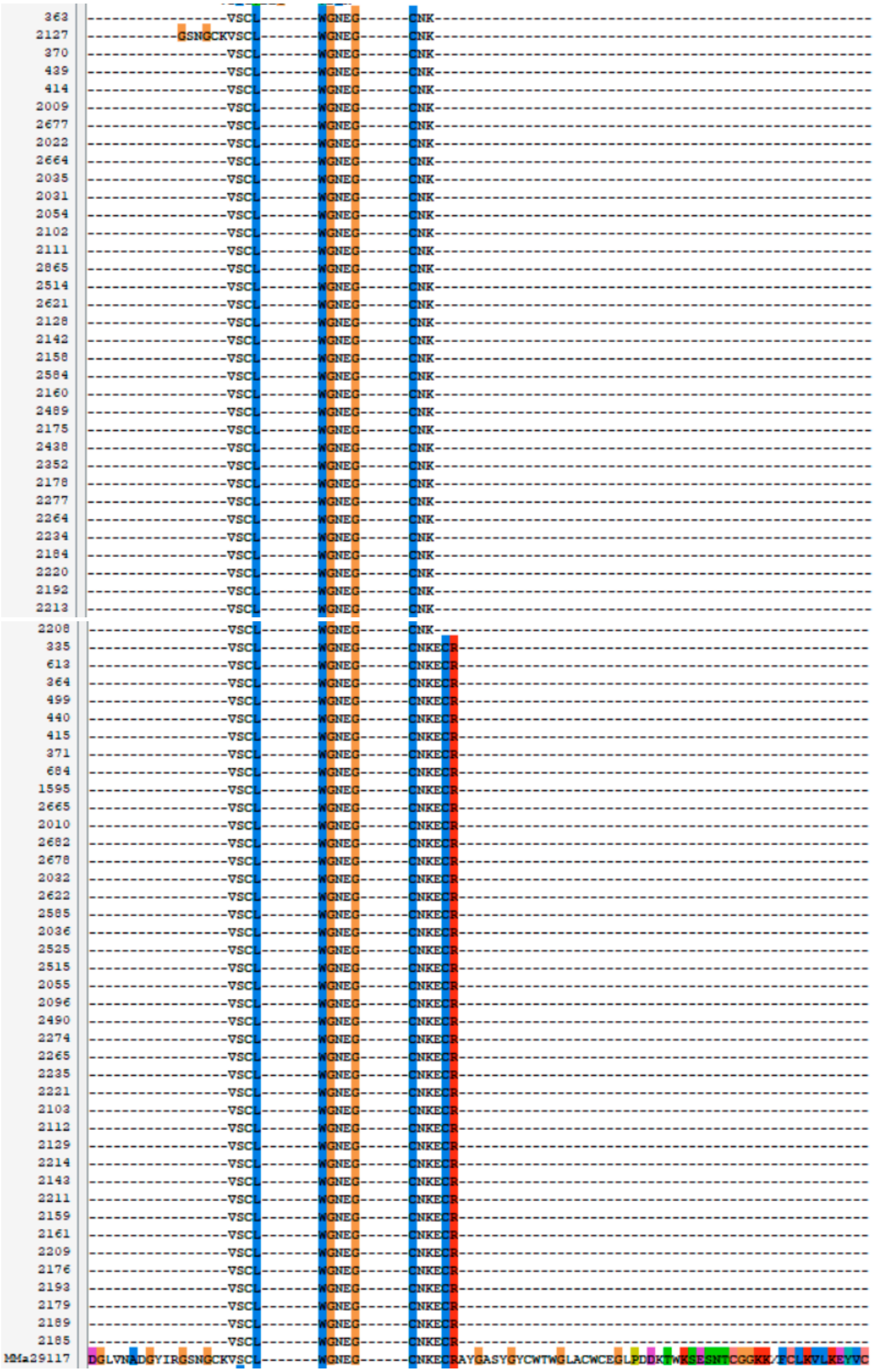

BmKNaTx15 MMa32661

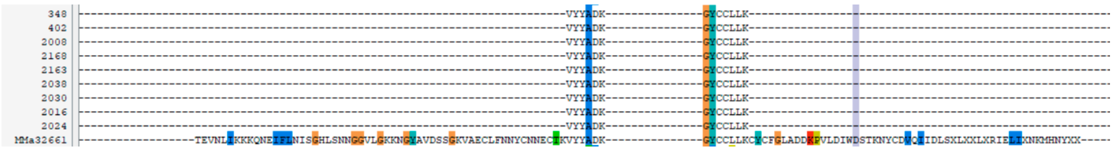

BmKNaTx17 MMa32663

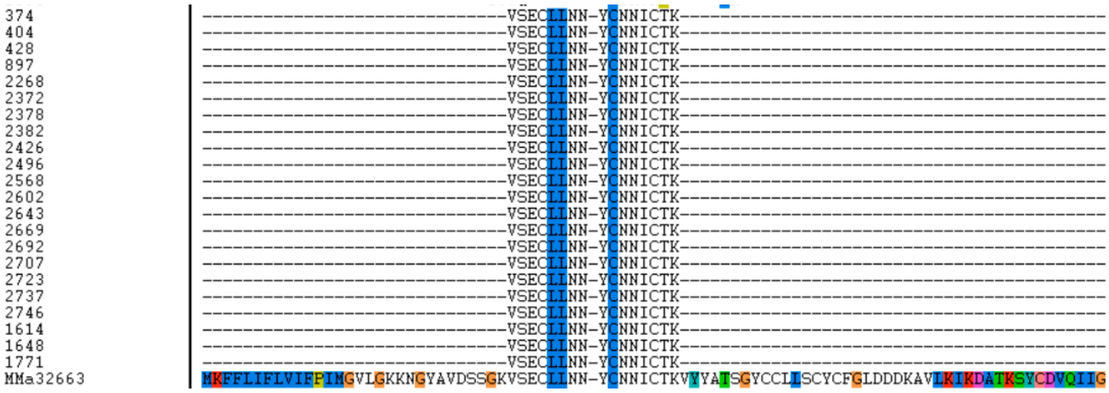

BmKNaTx22 MMa37864

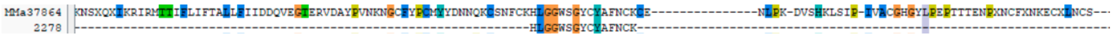

BmKNaTx23 MMa43956

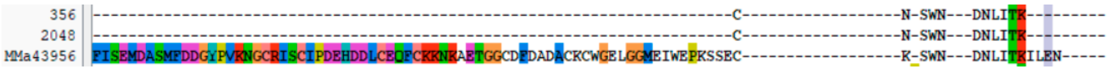

BmKNaTx24 MMa43955

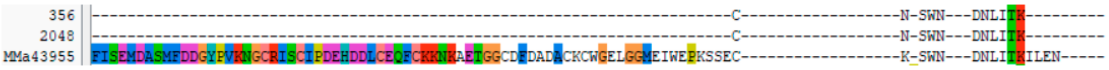

BmKNaTx31 MMa55372

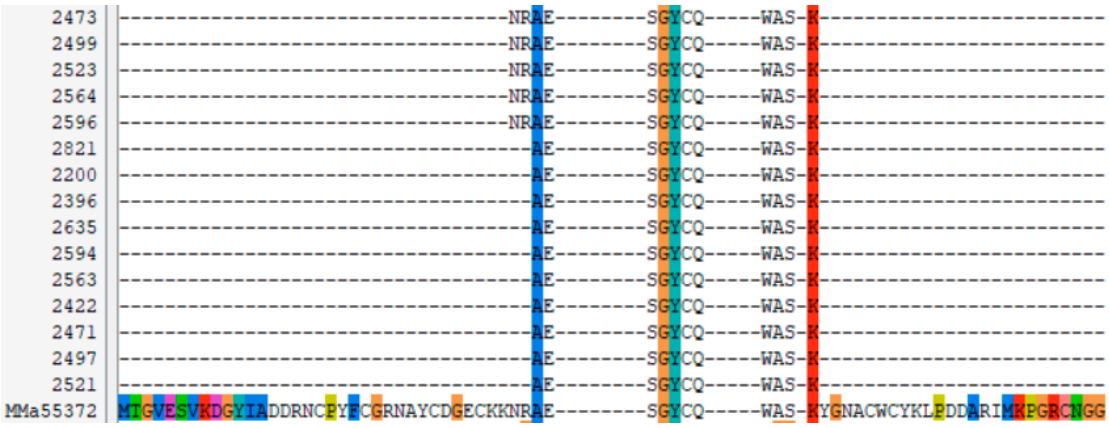

BmKNaTx40 MMa13212

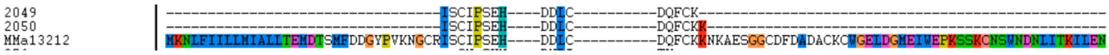

BmKNaTx42 MMa20191

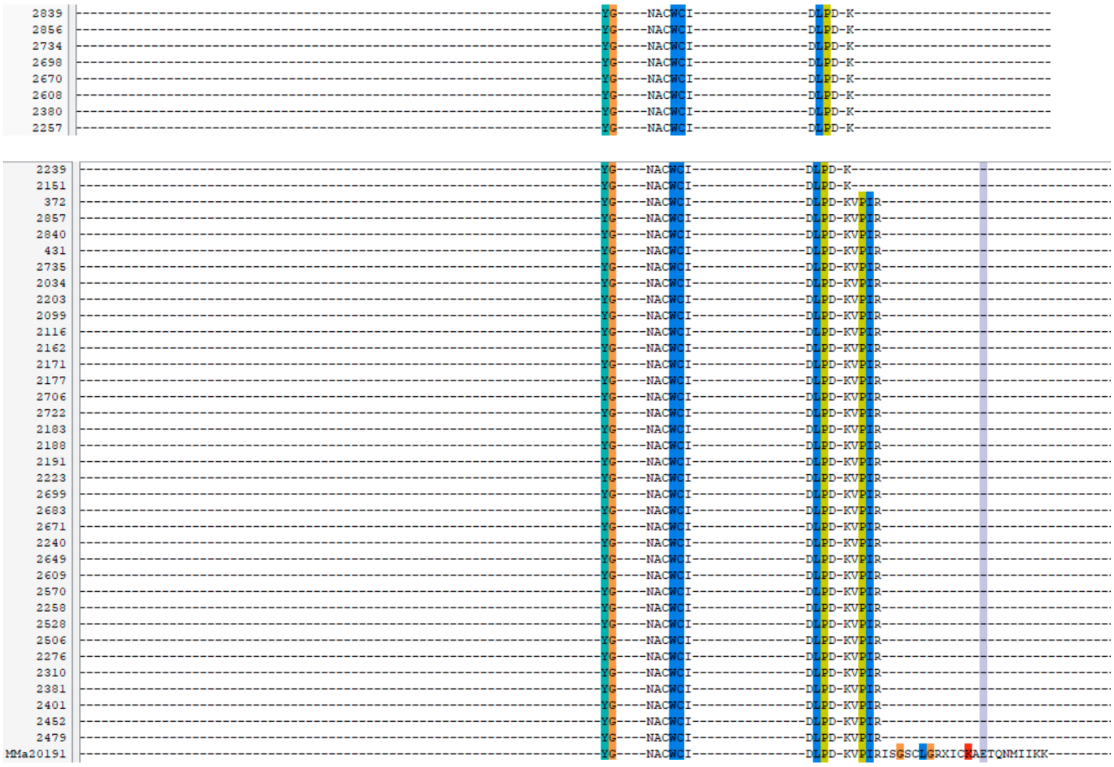

BmKNaTx44 MMa23370

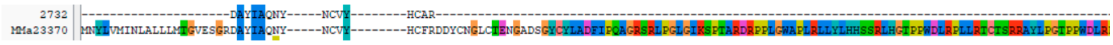

BmKNaTx54 MMa55680

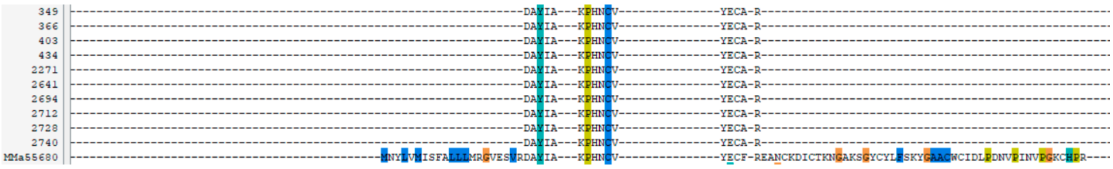

BmKNaTx62 MMa12627

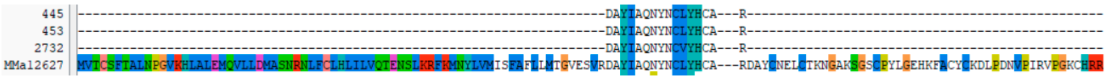

BmKNaTx64 MMa34629

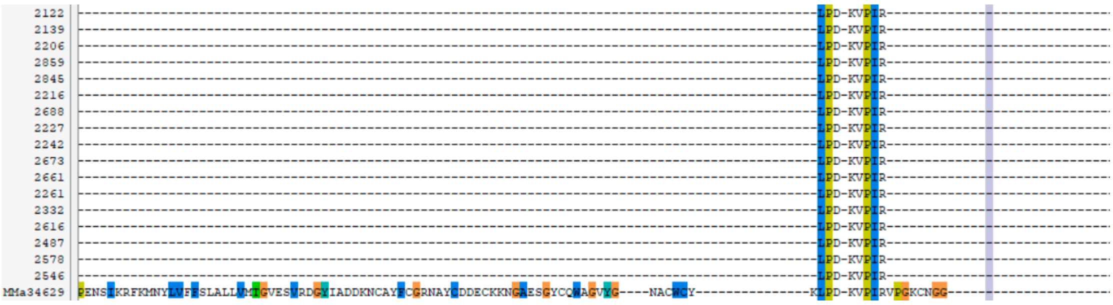

BmKaKTx1MMa16285

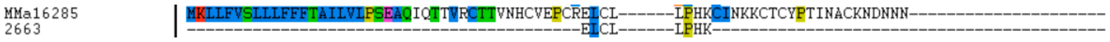

BmKaKTx10 MMa35044

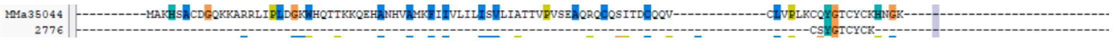

BmKaKTx28 MMa23444

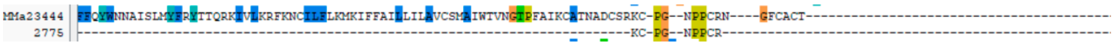

BmKbKTx2 MMa35531

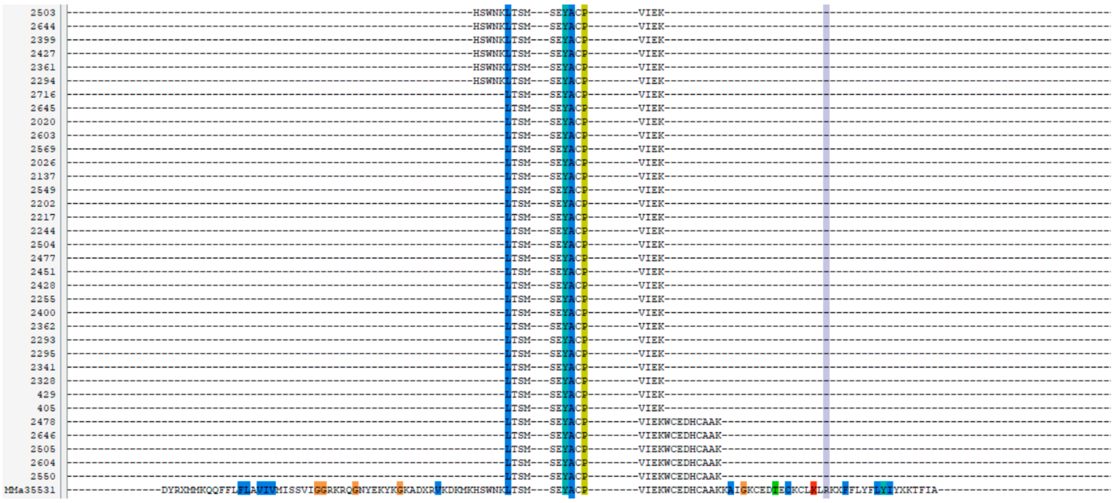

BmKClTx2 MMa44674

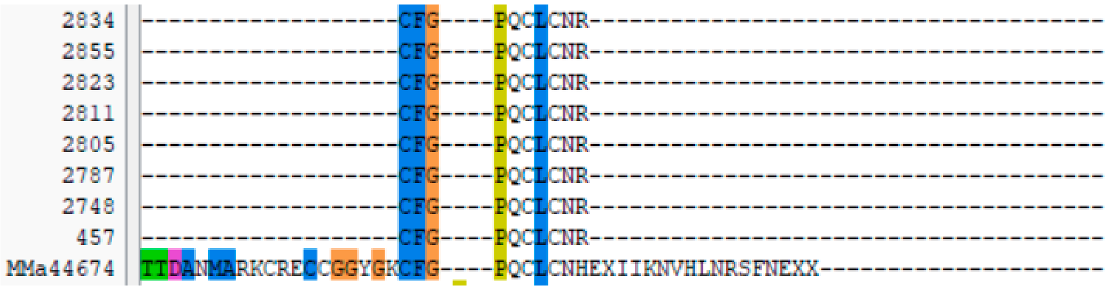

BmKClTx3 MMa00982

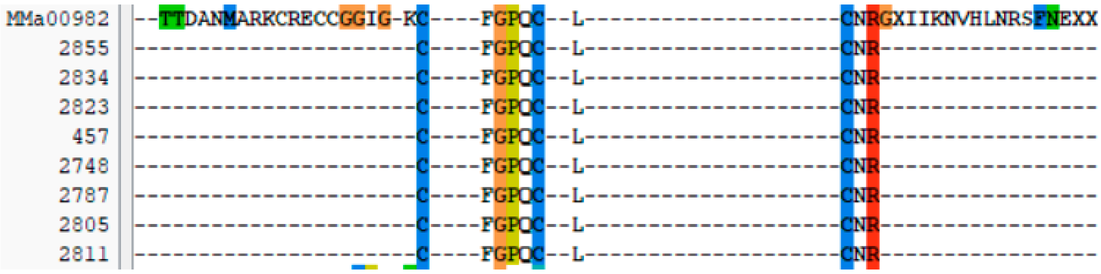

S3.3 The peptide sequences with serial numbers in S3.2 from MS/MS identification of the *M. martensii* venom samples separated by 2-DE, SDS-PAGE and RP-HPLCa (the original data was from Xu et al. (2014)) [21]

Name	Sequence ID	Peptide sequence
>1	>MMa46238	YKNPPYENYNVPPILDNYQK
>2	>MMa42691	AMEDGIVYSGK
>3	>MMa31039	IISNEELNVIAGDVSPGK
>4	>comp156_c0_seq1_6	LFSISEPIKCK
>5	>comp120_c0_seq1_2	CIAGPEINCDSEIEGK
>6	>MMa21615	GNRFSYDGNYQDGSR
>7	>comp214_c0_seq1_6	AGPSSLVAIR
>8	>comp214_c0_seq1_6	NTESTFYAFGR
>9	>comp188_c0_seq1_3	SVEDFANSWK
>10	>comp188_c0_seq1_3	SVEDFANSWKK
>11	>comp20_c0_seq1_3	YAVPEGTLR
>12	>comp52_c1_seq1_5	GAPAQYLDVNYVR
>13	>MMa19079	YIDVDQNELTR
>14	>comp197_c0_seq1_4	TYVKDVIAPVASTVIQNVNR
>15	>comp11260_c0_seq1_6	ENKHLVTR
>16	>comp61_c0_seq1_2	GIAAEETLKNMAK
>17	>comp61_c0_seq1_2	SNEANLAPYIPSQPR
>18	>comp197_c0_seq1_4	LSHPNTLIYPYINYILPGTKK
>19	>comp158487_c0_seq1_6	QSTDLIPFIDSVTACSFIENTPK
>20	>comp627_c0_seq1_2	ADLSNNLITR
>21	>comp627_c0_seq1_2	LVLDDNSIAR
>22	>comp64616_c0_seq1_1	FVENNEPQTFFK
>23	>comp64616_c0_seq1_1	VAAEQFYEKVEQETSK
>24	>comp416_c0_seq3_3	EIAKVAENFYR
>25	>comp416_c0_seq3_3	GLCLASAQSGLFHR
>26	>comp416_c0_seq3_3	LYKEIMPFYK
>27	>comp416_c0_seq3_3	NFFGGILQFQFHK
>28	>comp83009_c0_seq1_3	IKIDADGNLGER
>29	>comp73628_c0_seq1_4	EICYSPDALAGYCTIHGMK
>30	>MMa40622	SHELKLMIPR
>31	>comp188_c0_seq1_3	IFNNPTFLNCK
>32	>comp188_c0_seq1_3	IYIDAMPTLYNQLK
>33	>comp188_c0_seq1_3	LYDRVEMNGLECK
>34	>comp188_c0_seq1_3	NLFDCANGGNCR
>35	>comp188_c0_seq1_3	SVEDFANSWKK
>36	>comp188_c0_seq1_3	VDIPDDITVFWDRNNR
>37	>comp188_c0_seq1_3	YSCASSAMESLVR
>38	>comp93900_c0_seq1_5	NQEIFGDKIEDIPDYNIER
>39	>comp1172_c0_seq1_1	NQIDSIDDSLYGLTK

>40	>comp1172_c0_seq1_1	NQIDSIDDSLYGLTKLR
>41	>MMa37755	EAVKNLPHEFVAALR
>42	>MMa37755	EGGIISVNNVGLKVLCDTQR
>43	>MMa37755	SPVFYFLPHINKEILFNR
>44	>MMa27598	ADIITLDGGDVYR
>45	>MMa27598	ASSLQQSPSIYLGDFR
>46	>MMa27598	SLELSSLHELEGKR
>47	>comp197_c0_seq1_4	LSHPNTLIYPYINYILPGTK
>48	>comp197_c0_seq1_4	LSHPNTLIYPYINYILPGTKK
>49	>MMa42691	DASPQYFEFKEYGVR
>50	>comp1157_c0_seq1_1	ILATVSNSRDFAK
>51	>comp1157_c0_seq1_1	NAVNDMVIR
>52	>comp1157_c0_seq1_1	QYDQFYIPELHR
>53	>comp1157_c0_seq1_1	VFYDVHPPAR
>54	>comp627_c0_seq1_2	ADLSNNLITR
>55	>MMa52178	EAILDDAQYVPDPK
>56	>MMa52178	NNIVLLANILSK
>57	>comp544_c0_seq1_5	LTLVCEDFENINQVHSALR
>58	>comp544_c0_seq1_5	SLNVADNQLVEIGEK
>59	>comp544_c0_seq1_5	SLNVADNQLVEIGEKIK
>60	>comp120_c0_seq1_2	CIAGPEINCDSEIEGKGK
>61	>comp48_c0_seq1_5	LSTPVTFTPHVNR
>62	>MMa55716	QFHIYSFPGDDALTDDKIR
>63	>comp214_c0_seq1_6	AGPSSLVAIR
>64	>comp214_c0_seq1_6	ARNTESTFYAFGR
>65	>comp214_c0_seq1_6	DIDPKESFLHYER
>66	>comp214_c0_seq1_6	DLQNITEEYK
>67	>comp214_c0_seq1_6	DLQNITEEYKR
>68	>comp214_c0_seq1_6	ELPDTAGSKVIEVDSVELYSK
>69	>comp214_c0_seq1_6	EYVLNDTGKIFVGSYSVPK
>70	>comp214_c0_seq1_6	GSPFFVAIR
>71	>comp214_c0_seq1_6	GTLVQLILTNNKEFTKPK
>72	>comp214_c0_seq1_6	ISFTNPLDK
>73	>comp214_c0_seq1_6	ISFTNPLDKK
>74	>comp214_c0_seq1_6	LKPQTEGNTKLVLVFNSR
>75	>comp214_c0_seq1_6	LSINKYQVGR
>76	>comp214_c0_seq1_6	LTDCTISIECPGVTAPYR
>77	>comp214_c0_seq1_6	LVLVFNSR
>78	>comp214_c0_seq1_6	NTESTFYAFGR
>79	>comp214_c0_seq1_6	NYPHTNSENYISYRR
>80	>comp214_c0_seq1_6	RGSPFFVAIR
>81	>comp214_c0_seq1_6	STLPVSFLCK

>82	>comp214_c0_seq1_6	TVTNYVSAHDSDETMTIDHFYDK
>83	>comp214_c0_seq1_6	VIEVDSVELYSK
>84	>comp214_c0_seq1_6	VIEVDSVELYSKQNALEHK
>85	>comp214_c0_seq1_6	VTPEEYYDKLVDYSMMK
>86	>comp20_c0_seq1_3	YAVPEGTLR
>87	>comp52_c1_seq1_5	GAPAQYLDVNYVR
>88	>MMa31039	IISNEELNVIAGDVSPGKGK
>89	>comp551_c0_seq1_1	EITGEYPVNAFLPLK
>90	>comp551_c0_seq1_1	EITGEYPVNAFLPLKHLR
>91	>MMa50472	EAIIDDAQYVPDPK
>92	>MMa50472	NNIVLLANILSK
>93	>comp61_c0_seq1_2	GIAAEETLKNMAK
>94	>comp61_c0_seq1_2	SNEANLAPYIPSQPR
>95	>MMa47190	TVNYELTGLTNLR
>96	>comp197_c0_seq1_4	LSHPNTLIYPYINYILPGTK
>97	>comp197_c0_seq1_4	LSHPNTLIYPYINYILPGTKK
>98	>comp197_c0_seq1_4	YLIDPKNPTFK
>99	>MMa42691	AMEDGIVYSGK
>100	>MMa42691	DASPQYFEFKEYGVR
>101	>MMa42691	FGPNDASEKPGSFVSFEFSAPGLIFPK
>102	>MMa42691	VIAGGSVSDSDKETILK
>103	>comp41_c0_seq1_5	DANLSLMKDVNNLTYLIK
>104	>MMa31039	IISNEELNVIAGDVSPGK
>105	>comp774_c0_seq1_5	IHSGILPNTLK
>106	>comp774_c0_seq1_5	NVQSLNLAGNQLTEIDESFNCLNK
>107	>comp774_c0_seq1_5	TLDLSHNQIHSLGK
>108	>comp774_c0_seq1_5	TLNDELTGLR
>109	>comp120_c0_seq1_2	CIAGPEINCDSEIEGKGK
>110	>comp48_c0_seq1_5	LSTPVTFTPHVNR
>111	>comp929_c0_seq1_3	EASNHPDQQSIVVEAEETGNILDPEYK
>112	>MMa55716	EFVDLNNEAAR
>113	>MMa55716	LNGFHDAGDFQR
>114	>MMa55716	LNYQGLCPPVKR
>115	>MMa55716	QFHIYSFPGDDALTDDKIR
>116	>MMa55716	TGANPGFHEAIGDVMALSVYTPSHLK
>117	>MMa30097	SEIVEQLPNVR
>118	>comp548_c0_seq1_1	VGILAVDNAVYALR
>119	>MMa53616	FGFGLLDAEAMVK
>120	>MMa53616	GALELYLISPSGSK
>121	>MMa53616	SIFITDSQTQLPQPIATGK
>122	>comp52_c1_seq1_5	GAPAQYLDVNYVR
>123	>comp52_c1_seq1_5	SHSISYLGLCK

>124	>comp227_c0_seq1_5	IINEPTAAAIAYGLDK
>125	>comp227_c0_seq1_5	IINEPTAAAIAYGLDKK
>126	>comp227_c0_seq1_5	MDKSQIHDIVLVGGSTR
>127	>comp227_c0_seq1_5	MKEIAEAYLGK
>128	>comp227_c0_seq1_5	QTQTFTTYSDNQPGVLIQVYEGER
>129	>comp227_c0_seq1_5	TFFPEEISSMVLTK
>130	>comp227_c0_seq1_5	TTPSYVAFTDTER
>131	>comp551_c0_seq1_1	EITGEYPVNAFLPLKHLR
>132	>comp551_c0_seq1_1	LLFLYENQLNSLGNSLIHLK
>133	>comp551_c0_seq1_1	SLNGELTGLINLQELNVK
>134	>MMa49673	ALGDGSYTCPSYFLTR
>135	>MMa39946	LRDPEETFTVFAPVDSAFK
>136	>MMa36925	TAADERPIVVNLEATGNVLDPEYK
>137	>MMa43767	DASSSSVTISHVPTVEELKQGK
>138	>comp61_c0_seq1_2	FSDNTAGIAFPNTVCR
>139	>comp61_c0_seq1_2	GIAAEETLKNMAK
>140	>comp61_c0_seq1_2	SNEANLAPYIPSQPR
>141	>comp544_c0_seq1_5	LTLVCEDFENINQVHSALR
>142	>comp544_c0_seq1_5	SLNVADNQLVEIGEK
>143	>comp1072_c0_seq1_4	ITELKDSLQELR
>144	>comp1072_c0_seq1_4	LGISSNLFNKLDVGK
>145	>comp1072_c0_seq1_4	LIELAEISR
>146	>comp1072_c0_seq1_4	TLTNELTGLK
>147	>comp1072_c0_seq3_6	LYLPLHDLPDTIFANQGNCLR
>148	>comp61_c0_seq1_2	FSDNTAGIAFPNTVCR
>149	>comp61_c0_seq1_2	GIAAEETLKNMAK
>150	>comp61_c0_seq1_2	SNEANLAPYIPSQPR
>151	>MMa31039	IISNEELNVIAGDVSPGK
>152	>MMa31039	IISNEELNVIAGDVSPGKGK
>153	>comp52_c1_seq1_5	GANVAGSNSNNQFEVQLDR
>154	>comp52_c1_seq1_5	GAPAQYLDVNYVR
>155	>comp52_c1_seq1_5	SHSISYLGLCK
>156	>comp52_c1_seq1_5	TVTLNGGETTTLK
>157	>comp52_c1_seq1_5	VGCGYVSFEK
>158	>comp52_c1_seq1_5	VLGERGPDMDEGDFSR
>159	>MMa35583	LIELAEISR
>160	>comp4077_c0_seq1_2	NNQIETLDNSLYNLQELK
>161	>comp4077_c0_seq1_2	NNQIETLDNSLYNLQELKR
>162	>comp4077_c0_seq1_2	TLTNELTDLKNLK
>163	>comp48_c0_seq1_5	IGNIDSDSGQEYTFQSFSK
>164	>comp48_c0_seq1_5	LSTPVTFTPHVNR
>165	>comp48_c0_seq1_5	NSGIDGVCEGDSGGPLVVR

>166	>comp48_c0_seq1_5	NSGIDGVCEGDSGGPLVVRR
>167	>comp431_c0_seq1_5	LFYLYGADPVIGISVAPNDKNTK
>168	>comp431_c0_seq1_5	STFKDSYNQIFDEFGGFPLVK
>169	>comp41_c0_seq1_5	DANLSLMKDVNNLTYLIK
>170	>comp1072_c0_seq4_6	NFEDINQVQNALR
>171	>comp197_c0_seq1_4	AKHPDWSPAQIEK
>172	>comp197_c0_seq1_4	DVIAPVASTVIQNVNR
>173	>comp197_c0_seq1_4	GRGNCVWPEEPYTSWK
>174	>comp197_c0_seq1_4	ILVNQEETFNGDK
>175	>comp197_c0_seq1_4	LSHPNTLIYPYINYILPGTK
>176	>comp197_c0_seq1_4	LSHPNTLIYPYINYILPGTKK
>177	>comp197_c0_seq1_4	NDIQEANDKLSWLWK
>178	>comp197_c0_seq1_4	QSTALCPSIYMQESHITK
>179	>comp197_c0_seq1_4	TYVKDVIAPVASTVIQNVNR
>180	>comp197_c0_seq1_4	VAIEEWENSAK
>181	>comp197_c0_seq1_4	VAIEEWENSAKEWMLK
>182	>comp197_c0_seq1_4	YLIDPKNPTFK
>183	>comp197_c0_seq1_4	YPHIESHGDINGGMLQVSDLANHLK
>184	>MMa42689	FGPNDASEKPGSFVSFEFSAPGLIFPGGSK
>185	>MMa42689	LLAGGSVSDSDKQTILK
>186	>MMa42689	NGICAYLIGR
>187	>MMa42689	TSIISSSWHTIGILMK
>188	>MMa42689	VLYTCNYGPAGNIK
>189	>MMa42691	AMEDGIVYSGK
>190	>MMa42691	AMEDGIVYSGKYK
>191	>MMa42691	APSTDDYLLYCQFSDKDSK
>192	>MMa42691	DASPQYFEFKEYGVR
>193	>MMa42691	FGPNDASEKPGSFVSFEFSAPGLIFPK
>194	>MMa42691	IGCGVAGYTEANTK
>195	>MMa42691	NGICAYLIGR
>196	>MMa42691	TSSSWNTIGILMK
>197	>MMa42691	VIAGGSVSDSDKETILK
>198	>MMa31039	IISNEELNVIAGDVSPGK
>199	>MMa31039	IISNEELNVIAGDVSPGKGK
>200	>MMa31039	AMEDGIVYSGK
>201	>MMa31039	AMEDGIVYSGKYK
>202	>MMa31039	APSTDDYLLYCQFSDKDSK
>203	>MMa31039	DASPQYFEFKEYGVR
>204	>MMa31039	FGPNDASEKPGSFVSFEFSAPGLIFPK
>205	>MMa31039	IGCGVAGYTEANTK
>206	>MMa31039	NGICAYLIGR
>207	>MMa31039	TSSSWNTIGILMK

>208	>MMa31039	VIAGGSVSDSDKETILK
>209	>MMa15302	SYELPDGQVITIGNER
>210	>comp41_c0_seq1_5	DANLSLMKDVNNLTYLIK
>211	>MMa42689	LLAGGSVSDSDKQTILK
>212	>MMa42689	NGICAYLIGR
>213	>MMa42689	TSIISSSWHTIGILMK
>214	>MMa42689	VLYTCNYGPAGNIK
>215	>comp48_c0_seq1_5	IGNIDSDSGQEYTFQSFSK
>216	>comp48_c0_seq1_5	LSTPVTFTPHVNR
>217	>comp48_c0_seq1_5	NSGIDGVCEGDSGGPLVVR
>218	>comp48_c0_seq1_5	NSGIDGVCEGDSGGPLVVRR
>219	>comp545_c0_seq1_1	AGVHLLGTNGAIPAHK
>220	>comp545_c0_seq1_1	IEQPVTLHPFAFR
>221	>comp1072_c0_seq1_4	LIELAEISR
>222	>comp1072_c0_seq1_4	TLTNELTGLK
>223	>comp4077_c0_seq1_2	NNQIETLDNSLYNLQELK
>224	>comp544_c0_seq1_5	GITEDLPINALVPLKNLR
>225	>comp544_c0_seq1_5	SLNVADNQLVEIGEK
>226	>comp1072_c0_seq3_6	LYLPLHDLPDTIFANQGNCLR
>227	>comp52_c1_seq1_5	DEQQTILELHNK
>228	>comp52_c1_seq1_5	DPTGKLPPAGDMLEMEWDEELSK
>229	>comp52_c1_seq1_5	EGVSKDEQQTILELHNK
>230	>comp52_c1_seq1_5	FTPESINPFIDDDATGHFTQMAWSSTWK
>231	>comp52_c1_seq1_5	GANVAGSNSNNQFEVQLDR
>232	>comp52_c1_seq1_5	GAPAQYLDVNYVR
>233	>comp52_c1_seq1_5	KSHSISYLGLCK
>234	>comp52_c1_seq1_5	LADQCVFKHDCDECR
>235	>comp52_c1_seq1_5	LPPAGDMLEMEWDEELSKVAQK
>236	>comp52_c1_seq1_5	SHSISYLGLCK
>237	>comp52_c1_seq1_5	TVTLNGGETTTLK
>238	>comp52_c1_seq1_5	VGCGYVSFEK
>239	>comp52_c1_seq1_5	VLGERGPDMDEGDFSR
>240	>comp1072_c0_seq4_6	NFEDINQVQNALR
>241	>comp197_c0_seq1_4	DVIAPVASTVIQNVNR
>242	>comp197_c0_seq1_4	ILVNQEETFNGDK
>243	>comp197_c0_seq1_4	LSHPNTLIYPYINYILPGTKK
>244	>comp197_c0_seq1_4	TYVKDVIAPVASTVIQNVNR
>245	>comp197_c0_seq1_4	VAIEEWENSAK
>246	>comp197_c0_seq1_4	VAIEEWENSAKEWMLK
>247	>comp197_c0_seq1_4	YLIDPKNPTFK
>248	>comp431_c0_seq1_5	LFYLYGADPVIGISVAPNDKNTK
>249	>comp61_c0_seq1_2	FSDNTAGIAFPNTVCR

>250	>comp61_c0_seq1_2	GIAAEETLKNMAK
>251	>comp61_c0_seq1_2	SNEANLAPYIPSQPR
>252	>MMa41877	LVAISLEGNR
>253	>MMa23413	TKYTPQQIAEATVTALQR
>254	>MMa31039	IISNEELNVIAGDVSPGK
>255	>MMa31039	IISNEELNVIAGDVSPGKGK
>256	>MMa50337	FSDNTAGIAFPNTVCR
>257	>MMa50337	GIAAEETLKNMAK
>258	>MMa50337	NSGDSMEVYLANLMNAVK
>259	>MMa50337	SNEANLAPYIPSQPR
>260	>MMa50337	STDILVLR
>261	>MMa42691	AMEDGIVYSGK
>262	>MMa42691	AMEDGIVYSGKYK
>263	>MMa42691	APSTDDYLLYCQFSDKDSK
>264	>MMa42691	DASPQYFEFKEYGVR
>265	>MMa42691	FQHKNGICAYLIGR
>266	>MMa42691	IGCGVAGYTEANTK
>267	>MMa42691	NGICAYLIGR
>268	>MMa42691	TSSSWNTIGILMK
>269	>MMa42691	VIAGGSVSDSDKETILK
>270	>MMa42691	VLYTCNYGPAGNMIGSEAYQVGSPCSACPENT<br>K
>271	>MMa42689	FGPNDASEKPGSFVSFEFSAPGLIFPGGSK
>272	>MMa42689	FQHKNGICAYLIGR
>273	>MMa42689	NGICAYLIGR
>274	>MMa42689	VLYTCNYGPAGNIK
>275	>comp48_c0_seq1_5	IGNIDSDSGQEYTFQSFSK
>276	>comp48_c0_seq1_5	LSTPVTFTPHVNR
>277	>comp48_c0_seq1_5	NSGIDGVCEGDSGGPLVVR
>278	>comp48_c0_seq1_5	NSGIDGVCEGDSGGPLVVRR
>279	>comp48_c0_seq1_5	YANPGEFPWMVFIK
>280	>comp545_c0_seq1_1	AGVHLLGTNGAIPAHK
>281	>comp545_c0_seq1_1	DAPPLELPKGVGFK
>282	>comp545_c0_seq1_1	ESYLCTSFK
>283	>comp545_c0_seq1_1	FINGGTDNSGIVLTLLPGDDQSVTKR
>284	>comp545_c0_seq1_1	FNMLMPDVQPLQK
>285	>comp545_c0_seq1_1	GSGINYLVLQVHYADVTK
>286	>comp545_c0_seq1_1	HDPLEPQMFYPVENQDLTIEKGDILAAR
>287	>comp545_c0_seq1_1	IEQPVTLHPFAFR
>288	>comp1072_c0_seq3_6	LYLPLHDLPDTIFANQGNCLR
>289	>comp52_c1_seq1_5	EGVSKDEQQTILELHNK
>290	>comp52_c1_seq1_5	GANVAGSNSNNQFEVQLDR

>291	>comp52_c1_seq1_5	GAPAQYLDVNYVR
>292	>comp52_c1_seq1_5	SHSISYLGLCK
>293	>comp52_c1_seq1_5	TVTLNGGETTTLK
>294	>comp1072_c0_seq4_6	NFEDINQVQNALR
>295	>MMa15302	SYELPDGQVITIGNER
>296	>comp197_c0_seq1_4	LSHPNTLIYPYINYILPGTK
>297	>comp197_c0_seq1_4	TYVKDVIAPVASTVIQNVNR
>298	>comp197_c0_seq1_4	VAIEEWENSAKEWMLK
>299	>comp544_c0_seq1_5	GITEDLPINALVPLKNLR
>300	>comp544_c0_seq1_5	LTLVCEDFENINQVHSALR
>301	>comp61_c0_seq1_2	FSDNTAGIAFPNTVCR
>302	>comp61_c0_seq1_2	GIAAEETLKNMAK
>303	>comp61_c0_seq1_2	NSGDSMEVYLANLMNAVK
>304	>comp61_c0_seq1_2	SNEANLAPYIPSQPR
>305	>comp61_c0_seq1_2	STDILVLR
>306	>MMa28760	YSFTYALVRK
>307	>MMa33253	YDPSVQPQMLAR
>308	>MMa50337	GIAAEETLKNMAK
>309	>MMa50337	SNEANLAPYIPSQPR
>310	>MMa17864	SELWAYETNK
>311	>MMa08776	ISVSGLGPLNSTVK
>312	>MMa08776	ISVSGLGPLNSTVKK
>313	>MMa08776	QSNSFMDAVLQNVKTEGR
>314	>comp661_c0_seq1_2	FILVADDSQANER
>315	>comp1468_c0_seq1_6	ISVEEEINWPHK
>316	>MMa31039	FVLTAAHCVYDDER
>317	>MMa31039	FVLTAAHCVYDDERR
>318	>MMa31039	IISNEELNVIAGDVSPGKGK
>319	>MMa31039	KFVLTAAHCVYDDER
>320	>MMa31039	KIISNEELNVIAGDVSPGK
>321	>MMa31039	TKPAMFTDIR
>322	>MMa31039	YLPVSEIYTYNGYNGNWVNDIAILK
>323	>MMa08812	CADPGYPGVYTR
>324	>comp78389_c0_seq1_6	APLYLEIENK
>325	>comp52_c1_seq1_5	GANVAGSNSNNQFEVQLDR
>326	>comp52_c1_seq1_5	GAPAQYLDVNYVR
>327	>comp52_c1_seq1_5	KGANVAGSNSNNQFEVQLDR
>328	>comp52_c1_seq1_5	SHSISYLGLCK
>329	>comp52_c1_seq1_5	TVTLNGGETTTLK
>330	>comp20_c0_seq1_3	YAVPEGTLR
>331	>comp10941_c0_seq2_2	KYLQFYFR
>332	>comp61_c0_seq1_2	GIAAEETLKNMAK

>333	>comp61_c0_seq1_2	SNEANLAPYIPSQPR
>334	>comp16_c0_seq1_5	LHGVTYGYCYNSR
>335	>MMa02192	VSCLWGNEGCNKECR
>336	>comp20_c0_seq1_3	YAVPEGTLR
>337	>MMa13616	LPDSVPIRVPGK
>338	>MMa17865	KSELWNYNTNK
>339	>MMa17865	SELWNYNTNK
>340	>MMa17865	VWCVINNESCNSECK
>341	>MMa17864	IWCVINNESCNSECK
>342	>MMa17864	IWCVINNESCNSECKLR
>343	>MMa17864	SELWAYETNK
>344	>MMa17864	SELWAYETNKCNGR
>345	>MMa55372	YGNACWCYK
>346	>MMa55372	YGNACWCYKLPDDAR
>347	>MMa04555	VAECLFNNYCNNECTK
>348	>MMa04555	VYYADKGYCCLLK
>349	>comp7_c0_seq5_6	DAYIAKPHNCVYECAR
>350	>comp82847_c0_seq1_1	YGNFGINVSTK
>351	>comp20_c0_seq1_3	TIIQTAVHK
>352	>comp20_c0_seq1_3	TIIQTAVHKLGK
>353	>comp20_c0_seq1_3	TQFGCPAYQGYCDDHCQDIK
>354	>comp37980_c0_seq1_1	PTYSILNFITLK
>355	>comp21_c0_seq1_5	LHLASGGSCQQPAPFVK
>356	>comp53_c0_seq1_4	CNSWNDNLITK
>357	>comp8602_c1_seq2_6	IGVYLLNK
>358	>MMa34629	DGYIADDKNCAYFCGR
>359	>comp16_c0_seq1_5	LEDKDVTIWNAVK
>360	>comp16_c0_seq1_5	LHGVTYGYCYNSR
>361	>comp16_c0_seq1_5	YYCTILGENEYCRK
>362	>MMa56510	QILEPYFPHCCPSSPK
>363	>MMa02192	VSCLWGNEGCNK
>364	>MMa02192	VSCLWGNEGCNKECR
>365	>comp21_c0_seq1_5	LHLASGGSCQQPAPFVK
>366	>comp7_c0_seq5_6	DAYIAKPHNCVYECAR
>367	>comp16_c0_seq1_5	LEDKDVTIWNAVK
>368	>comp16_c0_seq1_5	LHGVTYGYCYNSR
>369	>comp16_c0_seq1_5	YYCTILGENEYCR
>370	>MMa02192	VSCLWGNEGCNK
>371	>MMa02192	VSCLWGNEGCNKECR
>372	>MMa20191	YGNACWCIDLPDKVPIR
>373	>MMa04555	VAECLFNNYCNNECTK
>374	>comp38_c0_seq1_5	VSECLLNNYCNNICTK

>375	>MMa17865	KSELWNYNTNK
>376	>MMa17865	VWCVINNESCNSECK
>377	>MMa17865	VWCVINNESCNSECKIR
>378	>MMa17864	GNYGYCYFWK
>379	>MMa17864	IWCVINNESCNSECK
>380	>MMa17864	IWCVINNESCNSECKLR
>381	>MMa17864	LACYCEGAPKSELWAYETNK
>382	>MMa17864	RGNYGYCYFWK
>383	>MMa17864	SELWAYETNKCNGR
>384	>MMa34629	DGYIADDKNCAYFCGR
>385	>MMa13616	LPDSVPIRVPGK
>386	>MMa55372	DGYIADDRNCPYFCGR
>387	>MMa55372	YGNACWCYKLPDDAR
>388	>MMa13619	YGNACWCIKLPDR
>389	>MMa13616	LCTENGAESGYCQWGGK
>390	>MMa13616	LPDSVPIR
>391	>MMa13616	LPDSVPIRVPGK
>392	>MMa17865	VWCVINNESCNSECK
>393	>MMa17865	VWCVINNESCNSECKIR
>394	>MMa17864	IWCVINNESCNSECK
>395	>MMa17864	IWCVINNESCNSECKLR
>396	>MMa17864	LACYCEGAPKSELWAYETNK
>397	>MMa17864	SELWAYETNK
>398	>MMa17864	SELWAYETNKCNGR
>399	>MMa55372	DGYIADDRNCPYFCGR
>400	>MMa55372	YGNACWCYK
>401	>MMa55372	YGNACWCYKLPDDAR
>402	>MMa04555	VYYADKGYCCLLK
>403	>comp7_c0_seq5_6	DAYIAKPHNCVYECAR
>404	>comp38_c0_seq1_5	VSECLLNNYCNNICTK
>405	>MMa35530	LTSMSEYACPVIEK
>406	>comp20_c0_seq1_3	YAVPEGTLR
>407	>comp16_c0_seq1_5	LEDKDVTIWNAVK
>408	>comp16_c0_seq1_5	LHGVTYGYCYNSR
>409	>comp16_c0_seq1_5	YYCTILGENEYCR
>410	>comp404_c0_seq1_2	NNFLNIENCCK
>411	>comp404_c0_seq1_2	TCESFIYGGVGGNK
>412	>MMa34629	DGYIADDKNCAYFCGR
>413	>comp34_c2_seq1_2	LACWCDDIHNWVPTWSR
>414	>MMa02192	VSCLWGNEGCNK
>415	>MMa02192	VSCLWGNEGCNKECR
>416	>MMa13616	LCTENGAESGYCQWGGK

>417	>MMa13616	LPDSVPIRVPGK
>418	>MMa17865	SELWNYNTNK
>419	>MMa17865	VWCVINNESCNSECK
>420	>MMa17864	IWCVINNESCNSECK
>421	>MMa17864	SELWAYETNKCNGR
>422	>MMa04118	CFASSECWTACK
>423	>MMa04118	CFASSECWTACKK
>424	>MMa55372	DGYIADDRNCPYFCGR
>425	>MMa55372	YGNACWCYKLPDDAR
>426	>comp7_c0_seq4_4	LCTDNGAESGYCQWGGK
>427	>comp7833_c0_seq7_2	RLTNNLDNEYFLDMDYVR
>428	>comp38_c0_seq1_5	VSECLLNNYCNNICTK
>429	>MMa35530	LTSMSEYACPVIEK
>430	>comp141_c0_seq1_4	KGFLLGFCDQR
>431	>MMa20191	YGNACWCIDLPDKVPIR
>432	>comp20_c0_seq1_3	YAVPEGTLR
>433	>MMa12627	DLPDNVPIRVPGK
>434	>comp7_c0_seq5_6	DAYIAKPHNCVYECAR
>435	>comp404_c0_seq1_2	TCESFIYGGVGGNK
>436	>MMa34629	DGYIADDKNCAYFCGR
>437	>comp16_c0_seq1_5	YYCTILGENEYCR
>438	>MMa13619	LCTDNGAESGYCQWGGR
>439	>MMa02192	VSCLWGNEGCNK
>440	>MMa02192	VSCLWGNEGCNKECR
>441	>MMa13616	LPDSVPIR
>442	>MMa13616	LPDSVPIRVPGK
>443	>comp968_c0_seq1_1	VTGNYDLYSR
>444	>comp20_c0_seq1_3	YAVPEGTLR
>445	>MMa12627	DAYIAQNYNCLYHCAR
>446	>MMa12627	DLPDNVPIR
>447	>MMa12627	DLPDNVPIRVPGK
>448	>MMa13619	LCTDNGAESGYCQWGGR
>449	>MMa04118	CFASSECWTACK
>450	>MMa04118	CFASSECWTACKK
>451	>comp58416_c0_seq1_5	SHPIGPHIWGSSVMK
>452	>comp20_c0_seq1_3	YAVPEGTLR
>453	>MMa12627	DAYIAQNYNCLYHCAR
>454	>MMa12627	DLPDNVPIR
>455	>MMa12627	DLPDNVPIRVPGK
>456	>comp88829_c0_seq1_4	MIMDVSTTFIPGKK
>457	>MMa00982	CFGPQCLCNR
>458	>comp605_c0_seq1_3	IGCAGIQYQGSR

>459	>comp605_c0_seq1_3	KDPYSEGEVIAVR
>460	>comp10743_c0_seq1_4	FLNIEEQQESCHFR
>461	>comp10743_c0_seq1_4	LIEIVGSFVCLNK
>462	>comp188_c0_seq1_3	CICSTLAEFLR
>463	>comp188_c0_seq1_3	CNELVDNLVWDNR
>464	>comp188_c0_seq1_3	CPEQSEPEFLPEK
>465	>comp188_c0_seq1_3	CTVDGWYCPLK
>466	>comp188_c0_seq1_3	DKTITSGVCATWGQFNYR
>467	>comp188_c0_seq1_3	EQTCIDWCRCPEGK
>468	>comp188_c0_seq1_3	ETILLTVEPFLR
>469	>comp188_c0_seq1_3	FAISWKDPTVECR
>470	>comp188_c0_seq1_3	GLCGILNGDYRDDLFSR
>471	>comp188_c0_seq1_3	GRVIPWTESIPLCAPECYLK
>472	>comp188_c0_seq1_3	GWEESVEVPR
>473	>comp188_c0_seq1_3	HEYCACETENEKCICSTLAEFLR
>474	>comp188_c0_seq1_3	IDIDLSHISSDIIQLK
>475	>comp188_c0_seq1_3	IDIDLSHISSDIIQLKDK
>476	>comp188_c0_seq1_3	IFNNPTFLNCK
>477	>comp188_c0_seq1_3	IFVVIDNK
>478	>comp188_c0_seq1_3	IFVVIDNKEFVVSK
>479	>comp188_c0_seq1_3	IYIDAMPTLYNQLK
>480	>comp188_c0_seq1_3	KIFVVIDNK
>481	>comp188_c0_seq1_3	KMDEFEEECNAESLSNHVCQYK
>482	>comp188_c0_seq1_3	LGGTVSGDWR
>483	>comp188_c0_seq1_3	LYDRVEMNGLECK
>484	>comp188_c0_seq1_3	MDEFEEECNAESLSNHVCQYK
>485	>comp188_c0_seq1_3	SAPMCHPFNSPIGTQILK
>486	>comp188_c0_seq1_3	SAPMCHPFNSPIGTQILKNDK
>487	>comp188_c0_seq1_3	SCPMTCQGSQYLCR
>488	>comp188_c0_seq1_3	SSGQCVLQEECNCTYFGEVYAAGSLRK
>489	>comp188_c0_seq1_3	SVEDFANSWK
>490	>comp188_c0_seq1_3	SVEDFANSWKK
>491	>comp188_c0_seq1_3	TITSGVCATWGQFNYR
>492	>comp188_c0_seq1_3	VDIPDDITVFWDR
>493	>comp188_c0_seq1_3	VDIPDDITVFWDRNNR
>494	>comp188_c0_seq1_3	VIPWTESIPLCAPECYLK
>495	>comp188_c0_seq1_3	VKCNELVDNLVWDNR
>496	>comp188_c0_seq1_3	WDGKETILLTVEPFLR
>497	>comp188_c0_seq1_3	YEFMGSCSYYLVFHK
>498	>comp188_c0_seq1_3	YSCASSAMESLVR
>499	>MMa02192	VSCLWGNEGCNKECR
>500	>MMa42689	LLAGGSVSDSDKQTILK

>501	>comp61_c0_seq1_2	FSDNTAGIAFPNTVCR
>502	>comp61_c0_seq1_2	SNEANLAPYIPSQPR
>503	>comp268_c0_seq2_2	AVVLTGFGGLK
>504	>comp268_c0_seq2_2	IIPNITLIGTASK
>505	>comp105006_c0_seq1_4	LATVISPR
>506	>comp188_c0_seq1_3	TITSGVCATWGQFNYR
>507	>comp214_c0_seq1_6	AGPSSLVAIR
>508	>comp214_c0_seq1_6	AISAIVNSNDDNGVLIGNWSGK
>509	>comp214_c0_seq1_6	ARNTESTFYAFGR
>510	>comp214_c0_seq1_6	DETADIGWKR
>511	>comp214_c0_seq1_6	DIDPKESFLHYER
>512	>comp214_c0_seq1_6	DLIKLEFTFGPSPNLSK
>513	>comp214_c0_seq1_6	DLQNITEEYKR
>514	>comp214_c0_seq1_6	ENVEFVLKPK
>515	>comp214_c0_seq1_6	ESFLHYER
>516	>comp214_c0_seq1_6	EYVLNDTGKIFVGSYSVPK
>517	>comp214_c0_seq1_6	GTLVQLILTNNK
>518	>comp214_c0_seq1_6	GTLVQLILTNNKEFTKPK
>519	>comp214_c0_seq1_6	IFVGSYSVPKGR
>520	>comp214_c0_seq1_6	ISFTNPLDK
>521	>comp214_c0_seq1_6	ISFTNPLDKK
>522	>comp214_c0_seq1_6	IYAIATVK
>523	>comp214_c0_seq1_6	IYAIATVKETK
>524	>comp214_c0_seq1_6	KLTDCTISIECPGVTAPYR
>525	>comp214_c0_seq1_6	LEFTFGPSPNLSK
>526	>comp214_c0_seq1_6	LKPQTEGNTKLVLVFNSR
>527	>comp214_c0_seq1_6	LSINKYQVGR
>528	>comp214_c0_seq1_6	LTDCTISIECPGVTAPYR
>529	>comp214_c0_seq1_6	LVLVFNSR
>530	>comp214_c0_seq1_6	NTESTFYAFGR
>531	>comp214_c0_seq1_6	NYPHTNSENYISYR
>532	>comp214_c0_seq1_6	RGSPFFVAIR
>533	>comp214_c0_seq1_6	STLPVSFLCK
>534	>comp214_c0_seq1_6	SVRYGQCWVFAGVTTTICR
>535	>comp214_c0_seq1_6	TIWAVLSASSVYYTGILAAK
>536	>comp214_c0_seq1_6	VIEVDSVELYSK
>537	>comp214_c0_seq1_6	VIEVDSVELYSKQNALEHK
>538	>comp214_c0_seq1_6	YGQCWVFAGVTTTICR
>539	>MMa27598	ADIITLDGGDVYR
>540	>MMa27598	ADLLFKDSTTELK
>541	>MMa27598	ASSLQQSPSIYLGDFR
>542	>MMa27598	CLIENNGDVAFVK

>543	>MMa27598	CNDLGKVVLSR
>544	>MMa27598	DALGDDYLNAVEAADPLLCTQEA
>545	>MMa27598	DPIVDAELKR
>546	>MMa27598	DSTTELKVLPLHATYR
>547	>MMa27598	EGRADIITLDGGDVYR
>548	>MMa27598	FCTTSEQGINKCNDLGK
>549	>MMa27598	HPSLCSLCGDPAK
>550	>MMa27598	HTTASEYTNGKSDLDWAK
>551	>MMa27598	KHPSLCSLCGDPAK
>552	>MMa27598	LCQQCIGDENGEHKCSR
>553	>MMa27598	MDTSIGCILESTTADCITAVK
>554	>MMa27598	MEEEVDLVQCISRPDR
>555	>MMa27598	QIFSRYPSNIPQIQR
>556	>MMa27598	RMDTSIGCILESTTADCITAVK
>557	>MMa27598	RNLGGGADLSCSWGVRPSNAFVVSSHK
>558	>MMa27598	SDIYSGYEGALGCLNDGK
>559	>MMa27598	SDIYSGYEGALGCLNDGKGDIAFSK
>560	>MMa27598	SDLDWAKELK
>561	>MMa27598	SLELSSLHELEGK
>562	>MMa27598	SLELSSLHELEGKR
>563	>MMa27598	SYGNKADLLFK
>564	>MMa27598	TAAVADLFSASCAPGANDAK
>565	>MMa27598	TQEEKEEIYSTLR
>566	>MMa27598	VPSHQVMISGSATFDR
>567	>MMa27598	VPSHQVMISGSATFDRR
>568	>MMa27598	YPSNIPQIQR
>569	>MMa27598	YSGYSGAFR
>570	>comp39704_c0_seq1_6	NTDTKLPMIFASPK
>571	>MMa15018	ADSNMQTLADLK
>572	>MMa15018	EGNADLITLSGR
>573	>MMa15018	LCIGDESGANVCSPTK
>574	>MMa52178	EAILDDAQYVPDPK
>575	>MMa52178	RNNIVLLANILSK
>576	>comp120_c0_seq1_2	CIAGPEINCDSEIEGK
>577	>comp120_c0_seq1_2	TWTAFCEEENMVPCK
>578	>comp48_c0_seq1_5	LSTPVTFTPHVNR
>579	>comp12251_c0_seq10_5	ITTSRNTPYNDLASGCGGCVSLNISK
>580	>comp976_c0_seq1_5	ELISNASDALDKIR
>581	>comp976_c0_seq1_5	FAFQAEVNR
>582	>comp976_c0_seq1_5	GVVDSDDLPLNVSR
>583	>comp976_c0_seq1_5	LGIIEDPGNR
>584	>comp214_c0_seq1_6	AGPSSLVAIR

>585	>comp214_c0_seq1_6	AISAIVNSNDDNGVLIGNWSGK
>586	>comp214_c0_seq1_6	DLQNITEEYKR
>587	>comp214_c0_seq1_6	GSPFFVAIR
>588	>comp214_c0_seq1_6	GTLVQLILTNNK
>589	>comp214_c0_seq1_6	GTLVQLILTNNKEFTKPK
>590	>comp214_c0_seq1_6	ISFTNPLDK
>591	>comp214_c0_seq1_6	ISFTNPLDKK
>592	>comp214_c0_seq1_6	KLTDCTISIECPGVTAPYR
>593	>comp214_c0_seq1_6	LTDCTISIECPGVTAPYR
>594	>comp214_c0_seq1_6	LVLVFNSR
>595	>comp214_c0_seq1_6	RGSPFFVAIR
>596	>comp214_c0_seq1_6	STLPVSFLCK
>597	>comp214_c0_seq1_6	VIEVDSVELYSK
>598	>comp214_c0_seq1_6	YGQCWVFAGVTTTICR
>599	>MMa53616	FGFGLLDAEAMVK
>600	>MMa53616	GALELYLISPSGSK
>601	>MMa53616	SIFITDSQTQLPQPIATGK
>602	>comp105006_c0_seq1_4	LATVISPR
>603	>comp774_c0_seq1_5	TLNDELTGLR
>604	>comp775_c0_seq1_1	ASSALWAVKLDNDLSGK
>605	>comp775_c0_seq1_1	QYFVSWKEPEEQIGLGR
>606	>comp775_c0_seq1_1	SVELDDVLGGSPVQHR
>607	>comp775_c0_seq1_1	TSENEVCLYR
>608	>comp775_c0_seq1_1	VVQGAEPQHFLR
>609	>comp775_c0_seq1_1	VYTSEQIAASITEPDFNVSALHR
>610	>MMa50472	EAIIDDAQYVPDPK
>611	>MMa50472	NLVAYDKVALVAGQHK
>612	>MMa50472	RNNIVLLANILSK
>613	>MMa02192	VSCLWGNEGCNKECR
>614	>comp61_c0_seq1_2	FSDNTAGIAFPNTVCR
>615	>comp61_c0_seq1_2	GIAAEETLKNMAK
>616	>comp1024_c0_seq1_4	IMPLIAVADPGYLIAR
>617	>comp1024_c0_seq1_4	LLETFLLDR
>618	>comp1024_c0_seq1_4	MQEEVKWMPLSGPVAGILPYEGK
>619	>comp1024_c0_seq1_4	MVETIDKLVGFLLEK
>620	>comp1024_c0_seq1_4	VLLISFDGFR
>621	>comp1024_c0_seq1_4	WMPLSGPVAGILPYEGK
>622	>MMa42691	IGCGVAGYTEANTK
>623	>MMa42691	TSSSWNTIGILMK
>624	>MMa42691	VIAGGSVSDSDKETILK
>625	>MMa42691	VLYTCNYGPAGNMIGSEAYQVGSPCSACPENT<br>K

>626	>comp41_c0_seq1_5	DANLSLMKDVNNLTYLIK
>627	>MMa48694	IWNSQLLQYAGYR
>628	>MMa48694	LTEVIADISER
>629	>comp545_c0_seq1_1	ESYLCTSFK
>630	>comp545_c0_seq1_1	IEQPVTLHPFAFR
>631	>comp1072_c0_seq1_4	ITELKDSLQELR
>632	>comp1072_c0_seq1_4	LGISSNLFNK
>633	>comp1072_c0_seq1_4	LGISSNLFNKLDVGK
>634	>comp1072_c0_seq1_4	LIELAEISR
>635	>comp1072_c0_seq1_4	MLTMIYNDLK
>636	>comp1072_c0_seq2_4	CPAYTGPCDCVFSR
>637	>comp52_c1_seq1_5	DEQQTILELHNK
>638	>comp52_c1_seq1_5	DGFCLTLSFRK
>639	>comp52_c1_seq1_5	EGVSKDEQQTILELHNK
>640	>comp52_c1_seq1_5	GANVAGSNSNNQFEVQLDR
>641	>comp52_c1_seq1_5	GAPAQYLDVNYVR
>642	>comp52_c1_seq1_5	KGANVAGSNSNNQFEVQLDR
>643	>comp52_c1_seq1_5	LADQCVFKHDCDECR
>644	>comp52_c1_seq1_5	SHSISYLGLCK
>645	>comp52_c1_seq1_5	TESSSDCQSQVEGANKWQTR
>646	>comp52_c1_seq1_5	TVTLNGGETTTLK
>647	>comp52_c1_seq1_5	VGCGYVSFEK
>648	>comp52_c1_seq1_5	VLGERGPDMDEGDFSR
>649	>comp52_c1_seq1_5	YLFNCDFKTESSSDCQSQVEGANK
>650	>comp1072_c0_seq3_6	LYLPLHDLPDTIFANQGNCLR
>651	>comp1072_c0_seq4_6	NFEDINQVQNALR
>652	>comp1172_c0_seq1_1	CPANTGPCDCFHVSSR
>653	>comp1172_c0_seq1_1	ELSLAYNPLK
>654	>comp1172_c0_seq1_1	IPINALISLER
>655	>comp1172_c0_seq1_1	LCLYDLTIISGK
>656	>comp1172_c0_seq1_1	LDLSYNSFTK
>657	>comp1172_c0_seq1_1	MNLNLPLLNIPDSLFSNQK
>658	>comp1172_c0_seq1_1	MYYKDFEDLSNLK
>659	>comp1172_c0_seq1_1	NELSSIAYGLEDLNNLEK
>660	>comp1172_c0_seq1_1	NIIQVQQALR
>661	>comp1172_c0_seq1_1	NQIDSIDDSLYGLTK
>662	>comp1172_c0_seq1_1	NVENIIIGEDNYVINK
>663	>comp105006_c0_seq1_4	LATVISPR
>664	>comp197_c0_seq1_4	DVIAPVASTVIQNVNR
>665	>comp197_c0_seq1_4	ILVNQEETFNGDK
>666	>comp197_c0_seq1_4	LSHPNTLIYPYINYILPGTK
>667	>comp197_c0_seq1_4	TYVKDVIAPVASTVIQNVNR

>668	>comp197_c0_seq1_4	VAIEEWENSAK
>669	>comp3601_c0_seq1_6	ATNVHLPNDASFVIANSCVEMNK
>670	>comp431_c0_seq1_5	DISNYLSIQFIR
>671	>comp431_c0_seq1_5	DISNYLSIQFIRK
>672	>comp431_c0_seq1_5	EFAEAFNCVK
>673	>comp431_c0_seq1_5	IENYYAELEFNSADSALTNYVK
>674	>comp431_c0_seq1_5	ILITYPETIAK
>675	>comp431_c0_seq1_5	NKIENYYAELEFNSADSALTNYVK
>676	>comp431_c0_seq1_5	NSFGNLPDHEMWTTTAIR
>677	>comp431_c0_seq1_5	QLNLPDNAR
>678	>comp431_c0_seq1_5	QNNEKDISNYLSIQFIR
>679	>comp431_c0_seq1_5	TLTEENDRNSWPK
>680	>comp431_c0_seq1_5	VIGALANSKEFAEAFNCVK
>681	>comp431_c0_seq1_5	VRNSFGNLPDHEMWTTTAIR
>682	>comp431_c0_seq1_5	YRQLNLPDNAR
>683	>MMa10526	FANICQENVPVKR
>684	>MMa02192	VSCLWGNEGCNKECR
>685	>comp431_c0_seq1_5	YRQLNLPDNAR
>686	>comp52_c1_seq1_5	LADQCVFKHDCDECR
>687	>comp4077_c0_seq1_2	NNQIETLDNSLYNLQELKR
>688	>MMa35583	LGISSDLFNKLDVGK
>689	>MMa35583	LNSLGNSLSNLEMLK
>690	>MMa42691	IGCGVAGYTEANTK
>691	>comp431_c0_seq1_5	VIGALANSKEFAEAFNCVK
>692	>comp431_c0_seq1_5	YRQLNLPDNAR
>693	>comp52_c1_seq1_5	TESSSDCQSQVEGANKWQTR
>694	>comp6409_c0_seq1_2	SSTFGPSK
>695	>comp4077_c0_seq1_2	LTTLDISNNR
>696	>comp268_c0_seq2_2	HLLYQQGGHEYIR
>697	>MMa47879	IGVIKAEAQNIAR
>698	>comp431_c0_seq1_5	QLNLPDNAR
>699	>comp431_c0_seq1_5	YRQLNLPDNAR
>700	>comp105006_c0_seq1_4	LATVISPR
>701	>comp52_c1_seq1_5	LADQCVFKHDCDECR
>702	>comp431_c0_seq1_5	ILITYPETIAK
>703	>comp431_c0_seq1_5	QLNLPDNAR
>704	>comp431_c0_seq1_5	TLTEENDRNSWPK
>705	>comp431_c0_seq1_5	YRQLNLPDNAR
>706	>comp1072_c0_seq1_4	LIELAEISR
>707	>comp1072_c0_seq1_4	TLTNELTGLK
>708	>comp1201_c0_seq2_1	YLFNFQMK
>709	>comp1172_c0_seq1_1	CPANTGPCDCFHVSSR

>710	>comp1172_c0_seq1_1	ELSLAYNPLK
>711	>comp1172_c0_seq1_1	IPINALISLER
>712	>comp1172_c0_seq1_1	NIIQVQQALR
>713	>MMa48694	LTEVIADISER
>714	>MMa35583	LIELAEISR
>715	>MMa42691	IGCGVAGYTEANTK
>716	>comp105006_c0_seq1_4	LATVISPR
>717	>comp197_c0_seq1_4	TVPSMDFKR
>718	>comp197_c0_seq1_4	VAIEEWENSAK
>719	>comp52_c1_seq1_5	GAPAQYLDVNYVR
>720	>comp52_c1_seq1_5	SHSISYLGLCK
>721	>comp52_c1_seq1_5	TESSSDCQSQVEGANKWQTR
>722	>comp52_c1_seq1_5	TVTLNGGETTTLK
>723	>comp52_c1_seq1_5	VGCGYVSFEK
>724	>comp52_c1_seq1_5	VGCGYVSFEKR
>725	>comp31795_c0_seq1_5	NIIQVKQIVK
>726	>MMa42689	NGICAYLIGR
>727	>MMa42689	YTEAYQVGSPCSACPENTK
>728	>MMa35583	LIELAEISR
>729	>comp1072_c0_seq1_4	AVISLKFLYNLK
>730	>comp1072_c0_seq1_4	ITELKDSLQELR
>731	>comp1072_c0_seq1_4	LIELAEISR
>732	>comp1072_c0_seq1_4	TLTNELTGLK
>733	>comp32566_c0_seq1_5	EASVLSVK
>734	>comp545_c0_seq1_1	AGVHLLGTNGAIPAHK
>735	>comp166733_c0_seq1_5	EIISIILSR
>736	>MMa17865	VWCVINNESCNSECK
>737	>MMa42691	AMEDGIVYSGK
>738	>MMa42691	APSTDDYLLYCQFSDKDSK
>739	>MMa42691	DASPQYFEFKEYGVR
>740	>MMa42691	FGPNDASEKPGSFVSFEFSAPGLIFPK
>741	>MMa42691	IGCGVAGYTEANTK
>742	>MMa42691	IGCGVAGYTEANTKK
>743	>MMa42691	NGICAYLIGR
>744	>MMa42691	TSSSWNTIGILMK
>745	>MMa42691	VIAGGSVSDSDKETILK
>746	>MMa42691	VLYTCNYGPAGNMIGSEAYQVGSPCSACPENT<br>K
>747	>comp105006_c0_seq1_4	LATVISPR
>748	>comp1072_c0_seq3_6	LYLPLHDLPDTIFANQGNCLR
>749	>comp197_c0_seq1_4	AKHPDWSPAQIEK
>750	>comp197_c0_seq1_4	DQPSEYFCK

>751	>comp197_c0_seq1_4	DVIAPVASTVIQNVNR
>752	>comp197_c0_seq1_4	GRGNCVWPEEPYTSWK
>753	>comp197_c0_seq1_4	HPDWSPAQIEK
>754	>comp197_c0_seq1_4	ILVNQEETFNGDK
>755	>comp197_c0_seq1_4	LSHPNTLIYPYINYILPGTK
>756	>comp197_c0_seq1_4	LSHPNTLIYPYINYILPGTKK
>757	>comp197_c0_seq1_4	NDIQEANDKLSWLWK
>758	>comp197_c0_seq1_4	QSTALCPSIYMQESHITK
>759	>comp197_c0_seq1_4	TVPSMDFK
>760	>comp197_c0_seq1_4	TVPSMDFKR
>761	>comp197_c0_seq1_4	TYVKDVIAPVASTVIQNVNR
>762	>comp197_c0_seq1_4	VAIEEWENSAK
>763	>comp197_c0_seq1_4	VAIEEWENSAKEWMLK
>764	>comp197_c0_seq1_4	VVWEVPSIMCSK
>765	>comp197_c0_seq1_4	YLIDPKNPTFK
>766	>comp197_c0_seq1_4	YPHIESHGDINGGMLQVSDLANHLK
>767	>comp1072_c0_seq2_4	CPAYTGPCDCVFSR
>768	>comp545_c0_seq1_1	AGVHLLGTNGAIPAHK
>769	>comp545_c0_seq1_1	ESYLCTSFK
>770	>comp1072_c0_seq1_4	ITELKDSLQELR
>771	>comp1072_c0_seq1_4	LIELAEISR
>772	>comp1072_c0_seq1_4	TLTNELTGLK
>773	>comp197_c0_seq1_4	ILVNQEETFNGDK
>774	>comp197_c0_seq1_4	VAIEEWENSAK
>775	>comp197_c0_seq1_4	YLIDPKNPTFK
>776	>MMa42689	CSEEYPGLCK
>777	>comp52_c1_seq1_5	SHSISYLGLCK
>778	>comp52_c1_seq1_5	TESSSDCQSQVEGANKWQTR
>779	>comp52_c1_seq1_5	TVTLNGGETTTLK
>780	>comp52_c1_seq1_5	VGCGYVSFEK
>781	>MMa42691	AMEDGIVYSGK
>782	>MMa42691	CSEEYPGLCK
>783	>MMa42691	EMEVTYTFEVKK
>784	>MMa42691	IGCGVAGYTEANTK
>785	>MMa42691	KDASPQYFEFK
>786	>MMa42691	SDREMEVTYTFEVK
>787	>MMa42691	VIAGGSVSDSDKETILK
>788	>comp1072_c0_seq1_4	ITELKDSLQELR
>789	>comp52_c1_seq1_5	DEQQTILELHNK
>790	>comp52_c1_seq1_5	EGVSKDEQQTILELHNK
>791	>comp52_c1_seq1_5	GANVAGSNSNNQFEVQLDR
>792	>comp52_c1_seq1_5	GAPAQYLDVNYVR

>793	>comp52_c1_seq1_5	KGANVAGSNSNNQFEVQLDR
>794	>comp52_c1_seq1_5	LADQCVFKHDCDECR
>795	>comp52_c1_seq1_5	SHSISYLGLCK
>796	>comp52_c1_seq1_5	TESSSDCQSQVEGANKWQTR
>797	>comp52_c1_seq1_5	TVTLNGGETTTLK
>798	>comp52_c1_seq1_5	VGCGYVSFEK
>799	>comp52_c1_seq1_5	VGCGYVSFEKR
>800	>comp52_c1_seq1_5	VLGERGPDMDEGDFSR
>801	>comp52_c1_seq1_5	YLFNCDFK
>802	>MMa42691	APSTDDYLLYCQFSDKDSK
>803	>MMa42691	DASPQYFEFKEYGVR
>804	>MMa42691	IGCGVAGYTEANTK
>805	>MMa42691	KDASPQYFEFK
>806	>MMa42691	NGICAYLIGR
>807	>MMa42691	SDREMEVTYTFEVK
>808	>MMa42691	TSSSWNTIGILMK
>809	>MMa42691	VIAGGSVSDSDKETILK
>810	>MMa42691	VLYTCNYGPAGNMIGSEAYQVGSPCSACPENT K
>811	>comp545_c0_seq1_1	AGVHLLGTNGAIPAHK
>812	>comp1072_c0_seq1_4	ITELKDSLQELR
>813	>comp1072_c0_seq1_4	LGISSNLFNK
>814	>comp1072_c0_seq1_4	LIELAEISR
>815	>comp1072_c0_seq1_4	MLTMIYNDLK
>816	>comp1072_c0_seq1_4	TLTNELTGLK
>817	>MMa31039	IISNEELNVIAGDVSPGK
>818	>comp545_c0_seq1_1	AGVHLLGTNGAIPAHK
>819	>comp545_c0_seq1_1	ESYLCTSFK
>820	>comp545_c0_seq1_1	IEQPVTLHPFAFR
>821	>comp52_c1_seq1_5	DEQQTILELHNK
>822	>comp52_c1_seq1_5	DGFCLTLSFRK
>823	>comp52_c1_seq1_5	EGVSKDEQQTILELHNK
>824	>comp52_c1_seq1_5	ESWTQLYVCNYGPAGNVEDSEMYR
>825	>comp52_c1_seq1_5	GANVAGSNSNNQFEVQLDR
>826	>comp52_c1_seq1_5	GAPAQYLDVNYVR
>827	>comp52_c1_seq1_5	KGANVAGSNSNNQFEVQLDR
>828	>comp52_c1_seq1_5	LADQCVFKHDCDECR
>829	>comp52_c1_seq1_5	LDSEGNEWLPYSIGIPMK
>830	>comp52_c1_seq1_5	LDSEGNEWLPYSIGIPMKQPMQISLK
>831	>comp52_c1_seq1_5	LPPAGDMLEMEWDEELSK
>832	>comp52_c1_seq1_5	SHSISYLGLCK
>833	>comp52_c1_seq1_5	TESSSDCQSQVEGANKWQTR

>834	>comp52_c1_seq1_5	TVTLNGGETTTLK
>835	>comp52_c1_seq1_5	VGCGYVSFEK
>836	>comp52_c1_seq1_5	VGCGYVSFEKR
>837	>comp52_c1_seq1_5	VLGERGPDMDEGDFSR
>838	>comp1072_c0_seq4_6	NFEDINQVQNALR
>839	>comp197_c0_seq1_4	DVIAPVASTVIQNVNR
>840	>comp197_c0_seq1_4	TYVKDVIAPVASTVIQNVNR
>841	>MMa42691	APSTDDYLLYCQFSDK
>842	>MMa42691	APSTDDYLLYCQFSDKDSK
>843	>MMa42691	DASPQYFEFK
>844	>MMa42691	DASPQYFEFKEYGVR
>845	>MMa42691	EMEVTYTFEVKK
>846	>MMa42691	IGCGVAGYTEANTK
>847	>MMa42691	KDASPQYFEFK
>848	>MMa42691	NGICAYLIGR
>849	>MMa42691	SDREMEVTYTFEVK
>850	>MMa42691	TSSSWNTIGILMK
>851	>MMa42691	VIAGGSVSDSDKETILK
>852	>comp1072_c0_seq1_4	ITELKDSLQELR
>853	>comp1072_c0_seq1_4	LIELAEISR
>854	>comp1072_c0_seq1_4	TLTNELTGLK
>855	>comp61_c0_seq1_2	SNEANLAPYIPSQPR
>856	>comp545_c0_seq1_1	ESYLCTSFK
>857	>MMa35583	LGISSDLFNK
>858	>MMa35583	LIELAEISR
>859	>MMa35583	LNSLGNSLSNLEMLK
>860	>comp1072_c0_seq4_6	NFEDINQVQNALR
>861	>comp48_c0_seq1_5	NSGIDGVCEGDSGGPLVVR
>862	>comp4077_c0_seq1_2	TLTNELTDLK
>863	>comp105006_c0_seq1_4	LATVISPR
>864	>MMa41877	LVAISLEGNR
>865	>comp197_c0_seq1_4	DVIAPVASTVIQNVNR
>866	>comp197_c0_seq1_4	TYVKDVIAPVASTVIQNVNR
>867	>comp197_c0_seq1_4	VAIEEWENSAK
>868	>comp52_c1_seq1_5	DEQQTILELHNK
>869	>comp52_c1_seq1_5	EGVSKDEQQTILELHNK
>870	>comp52_c1_seq1_5	ESWTQLYVCNYGPAGNVEDSEMYR
>871	>comp52_c1_seq1_5	GANVAGSNSNNQFEVQLDR
>872	>comp52_c1_seq1_5	GAPAQYLDVNYVR
>873	>comp52_c1_seq1_5	GPDMDEGDFSR
>874	>comp52_c1_seq1_5	KGANVAGSNSNNQFEVQLDR
>875	>comp52_c1_seq1_5	KSHSISYLGLCK

>876	>comp52_c1_seq1_5	LADQCVFKHDCDECR
>877	>comp52_c1_seq1_5	LDSEGNEWLPYSIGIPMK
>878	>comp52_c1_seq1_5	LPPAGDMLEMEWDEELSK
>879	>comp52_c1_seq1_5	SHSISYLGLCK
>880	>comp52_c1_seq1_5	TESSSDCQSQVEGANKWQTR
>881	>comp52_c1_seq1_5	TVTLNGGETTTLK
>882	>comp52_c1_seq1_5	VGCGYVSFEK
>883	>comp52_c1_seq1_5	VGCGYVSFEKR
>884	>comp52_c1_seq1_5	VLGERGPDMDEGDFSR
>885	>comp52_c1_seq1_5	YLFNCDFKTESSSDCQSQVEGANK
>886	>comp166757_c0_seq1_1	KGHSTEILAMECYK
>887	>MMa42691	AMEDGIVYSGK
>888	>MMa42691	APSTDDYLLYCQFSDK
>889	>MMa42691	APSTDDYLLYCQFSDKDSK
>890	>MMa42691	DASPQYFEFKEYGVR
>891	>MMa42691	IGCGVAGYTEANTK
>892	>MMa42691	KDASPQYFEFK
>893	>MMa42691	KVLYTCNYGPAGNMIGSEAYQVGSPCSACPEN TK
>894	>MMa42691	TSSSWNTIGILMK
>895	>MMa42691	VIAGGSVSDSDKETILK
>896	>MMa42691	VLYTCNYGPAGNMIGSEAYQVGSPCSACPENT K
>897	>comp38_c0_seq1_5	VSECLLNNYCNNICTK
>898	>MMa05656	ILGLAYPGTLCNK
>899	>MMa17864	IWCVINNESCNSECK
>900	>MMa42689	FQHKNGICAYLIGR
>901	>MMa42689	NGICAYLIGR
>902	>MMa16313	QTLSENIADNGGLR
>903	>comp48_c0_seq1_5	IGNIDSDSGQEYTFQSFSK
>904	>comp545_c0_seq1_1	AGVHLLGTNGAIPAHK
>905	>comp545_c0_seq1_1	ESYLCTSFK
>906	>comp545_c0_seq1_1	FNMLMPDVQPLQK
>907	>comp545_c0_seq1_1	GSGINYLVLQVHYADVTK
>908	>comp545_c0_seq1_1	IEQPVTLHPFAFR
>909	>comp1072_c0_seq1_4	ITELKDSLQELR
>910	>comp1072_c0_seq1_4	LGISSNLFNK
>911	>comp1072_c0_seq1_4	LGISSNLFNKLDVGK
>912	>comp1072_c0_seq1_4	LIELAEISR
>913	>comp1072_c0_seq1_4	TLTNELTGLK
>914	>comp1072_c0_seq2_4	CPAYTGPCDCVFSR
>915	>comp52_c1_seq1_5	DEQQTILELHNK

>916	>comp52_c1_seq1_5	EGVSKDEQQTILELHNK
>917	>comp52_c1_seq1_5	GANVAGSNSNNQFEVQLDR
>918	>comp52_c1_seq1_5	GAPAQYLDVNYVR
>919	>comp52_c1_seq1_5	GPDMDEGDFSR
>920	>comp52_c1_seq1_5	KGANVAGSNSNNQFEVQLDR
>921	>comp52_c1_seq1_5	LADQCVFKHDCDECR
>922	>comp52_c1_seq1_5	SHSISYLGLCK
>923	>comp52_c1_seq1_5	TESSSDCQSQVEGANKWQTR
>924	>comp52_c1_seq1_5	TVTLNGGETTTLK
>925	>comp52_c1_seq1_5	VGCGYVSFEK
>926	>comp52_c1_seq1_5	VGCGYVSFEKR
>927	>comp52_c1_seq1_5	VLGERGPDMDEGDFSR
>928	>comp1072_c0_seq3_6	LYLPLHDLPDTIFANQGNCLR
>929	>MMa34459	LPPVVFVLGGR
>930	>MMa34459	QQTFAEAVTEPGLTFVAAK
>931	>MMa34459	YYTEFDYDNAR
>932	>MMa31039	IISNEELNVIAGDVSPGK
>933	>comp1072_c0_seq4_6	NFEDINQVQNALR
>934	>comp347_c1_seq1_6	ILGLAYPGTLCNK
>935	>comp197_c0_seq1_4	DVIAPVASTVIQNVNR
>936	>comp197_c0_seq1_4	GNCVWPEEPYTSWK
>937	>comp197_c0_seq1_4	IVIFYESQLGK
>938	>comp197_c0_seq1_4	QSTALCPSIYMQESHITK
>939	>comp197_c0_seq1_4	TYVKDVIAPVASTVIQNVNR
>940	>comp197_c0_seq1_4	VAIEEWENSAK
>941	>comp197_c0_seq1_4	VAIEEWENSAKEWMLK
>942	>MMa42691	AMEDGIVYSGK
>943	>MMa42691	AMEDGIVYSGKYK
>944	>MMa42691	APSTDDYLLYCQFSDK
>945	>MMa42691	APSTDDYLLYCQFSDKDSK
>946	>MMa42691	DASPQYFEFK
>947	>MMa42691	DASPQYFEFKEYGVR
>948	>MMa42691	EMEVTYTFEVKK
>949	>MMa42691	FQHKNGICAYLIGR
>950	>MMa42691	IGCGVAGYTEANTK
>951	>MMa42691	KDASPQYFEFK
>952	>MMa42691	NGICAYLIGR
>953	>MMa42691	SDREMEVTYTFEVK
>954	>MMa42691	TSSSWNTIGILMK
>955	>MMa42691	VIAGGSVSDSDKETILK
>956	>comp1072_c0_seq1_4	ITELKDSLQELR
>957	>comp1072_c0_seq1_4	LIELAEISR

>958	>comp1072_c0_seq1_4	TLTNELTGLK
>959	>comp4077_c0_seq1_2	LTTLDISNNR
>960	>comp4077_c0_seq1_2	TLTNELTDLKNLK
>961	>comp61_c0_seq1_2	ADIVMLITK
>962	>comp61_c0_seq1_2	DCPASDGYIMGDR
>963	>comp61_c0_seq1_2	FSDNTAGIAFPNTVCR
>964	>comp61_c0_seq1_2	GIAAEETLKNMAK
>965	>comp61_c0_seq1_2	MLFNTLDLR
>966	>comp61_c0_seq1_2	SNEANLAPYIPSQPR
>967	>MMa42689	APTSDDYLLYCHFSDK
>968	>MMa42689	APTSDDYLLYCHFSDKDSK
>969	>MMa42689	GWYTTEVK
>970	>MMa42689	LLAGGSVSDSDKQTILK
>971	>MMa42689	NGICAYLIGR
>972	>MMa42689	VLYTCNYGPAGNIK
>973	>MMa42689	YTEAYQVGSPCSACPENTK
>974	>MMa42691	AMEDGIVYSGK
>975	>MMa42691	DASPQYFEFKEYGVR
>976	>MMa42691	IGCGVAGYTEANTK
>977	>MMa42691	NGICAYLIGR
>978	>MMa42691	TSSSWNTIGILMK
>979	>MMa42691	VIAGGSVSDSDKETILK
>980	>comp431_c0_seq1_5	LFPESNLCMTK
>981	>comp105006_c0_seq1_4	LATVISPR
>982	>comp1072_c0_seq4_6	NFEDINQVQNALR
>983	>comp197_c0_seq1_4	DVIAPVASTVIQNVNR
>984	>comp197_c0_seq1_4	TVPSMDFKR
>985	>comp52_c1_seq1_5	GANVAGSNSNNQFEVQLDR
>986	>comp52_c1_seq1_5	GAPAQYLDVNYVR
>987	>comp52_c1_seq1_5	LADQCVFKHDCDECR
>988	>comp52_c1_seq1_5	SHSISYLGLCK
>989	>comp52_c1_seq1_5	TESSSDCQSQVEGANKWQTR
>990	>comp52_c1_seq1_5	TVTLNGGETTTLK
>991	>comp52_c1_seq1_5	VGCGYVSFEK
>992	>comp52_c1_seq1_5	YLFNCDFK
>993	>comp52_c1_seq1_5	YLFNCDFKTESSSDCQSQVEGANK
>994	>MMa42689	APTSDDYLLYCHFSDK
>995	>MMa42689	APTSDDYLLYCHFSDKDSK
>996	>MMa42689	ETQYQTLPSAANMMQLEWDDELAAIAQAHSN QCK
>997	>MMa42689	FGPNDASEKPGSFVSFEFSAPGLIFPGGSK
>998	>MMa42689	IGCGLTGFMDDNTNKVLYTCNYGPAGNIK

>999	>MMa42689	LATGKETQYQTLPSAANMMQLEWDDELAAIA QAHSNQCK
>1000	>MMa42689	LLAGGSVSDSDKQTILK
>1001	>MMa42689	NGICAYLIGR
>1002	>MMa42689	TSIISSSWHTIGILMK
>1003	>MMa42689	VLYTCNYGPAGNIK
>1004	>MMa42689	YTEAYQVGSPCSACPENTK
>1005	>comp1072_c0_seq1_4	LIELAEISR
>1006	>comp1072_c0_seq3_6	LYLPLHDLPDTIFANQGNCLR
>1007	>comp61_c0_seq1_2	FSDNTAGIAFPNTVCR
>1008	>comp61_c0_seq1_2	MLFNTLDLR
>1009	>comp61_c0_seq1_2	SNEANLAPYIPSQPR
>1010	>comp31348_c0_seq1_5	LLHNNIYKR
>1011	>comp197_c0_seq1_4	DVIAPVASTVIQNVNR
>1012	>comp197_c0_seq1_4	GNCVWPEEPYTSWK
>1013	>comp197_c0_seq1_4	LSHPNTLIYPYINYILPGTK
>1014	>comp197_c0_seq1_4	TYVKDVIAPVASTVIQNVNR
>1015	>comp52_c1_seq1_5	GAPAQYLDVNYVR
>1016	>comp52_c1_seq1_5	VGCGYVSFEK
>1017	>MMa42691	DASPQYFEFKEYGVR
>1018	>MMa42691	IGCGVAGYTEANTK
>1019	>MMa42691	NGICAYLIGR
>1020	>MMa42691	TSSSWNTIGILMK
>1021	>MMa42691	VIAGGSVSDSDKETILK
>1022	>MMa42689	APTSDDYLLYCHFSDK
>1023	>MMa42689	APTSDDYLLYCHFSDKDSK
>1024	>MMa42689	FGPNDASEKPGSFVSFEFSAPGLIFPGGSK
>1025	>MMa42689	IGCGLTGFMDDNTNKVLYTCNYGPAGNIK
>1026	>MMa42689	LLAGGSVSDSDKQTILK
>1027	>MMa42689	NGICAYLIGR
>1028	>MMa42689	TSIISSSWHTIGILMK
>1029	>MMa42689	VLYTCNYGPAGNIK
>1030	>MMa42689	YTEAYQVGSPCSACPENTK
>1031	>comp1072_c0_seq1_4	LIELAEISR
>1032	>comp61_c0_seq1_2	SNEANLAPYIPSQPR
>1033	>MMa35583	LIELAEISR
>1034	>MMa42691	AMEDGIVYSGK
>1035	>MMa42691	APSTDDYLLYCQFSDKDSK
>1036	>MMa42691	DASPQYFEFKEYGVR
>1037	>MMa42691	IGCGVAGYTEANTK
>1038	>MMa42691	NGICAYLIGR
>1039	>MMa42691	TSSSWNTIGILMK

>1040	>MMa42691	VIAGGSVSDSDKETILK
>1041	>comp105006_c0_seq1_4	LATVISPR
>1042	>comp1072_c0_seq4_6	NFEDINQVQNALR
>1043	>comp52_c1_seq1_5	GANVAGSNSNNQFEVQLDR
>1044	>comp52_c1_seq1_5	GAPAQYLDVNYVR
>1045	>comp52_c1_seq1_5	LADQCVFKHDCDECR
>1046	>comp52_c1_seq1_5	SHSISYLGLCK
>1047	>comp52_c1_seq1_5	TESSSDCQSQVEGANKWQTR
>1048	>comp52_c1_seq1_5	TVTLNGGETTTLK
>1049	>comp52_c1_seq1_5	VGCGYVSFEK
>1050	>MMa42689	LLAGGSVSDSDKQTILK
>1051	>MMa42689	VLYTCNYGPAGNIK
>1052	>MMa42689	YTEAYQVGSPCSACPENTK
>1053	>comp268_c0_seq2_2	AVVLTGFGGLK
>1054	>comp268_c0_seq2_2	AWAELTAVPAK
>1055	>comp268_c0_seq2_2	GYGLLKPLGR
>1056	>comp268_c0_seq2_2	HLLYQQGGHEYIR
>1057	>comp268_c0_seq2_2	IIPNITLIGTASK
>1058	>comp268_c0_seq2_2	NTILLHSAGGGVGQAVAQMAK
>1059	>comp268_c0_seq2_2	TVIDSTWAFEDVPEAMQR
>1060	>comp268_c0_seq2_2	YILYGTASIVSGETK
>1061	>comp169119_c0_seq1_5	AVTSLVPR
>1062	>comp41_c0_seq1_5	DANLSLMK
>1063	>comp60456_c0_seq1_2	ASMAVQITK
>1064	>MMa42689	GWYTTEVK
>1065	>MMa42689	LLAGGSVSDSDKQTILK
>1066	>MMa42689	VLYTCNYGPAGNIK
>1067	>MMa42689	YTEAYQVGSPCSACPENTK
>1068	>MMa52966	MPLTIGETADK
>1069	>comp1072_c0_seq1_4	LIELAEISR
>1070	>MMa35583	LIELAEISR
>1071	>comp105006_c0_seq1_4	LATVISPR
>1072	>comp268_c0_seq2_2	AVVLTGFGGLK
>1073	>comp268_c0_seq2_2	AWAELTAVPAK
>1074	>comp268_c0_seq2_2	HLLYQQGGHEYIR
>1075	>comp268_c0_seq2_2	IIPNITLIGTASK
>1076	>comp268_c0_seq2_2	ITLDPNMEPKPKPQPTK
>1077	>comp268_c0_seq2_2	NTILLHSAGGGVGQAVAQMAK
>1078	>comp268_c0_seq2_2	SGGLNFLDVMVR
>1079	>comp268_c0_seq2_2	SWWQVDKVNPIK
>1080	>comp268_c0_seq2_2	TVIDSTWAFEDVPEAMQR
>1081	>comp268_c0_seq2_2	VKSGGLNFLDVMVR

>1082	>comp268_c0_seq2_2	YILYGTASIVSGETK
>1083	>comp268_c0_seq2_2	AVVLTGFGGLK
>1084	>comp268_c0_seq2_2	AVVLTGFGGLKSVK
>1085	>comp268_c0_seq2_2	AWAELTAVPAK
>1086	>comp268_c0_seq2_2	EKNTILLHSAGGGVGQAVAQMAK
>1087	>comp268_c0_seq2_2	GYGLLKPLGR
>1088	>comp268_c0_seq2_2	HLLYQQGGHEYIR
>1089	>comp268_c0_seq2_2	IIPNITLIGTASK
>1090	>comp268_c0_seq2_2	IKTVIDSTWAFEDVPEAMQR
>1091	>comp268_c0_seq2_2	ITLDPNMEPKPKPQPTK
>1092	>comp268_c0_seq2_2	LFDENKSISGFNLR
>1093	>comp268_c0_seq2_2	NTILLHSAGGGVGQAVAQMAK
>1094	>comp268_c0_seq2_2	SGGLNFLDVMVR
>1095	>comp268_c0_seq2_2	SWWQVDKVNPIK
>1096	>comp268_c0_seq2_2	TVIDSTWAFEDVPEAMQR
>1097	>comp268_c0_seq2_2	VKSGGLNFLDVMVR
>1098	>comp268_c0_seq2_2	VNPIKLFDENK
>1099	>comp268_c0_seq2_2	YILYGTASIVSGETK
>1100	>comp508_c0_seq1_4	ILTSPPKTDALLYEGAICPEAK
>1101	>comp544_c0_seq1_5	SLNVADNQLVEIGEK
>1102	>comp1166_c0_seq1_4	GSFSYDGINEFLR
>1103	>comp1166_c0_seq1_4	GVVNVGAVDADKHQSLGGQYGVR
>1104	>comp1072_c0_seq1_4	ITELKDSLQELR
>1105	>comp4077_c0_seq1_2	TLTNELTDLKNLK
>1106	>comp52_c1_seq1_5	GAPAQYLDVNYVR
>1107	>comp1072_c0_seq4_6	NFEDINQVQNALR
>1108	>comp1172_c0_seq1_1	IPINALISLER
>1109	>comp1172_c0_seq1_1	NQIDSIDDSLYGLTK
>1110	>comp105006_c0_seq1_4	LATVISPR
>1111	>comp105102_c0_seq1_2	ISRCLFSAAMVLVR
>1112	>comp197_c0_seq1_4	TYVKDVIAPVASTVIQNVNR
>1113	>MMa42689	LLAGGSVSDSDKQTILK
>1114	>MMa42689	VLYTCNYGPAGNIK
>1115	>comp568_c0_seq1_4	DALSCQIIIPQK
>1116	>comp568_c0_seq1_4	DALSCQIIIPQKQVGR
>1117	>comp568_c0_seq1_4	DIDPNKTTTAQMYEK
>1118	>comp568_c0_seq1_4	EALVSNLMGMFEK
>1119	>comp568_c0_seq1_4	ECILSGLLSVSGKK
>1120	>comp568_c0_seq1_4	FNLPSAQLDEYGR
>1121	>comp568_c0_seq1_4	GTGEDFDFSK
>1122	>comp568_c0_seq1_4	KSDIYVSLVSYTHQVAAK
>1123	>comp568_c0_seq1_4	QVYCDPSYVPDR

>1124	>comp568_c0_seq1_4	QVYCDPSYVPDRSEK
>1125	>comp568_c0_seq1_4	SDIYVSLVSYTHQVAAK
>1126	>comp568_c0_seq1_4	SPYLYPLYGLGELPQGFAR
>1127	>comp568_c0_seq1_4	YYGGESASITPLEELFSK
>1128	>comp61_c0_seq1_2	FSDNTAGIAFPNTVCR
>1129	>comp61_c0_seq1_2	MLFNTLDLR
>1130	>comp61_c0_seq1_2	SNEANLAPYIPSQPR
>1131	>MMa42691	AMEDGIVYSGK
>1132	>MMa42691	APSTDDYLLYCQFSDK
>1133	>MMa42691	APSTDDYLLYCQFSDKDSK
>1134	>MMa42691	CSEEYPGLCK
>1135	>MMa42691	DASPQYFEFK
>1136	>MMa42691	DASPQYFEFKEYGVR
>1137	>MMa42691	IGCGVAGYTEANTK
>1138	>MMa42691	IGCGVAGYTEANTKK
>1139	>MMa42691	KVLYTCNYGPAGNMIGSEAYQVGSPCSACPENTK
>1140	>MMa42691	NGICAYLIGR
>1141	>MMa42691	TSSSWNTIGILMK
>1142	>MMa42691	VIAGGSVSDSDKETILK
>1143	>MMa42691	VLYTCNYGPAGNMIGSEAYQVGSPCSACPENTK
>1144	>MMa07107	GYSFTTTAEREIVR
>1145	>MMa07107	IKIIAPPER
>1146	>MMa07107	IWHHTFYNELR
>1147	>MMa07107	LDLAGRDLTDYLMK
>1148	>comp122875_c0_seq1_4	QFPSLVGR
>1149	>comp544_c0_seq1_5	GITEDLPINALVPLK
>1150	>comp544_c0_seq1_5	GITEDLPINALVPLKNLR
>1151	>comp544_c0_seq1_5	ILMLELNHITVLKDPLWK
>1152	>comp544_c0_seq1_5	IYSNVFPPTLK
>1153	>comp544_c0_seq1_5	LHLDILILDDNNIFR
>1154	>comp544_c0_seq1_5	LKILNLSR
>1155	>comp544_c0_seq1_5	LKSLGNSLIHLK
>1156	>comp544_c0_seq1_5	LTLVCEDFENINQVHSALR
>1157	>comp544_c0_seq1_5	SLGNSLIHLKK
>1158	>comp544_c0_seq1_5	SLNVADNQLVEIGEKIK
>1159	>comp544_c0_seq1_5	TLNGELTGLVNLEELYVVQNR
>1160	>comp4077_c0_seq1_2	LTTLDISNNR
>1161	>comp4077_c0_seq1_2	NNQIETLDNSLYNLQELKR
>1162	>comp4077_c0_seq1_2	TLTNELTDLK
>1163	>comp4077_c0_seq1_2	TLTNELTDLKNLK

>1164	>comp1072_c0_seq1_4	ITELKDSLQELR
>1165	>comp1072_c0_seq1_4	LIELAEISR
>1166	>comp16672_c0_seq10_5	TLLDLDKDK
>1167	>comp52_c1_seq1_5	DEQQTILELHNK
>1168	>comp52_c1_seq1_5	DGFCLTLSFR
>1169	>comp52_c1_seq1_5	EGVSKDEQQTILELHNK
>1170	>comp52_c1_seq1_5	FTPESINPFIDDDATGHFTQMAWSSTWK
>1171	>comp52_c1_seq1_5	GANVAGSNSNNQFEVQLDR
>1172	>comp52_c1_seq1_5	GAPAQYLDVNYVR
>1173	>comp52_c1_seq1_5	GPDMDEGDFSR
>1174	>comp52_c1_seq1_5	KGANVAGSNSNNQFEVQLDR
>1175	>comp52_c1_seq1_5	KSHSISYLGLCK
>1176	>comp52_c1_seq1_5	LADQCVFKHDCDECR
>1177	>comp52_c1_seq1_5	LDSEGNEWLPYSIGIPMK
>1178	>comp52_c1_seq1_5	LPPAGDMLEMEWDEELSK
>1179	>comp52_c1_seq1_5	QPMQISLK
>1180	>comp52_c1_seq1_5	SHSISYLGLCK
>1181	>comp52_c1_seq1_5	TESSSDCQSQVEGANKWQTR
>1182	>comp52_c1_seq1_5	TVTLNGGETTTLK
>1183	>comp52_c1_seq1_5	VGCGYVSFEK
>1184	>comp52_c1_seq1_5	VGCGYVSFEKR
>1185	>comp52_c1_seq1_5	VLGERGPDMDEGDFSR
>1186	>comp9550_c0_seq1_6	AVFPSIVGRPR
>1187	>comp9550_c0_seq1_6	DLYANTVLSGGTTMYPGIADR
>1188	>comp9550_c0_seq1_6	GYSFTTTAEREIVR
>1189	>comp9550_c0_seq1_6	HQGVMVGMGQKDSYVGDEAQSK
>1190	>comp9550_c0_seq1_6	IKIIAPPER
>1191	>comp9550_c0_seq1_6	IWHHTFYNELR
>1192	>comp9550_c0_seq1_6	KDLYANTVLSGGTTMYPGIADR
>1193	>comp9550_c0_seq1_6	LDLAGRDLTDYLMK
>1194	>comp9550_c0_seq1_6	QEYDESGPSIVHR
>1195	>comp9550_c0_seq1_6	SYELPDGQVITIGNER
>1196	>comp9550_c0_seq1_6	VAPEEHPVLLTEAPLNPK
>1197	>MMa35583	LGISSDLFNK
>1198	>MMa35583	LGISSDLFNKLDVGK
>1199	>MMa35583	LIELAEISR
>1200	>MMa35583	LNSLGNSLSNLEMLK
>1201	>comp105006_c0_seq1_4	LATVISPR
>1202	>MMa15302	AVFPSIVGRPR
>1203	>MMa15302	DLYANTVLSGGTTMYPGIADR
>1204	>MMa15302	EITALAPSTMK
>1205	>MMa15302	GYSFTTTAEREIVR

>1206	>MMa15302	HQGVMVGMGQKDSYVGDEAQSK
>1207	>MMa15302	IKIIAPPER
>1208	>MMa15302	IWHHTFYNELR
>1209	>MMa15302	KDLYANTVLSGGTTMYPGIADR
>1210	>MMa15302	LCYVALDFEQEMATAASSSSLEK
>1211	>MMa15302	LDLAGRDLTDYLMK
>1212	>MMa15302	MQKEITALAPSTMK
>1213	>MMa15302	QEYDESGPSIVHR
>1214	>MMa15302	SYELPDGQVITIGNER
>1215	>MMa15302	VAPEEHPVLLTEAPLNPK
>1216	>comp197_c0_seq1_4	LSHPNTLIYPYINYILPGTK
>1217	>comp431_c0_seq1_5	EKLDYSEMLISR
>1218	>comp431_c0_seq1_5	FKEIYVGIK
>1219	>comp431_c0_seq1_5	LFYLYGADPVIGISVAPNDK
>1220	>comp431_c0_seq1_5	STFKDSYNQIFDEFGGFPLVK
>1221	>MMa42689	CSEEYPGLCK
>1222	>MMa42689	NGICAYLIGR
>1223	>MMa42689	VLYTCNYGPAGNIK
>1224	>comp2191_c0_seq1_4	YVLPDGSVLEIGPAR
>1225	>comp61_c0_seq1_2	SNEANLAPYIPSQPR
>1226	>comp1024_c0_seq1_4	VLLISFDGFR
>1227	>comp154040_c0_seq1_3	IMNILGLVDIVKSNILNLIV
>1228	>MMa31039	IISNEELNVIAGDVSPGK
>1229	>MMa31039	IISNEELNVIAGDVSPGKGK
>1230	>MMa31039	TKPAMFTDIR
>1231	>MMa50337	ADIVMLITK
>1232	>MMa50337	ADIVMLITKRPLGDYDEK
>1233	>MMa50337	ADTVAHESAHLLGCDHDGEGDER
>1234	>MMa50337	DCPASDGYIMGDR
>1235	>MMa50337	FSDNTAGIAFPNTVCR
>1236	>MMa50337	GIAAEETLKNMAK
>1237	>MMa50337	KFSDNTAGIAFPNTVCR
>1238	>MMa50337	MLFNTLDLR
>1239	>MMa50337	NSGDSMEVYLANLMNAVK
>1240	>MMa50337	SNEANLAPYIPSQPR
>1241	>MMa50337	STDILVLR
>1242	>MMa50337	TGSKDCPASDGYIMGDR
>1243	>MMa50337	VVIIGIIK
>1244	>MMa50337	YGLVTDHSKLNER
>1245	>comp106427_c0_seq1_6	VATVPSIR
>1246	>comp544_c0_seq1_5	LTLVCEDFENINQVHSALR
>1247	>MMa38659	SFLIFPGTK

>1248	>MMa05656	CPASLGHFMGSR
>1249	>MMa05656	ILGLAYPGTLCNK
>1250	>MMa05656	VAIIGIIK
>1251	>MMa05656	VQTIMDSLDLGLK
>1252	>MMa56292	ISYYSSDAMETISR
>1253	>MMa56292	VTVQQLER
>1254	>comp661_c0_seq1_2	ADIVPLITTRPLGTVHDDGSFDLK
>1255	>comp661_c0_seq1_2	ADTVAHESAHTLGCDHDGDGDDITGSK
>1256	>comp661_c0_seq1_2	CGKFILVADDSQANER
>1257	>comp661_c0_seq1_2	FILVADDSQANER
>1258	>comp661_c0_seq1_2	FYCISTDK
>1259	>comp661_c0_seq1_2	FYCISTDKLIQK
>1260	>comp661_c0_seq1_2	GLDAGYTLDKMSK
>1261	>comp661_c0_seq1_2	LAPYIATYPK
>1262	>comp661_c0_seq1_2	YAGLAYLNSACK
>1263	>comp4077_c0_seq1_2	LTTLDISNNR
>1264	>comp9550_c0_seq1_6	DLYANTVLSGGTTMYPGIADR
>1265	>comp52_c1_seq1_5	YLFNCDFKTESSSDCQSQVEGANK
>1266	>comp66592_c0_seq1_6	VVLLGLIK
>1267	>comp347_c1_seq1_6	ILGLAYPGTLCNK
>1268	>comp347_c1_seq1_6	VAIIGIIK
>1269	>comp347_c1_seq1_6	VQTIMDSLDLGLK
>1270	>comp105006_c0_seq1_4	LATVISPR
>1271	>MMa15302	DLYANTVLSGGTTMYPGIADR
>1272	>MMa15302	LCYVALDFEQEMATAASSSSLEK
>1273	>comp61_c0_seq1_2	ADIVMLITK
>1274	>comp61_c0_seq1_2	ADIVMLITKRPLGDYDEK
>1275	>comp61_c0_seq1_2	ADTVAHESAHLLGCDHDGEGDER
>1276	>comp61_c0_seq1_2	DCPASDGYIMGDR
>1277	>comp61_c0_seq1_2	FSDNTAGIAFPNTVCR
>1278	>comp61_c0_seq1_2	GIAAEETLKNMAK
>1279	>comp61_c0_seq1_2	KFSDNTAGIAFPNTVCR
>1280	>comp61_c0_seq1_2	MLFNTLDLR
>1281	>comp61_c0_seq1_2	NSGDSMEVYLANLMNAVK
>1282	>comp61_c0_seq1_2	SNEANLAPYIPSQPR
>1283	>comp61_c0_seq1_2	STDILVLR
>1284	>comp61_c0_seq1_2	TGSKDCPASDGYIMGDR
>1285	>comp61_c0_seq1_2	VVIIGIIK
>1286	>comp61_c0_seq1_2	YGLVTDHSKLNER
>1287	>MMa50337	ADIVMLITK
>1288	>MMa50337	ADTVAHESAHLLGCDHDGEGDER
>1289	>MMa50337	DCPASDGYIMGDR

>1290	>MMa50337	FSDNTAGIAFPNTVCR
>1291	>MMa50337	GIAAEETLKNMAK
>1292	>MMa50337	KFSDNTAGIAFPNTVCR
>1293	>MMa50337	MLFNTLDLR
>1294	>MMa50337	NSGDSMEVYLANLMNAVK
>1295	>MMa50337	SNEANLAPYIPSQPR
>1296	>MMa50337	SVRNQLQVAESR
>1297	>MMa50337	TGSKDCPASDGYIMGDR
>1298	>MMa50337	VVIIGIIK
>1299	>MMa50337	YGLVTDHSKLNER
>1300	>MMa05656	ILGLAYPGTLCNK
>1301	>MMa05656	VAIIGIIK
>1302	>MMa05656	VQTIMDSLDLGLK
>1303	>MMa31039	IISNEELNVIAGDVSPGK
>1304	>MMa31039	IISNEELNVIAGDVSPGKGK
>1305	>MMa55199	THINIVVIGHVDSGK
>1306	>MMa56292	VTVQQLER
>1307	>MMa38659	MVNKCNELLYR
>1308	>comp661_c0_seq1_2	FILVADDSQANER
>1309	>comp661_c0_seq1_2	FYCISTDKLIQK
>1310	>comp661_c0_seq1_2	GLDAGYTLDK
>1311	>comp661_c0_seq1_2	GLDAGYTLDKMSK
>1312	>comp661_c0_seq1_2	LTGEYEAKLAPYIATYPK
>1313	>comp661_c0_seq1_2	YAGLAYLNSACK
>1314	>comp661_c0_seq1_2	YAGLAYLNSACKK
>1315	>comp544_c0_seq1_5	LTLVCEDFENINQVHSALR
>1316	>comp52_c1_seq1_5	GAPAQYLDVNYVR
>1317	>comp66592_c0_seq1_6	VVLLGLIK
>1318	>comp1172_c0_seq1_1	NQIDSIDDSLYGLTK
>1319	>comp347_c1_seq1_6	ILGLAYPGTLCNK
>1320	>comp347_c1_seq1_6	VAIIGIIK
>1321	>comp347_c1_seq1_6	VQTIMDSLDLGLK
>1322	>comp105006_c0_seq1_4	LATVISPR
>1323	>comp61_c0_seq1_2	ADIVMLITK
>1324	>comp61_c0_seq1_2	ADTVAHESAHLLGCDHDGEGDER
>1325	>comp61_c0_seq1_2	DCPASDGYIMGDR
>1326	>comp61_c0_seq1_2	FSDNTAGIAFPNTVCR
>1327	>comp61_c0_seq1_2	GIAAEETLKNMAK
>1328	>comp61_c0_seq1_2	KFSDNTAGIAFPNTVCR
>1329	>comp61_c0_seq1_2	MLFNTLDLR
>1330	>comp61_c0_seq1_2	NSGDSMEVYLANLMNAVK
>1331	>comp61_c0_seq1_2	SNEANLAPYIPSQPR

>1332	>comp61_c0_seq1_2	SVRNQLQVAESR
>1333	>comp61_c0_seq1_2	TGSKDCPASDGYIMGDR
>1334	>comp61_c0_seq1_2	VVIIGIIK
>1335	>comp61_c0_seq1_2	YGLVTDHSKLNER
>1336	>comp1024_c0_seq1_4	FGPLSQAVGK
>1337	>comp1024_c0_seq1_4	TYHTPNFDYLAK
>1338	>MMa50337	ADIVMLITK
>1339	>MMa50337	ADIVMLITKRPLGDYDEK
>1340	>MMa50337	ADTVAHESAHLLGCDHDGEGDER
>1341	>MMa50337	DCPASDGYIMGDR
>1342	>MMa50337	FSDNTAGIAFPNTVCR
>1343	>MMa50337	GIAAEETLKNMAK
>1344	>MMa50337	KFSDNTAGIAFPNTVCR
>1345	>MMa50337	MLFNTLDLR
>1346	>MMa50337	NSGDSMEVYLANLMNAVK
>1347	>MMa50337	SNEANLAPYIPSQPR
>1348	>MMa50337	STDILVLR
>1349	>MMa50337	SVRNQLQVAESR
>1350	>MMa50337	TGSKDCPASDGYIMGDR
>1351	>MMa50337	YGLVTDHSKLNER
>1352	>MMa05656	ILGLAYPGTLCNK
>1353	>MMa31039	IISNEELNVIAGDVSPGK
>1354	>MMa31039	IISNEELNVIAGDVSPGKGK
>1355	>MMa31039	ISAGVLSFSYENDCILEK
>1356	>MMa38659	MVNKCNELLYR
>1357	>MMa38659	SFLIFPGTK
>1358	>comp661_c0_seq1_2	ADIVPLITTRPLGTVHDDGSFDLK
>1359	>comp661_c0_seq1_2	FILVADDSQANER
>1360	>comp661_c0_seq1_2	FYCISTDKLIQK
>1361	>comp661_c0_seq1_2	GLDAGYTLDK
>1362	>comp661_c0_seq1_2	GLDAGYTLDKMSK
>1363	>comp661_c0_seq1_2	LAPYIATYPK
>1364	>comp661_c0_seq1_2	LAPYIATYPKGLDAGYTLDK
>1365	>comp661_c0_seq1_2	VAINGIIK
>1366	>comp661_c0_seq1_2	YAGLAYLNSACK
>1367	>comp661_c0_seq1_2	YAGLAYLNSACKK
>1368	>comp6508_c1_seq1_2	VALNGIIK
>1369	>comp1172_c0_seq1_1	NQIDSIDDSLYGLTK
>1370	>comp105006_c0_seq1_4	LATVISPR
>1371	>comp61_c0_seq1_2	ADIVMLITK
>1372	>comp61_c0_seq1_2	ADIVMLITKRPLGDYDEK
>1373	>comp61_c0_seq1_2	ADTVAHESAHLLGCDHDGEGDER

>1374	>comp61_c0_seq1_2	DCPASDGYIMGDR
>1375	>comp61_c0_seq1_2	FSDNTAGIAFPNTVCR
>1376	>comp61_c0_seq1_2	GIAAEETLKNMAK
>1377	>comp61_c0_seq1_2	KFSDNTAGIAFPNTVCR
>1378	>comp61_c0_seq1_2	MLFNTLDLR
>1379	>comp61_c0_seq1_2	NSGDSMEVYLANLMNAVK
>1380	>comp61_c0_seq1_2	SNEANLAPYIPSQPR
>1381	>comp61_c0_seq1_2	STDILVLR
>1382	>comp61_c0_seq1_2	SVRNQLQVAESR
>1383	>comp61_c0_seq1_2	TGSKDCPASDGYIMGDR
>1384	>comp61_c0_seq1_2	VVIIGIIKHTK
>1385	>comp61_c0_seq1_2	YGLVTDHSKLNER
>1386	>MMa41877	FLYEEFFPSLIGR
>1387	>MMa41877	KLEDFTIGIGNLDYVSK
>1388	>MMa41877	LEDFTIGIGNLDYVSK
>1389	>MMa41877	LITLSLSANK
>1390	>MMa41877	LITLSLSANKLQEVK
>1391	>MMa41877	LVAISLEGNR
>1392	>MMa41877	LVAISLEGNRIR
>1393	>MMa41877	LVHPEAFSNHEKLITLSLSANK
>1394	>MMa41877	SLPEDMFIGMPK
>1395	>MMa41877	SLQILHIDYINLK
>1396	>MMa41877	SMLPNPAKELR
>1397	>MMa41877	YLILSYNKLK
>1398	>comp365_c0_seq3_2	DIMEDCRCNEFSR
>1399	>comp1201_c0_seq2_1	YLFNFQMK
>1400	>MMa04250	SLGVAVEGVPDQYADGK
>1401	>comp1468_c0_seq1_6	AWDMWSFDGNNYQLR
>1402	>comp1468_c0_seq1_6	ELGPIKDDTVLILNQSSGEIIR
>1403	>comp1468_c0_seq1_6	FTPNNEKPSIVLGEEFTPGSDR
>1404	>comp1468_c0_seq1_6	ILCYSVENNDSFGK
>1405	>comp1468_c0_seq1_6	ISVEEEINWPHK
>1406	>comp1468_c0_seq1_6	ISVEEEINWPHKSTLK
>1407	>comp1468_c0_seq1_6	MIIPHSLSMIEEK
>1408	>comp1468_c0_seq1_6	MIIPHSLSMIEEKK
>1409	>comp1468_c0_seq1_6	MNNGNIINTWHSHK
>1410	>comp1468_c0_seq1_6	QVSGIDVDKLGNVHVLHR
>1411	>MMa05656	ILGLAYPGTLCNK
>1412	>MMa05656	VQTIMDSLDLGLK
>1413	>MMa31039	FVLTAAHCVYDDER
>1414	>MMa31039	IISNEELNVIAGDVSPGK
>1415	>MMa31039	IISNEELNVIAGDVSPGKGK

>1416	>MMa31039	ISAGVLSFSYENDCILEK
>1417	>MMa31039	KFVLTAAHCVYDDER
>1418	>MMa31039	TKPAMFTDIR
>1419	>comp545_c0_seq1_1	AGVHLLGTNGAIPAHK
>1420	>comp545_c0_seq1_1	DAPPLELPK
>1421	>comp545_c0_seq1_1	ESYLCTSFK
>1422	>comp545_c0_seq1_1	FINGGTDNSGIVLTLLPGDDQSVTK
>1423	>comp545_c0_seq1_1	FNMLMPDVQPLQK
>1424	>comp545_c0_seq1_1	GSGINYLVLQVHYADVTK
>1425	>comp545_c0_seq1_1	HDPLEPQMFYPVENQDLTIEK
>1426	>comp545_c0_seq1_1	IEQPVTLHPFAFR
>1427	>comp14308_c0_seq1_2	KNVASTIYQR
>1428	>comp52_c1_seq1_5	GANVAGSNSNNQFEVQLDR
>1429	>comp52_c1_seq1_5	LADQCVFKHDCDECR
>1430	>comp52_c1_seq1_5	SHSISYLGLCK
>1431	>comp52_c1_seq1_5	TESSSDCQSQVEGANKWQTR
>1432	>comp52_c1_seq1_5	TVTLNGGETTTLK
>1433	>comp52_c1_seq1_5	VGCGYVSFEK
>1434	>comp101207_c0_seq1_4	EHVDILIK
>1435	>comp2213_c0_seq1_5	VVGILLGSWK
>1436	>comp1965_c0_seq1_5	MPLIGLGTFQSNK
>1437	>comp1965_c0_seq1_5	TPAQILLR
>1438	>comp347_c1_seq1_6	ILGLAYPGTLCNK
>1439	>comp347_c1_seq1_6	VQTIMDSLDLGLK
>1440	>comp105006_c0_seq1_4	LATVISPR
>1441	>MMa21329	DASYDEIKAAVK
>1442	>MMa21329	GAGQNIIPASTGAAK
>1443	>MMa21329	LIAWYDNEYGYSNR
>1444	>MMa21329	VPTPDVSVVDLTCR
>1445	>comp61_c0_seq1_2	FSDNTAGIAFPNTVCR
>1446	>comp61_c0_seq1_2	GIAAEETLKNMAK
>1447	>comp61_c0_seq1_2	MLFNTLDLR
>1448	>comp61_c0_seq1_2	SNEANLAPYIPSQPR
>1449	>comp61_c0_seq1_2	TGSKDCPASDGYIMGDR
>1450	>comp61_c0_seq1_2	YGLVTDHSKLNER
>1451	>MMa50337	ADIVMLITK
>1452	>MMa50337	ADIVMLITKRPLGDYDEK
>1453	>MMa50337	DCPASDGYIMGDR
>1454	>MMa50337	FSDNTAGIAFPNTVCR
>1455	>MMa50337	GIAAEETLKNMAK
>1456	>MMa50337	IKVVIIGIIK
>1457	>MMa50337	KFSDNTAGIAFPNTVCR

>1458	>MMa50337	KNSGDSMEVYLANLMNAVK
>1459	>MMa50337	MLFNTLDLR
>1460	>MMa50337	NSGDSMEVYLANLMNAVK
>1461	>MMa50337	SNEANLAPYIPSQPR
>1462	>MMa50337	STDILVLR
>1463	>MMa50337	SVRNQLQVAESR
>1464	>MMa50337	TGSKDCPASDGYIMGDR
>1465	>MMa50337	VVIIGIIK
>1466	>MMa50337	YGLVTDHSKLNER
>1467	>MMa05656	ILGLAYPGTLCNK
>1468	>MMa05656	VQTIMDSLDLGLK
>1469	>comp66592_c0_seq1_6	LKVVLLGLIK
>1470	>comp66592_c0_seq1_6	VVLLGLIK
>1471	>comp61_c0_seq1_2	ADIVMLITK
>1472	>comp61_c0_seq1_2	ADIVMLITKRPLGDYDEK
>1473	>comp61_c0_seq1_2	DCPASDGYIMGDR
>1474	>comp61_c0_seq1_2	FSDNTAGIAFPNTVCR
>1475	>comp61_c0_seq1_2	GIAAEETLKNMAK
>1476	>comp61_c0_seq1_2	IKVVIIGIIK
>1477	>comp61_c0_seq1_2	KFSDNTAGIAFPNTVCR
>1478	>comp61_c0_seq1_2	KNSGDSMEVYLANLMNAVK
>1479	>comp61_c0_seq1_2	MLFNTLDLR
>1480	>comp61_c0_seq1_2	NSGDSMEVYLANLMNAVK
>1481	>comp61_c0_seq1_2	SNEANLAPYIPSQPR
>1482	>comp61_c0_seq1_2	STDILVLR
>1483	>comp61_c0_seq1_2	SVRNQLQVAESR
>1484	>comp61_c0_seq1_2	TGSKDCPASDGYIMGDR
>1485	>comp61_c0_seq1_2	VVIIGIIK
>1486	>comp61_c0_seq1_2	VVIIGIIKHTK
>1487	>comp61_c0_seq1_2	YGLVTDHSKLNER
>1488	>comp661_c0_seq1_2	FILVADDSQANER
>1489	>comp105006_c0_seq1_4	LATVISPR
>1490	>comp347_c1_seq1_6	ILGLAYPGTLCNK
>1491	>comp347_c1_seq1_6	VQTIMDSLDLGLK
>1492	>comp38617_c0_seq1_5	YRSPTLDLR
>1493	>comp22670_c0_seq1_5	VSLVMILKEK
>1494	>MMa50337	ADIVMLITK
>1495	>MMa50337	ADIVMLITKRPLGDYDEK
>1496	>MMa50337	DCPASDGYIMGDR
>1497	>MMa50337	FSDNTAGIAFPNTVCR
>1498	>MMa50337	GIAAEETLKNMAK
>1499	>MMa50337	KFSDNTAGIAFPNTVCR

>1500	>MMa50337	KNSGDSMEVYLANLMNAVK
>1501	>MMa50337	MLFNTLDLR
>1502	>MMa50337	MLFNTLDLRIK
>1503	>MMa50337	NSGDSMEVYLANLMNAVK
>1504	>MMa50337	SNEANLAPYIPSQPR
>1505	>MMa50337	STDILVLR
>1506	>MMa50337	SVRNQLQVAESR
>1507	>MMa50337	VVIIGIIK
>1508	>MMa50337	YGLVTDHSKLNER
>1509	>MMa38659	MVEMIEVNTYIGGK
>1510	>MMa38659	MVEMIEVNTYIGGKK
>1511	>MMa38659	MVNKCNELLYR
>1512	>comp661_c0_seq1_2	FILVADDSQANER
>1513	>MMa05656	ILGLAYPGTLCNK
>1514	>comp66592_c0_seq1_6	VVLLGLIK
>1515	>comp347_c1_seq1_6	ILGLAYPGTLCNK
>1516	>comp105006_c0_seq1_4	LATVISPR
>1517	>MMa27185	EYICAGVSDGTK
>1518	>comp61_c0_seq1_2	ADIVMLITK
>1519	>comp61_c0_seq1_2	ADIVMLITKRPLGDYDEK
>1520	>comp61_c0_seq1_2	DCPASDGYIMGDR
>1521	>comp61_c0_seq1_2	FSDNTAGIAFPNTVCR
>1522	>comp61_c0_seq1_2	GIAAEETLKNMAK
>1523	>comp61_c0_seq1_2	KFSDNTAGIAFPNTVCR
>1524	>comp61_c0_seq1_2	KNSGDSMEVYLANLMNAVK
>1525	>comp61_c0_seq1_2	MLFNTLDLR
>1526	>comp61_c0_seq1_2	MLFNTLDLRIK
>1527	>comp61_c0_seq1_2	NSGDSMEVYLANLMNAVK
>1528	>comp61_c0_seq1_2	SNEANLAPYIPSQPR
>1529	>comp61_c0_seq1_2	STDILVLR
>1530	>comp61_c0_seq1_2	SVRNQLQVAESR
>1531	>comp61_c0_seq1_2	VVIIGIIK
>1532	>comp61_c0_seq1_2	VVIIGIIKHTK
>1533	>comp61_c0_seq1_2	YGLVTDHSKLNER
>1534	>MMa50337	ADIVMLITK
>1535	>MMa50337	ADIVMLITKRPLGDYDEK
>1536	>MMa50337	ADTVAHESAHLLGCDHDGEGDER
>1537	>MMa50337	ADTVAHESAHLLGCDHDGEGDERTGSK
>1538	>MMa50337	DCPASDGYIMGDR
>1539	>MMa50337	DCPASDGYIMGDRNK
>1540	>MMa50337	FSDNTAGIAFPNTVCR
>1541	>MMa50337	FSDNTAGIAFPNTVCRQCYK

>1542	>MMa50337	GIAAEETLKNMAK
>1543	>MMa50337	IKVVIIGIIK
>1544	>MMa50337	KFSDNTAGIAFPNTVCR
>1545	>MMa50337	KNSGDSMEVYLANLMNAVK
>1546	>MMa50337	MLFNTLDLR
>1547	>MMa50337	MLFNTLDLRIK
>1548	>MMa50337	NSGDSMEVYLANLMNAVK
>1549	>MMa50337	SNEANLAPYIPSQPR
>1550	>MMa50337	STDILVLR
>1551	>MMa50337	SVRNQLQVAESR
>1552	>MMa50337	TGSKDCPASDGYIMGDR
>1553	>MMa50337	YGLVTDHSKLNER
>1554	>MMa05656	ILGLAYPGTLCNK
>1555	>MMa31039	IISNEELNVIAGDVSPGK
>1556	>MMa38659	HESSFLKDLLSIN
>1557	>MMa38659	KLVDCYLYGDSYIIGK
>1558	>MMa38659	LVDCYLYGDSYIIGK
>1559	>MMa38659	MLEMVPVK
>1560	>MMa38659	MVEMIEVNTYIGGK
>1561	>MMa38659	MVEMIEVNTYIGGKK
>1562	>MMa38659	MVNKCNELLYR
>1563	>MMa38659	SFLIFPGTK
>1564	>comp661_c0_seq1_2	FILVADDSQANER
>1565	>comp4077_c0_seq1_2	LTTLDISNNR
>1566	>MMa04441	NYDVNTILFR
>1567	>comp13064_c0_seq1_5	SSELLVIR
>1568	>comp66592_c0_seq1_6	LKVVLLGLIK
>1569	>comp105006_c0_seq1_4	LATVISPR
>1570	>comp61_c0_seq1_2	ADIVMLITK
>1571	>comp61_c0_seq1_2	ADIVMLITKRPLGDYDEK
>1572	>comp61_c0_seq1_2	ADTVAHESAHLLGCDHDGEGDER
>1573	>comp61_c0_seq1_2	ADTVAHESAHLLGCDHDGEGDERTGSK
>1574	>comp61_c0_seq1_2	DCPASDGYIMGDR
>1575	>comp61_c0_seq1_2	DCPASDGYIMGDRNK
>1576	>comp61_c0_seq1_2	FSDNTAGIAFPNTVCR
>1577	>comp61_c0_seq1_2	FSDNTAGIAFPNTVCRQCYK
>1578	>comp61_c0_seq1_2	GIAAEETLKNMAK
>1579	>comp61_c0_seq1_2	IKVVIIGIIK
>1580	>comp61_c0_seq1_2	KFSDNTAGIAFPNTVCR
>1581	>comp61_c0_seq1_2	KNSGDSMEVYLANLMNAVK
>1582	>comp61_c0_seq1_2	MLFNTLDLR
>1583	>comp61_c0_seq1_2	MLFNTLDLRIK

>1584	>comp61_c0_seq1_2	NSGDSMEVYLANLMNAVK
>1585	>comp61_c0_seq1_2	SNEANLAPYIPSQPR
>1586	>comp61_c0_seq1_2	STDILVLR
>1587	>comp61_c0_seq1_2	SVRNQLQVAESR
>1588	>comp61_c0_seq1_2	TGSKDCPASDGYIMGDR
>1589	>comp61_c0_seq1_2	YGLVTDHSKLNER
>1590	>MMa33253	QEPGGDGTEIWNGIK
>1591	>MMa33253	YDPSVQPQMLAR
>1592	>MMa17865	SELWNYNTNK
>1593	>MMa17865	VWCVINNESCNSECK
>1594	>MMa17864	IWCVINNESCNSECK
>1595	>MMa02192	VSCLWGNEGCNKECR
>1596	>comp627_c0_seq1_2	ADLSNNLITR
>1597	>comp627_c0_seq1_2	FGLFLFFPSLEK
>1598	>MMa05656	ILGLAYPGTLCNK
>1599	>MMa05656	VQTIMDSLDLGLK
>1600	>MMa31039	DETPVIQLGWGEFALDSGFSQTLK
>1601	>MMa31039	FVLTAAHCVYDDER
>1602	>MMa31039	FVLTAAHCVYDDERR
>1603	>MMa31039	GFYLNNITCR
>1604	>MMa31039	IISNEELNVIAGDVSPGK
>1605	>MMa31039	IISNEELNVIAGDVSPGKGK
>1606	>MMa31039	ISAGVLSFSYENDCILEK
>1607	>MMa31039	KFVLTAAHCVYDDER
>1608	>MMa31039	KIISNEELNVIAGDVSPGK
>1609	>MMa31039	SEGDRTCHGDSGGPMVITK
>1610	>MMa31039	TICLTDEGYDLK
>1611	>MMa31039	TKPAMFTDIR
>1612	>MMa31039	TQTKDNTICWVQK
>1613	>comp265_c0_seq1_1	LSVIIGIHR
>1614	>comp38_c0_seq1_5	VSECLLNNYCNNICTK
>1615	>MMa38659	MVEMIEVNTYIGGK
>1616	>comp545_c0_seq1_1	IEQPVTLHPFAFR
>1617	>comp1072_c0_seq1_4	LIELAEISR
>1618	>comp16672_c0_seq10_5	TLLDLDKDK
>1619	>comp52_c1_seq1_5	GAPAQYLDVNYVR
>1620	>MMa35583	LIELAEISR
>1621	>comp1172_c0_seq1_1	IPINALISLER
>1622	>comp347_c1_seq1_6	ILGLAYPGTLCNK
>1623	>comp347_c1_seq1_6	VQTIMDSLDLGLK
>1624	>comp105006_c0_seq1_4	LATVISPR
>1625	>MMa08776	ISVSGLGPLNSTVK

>1626	>MMa08776	ISVSGLGPLNSTVKK
>1627	>MMa08776	QSNSFMDAVLQNVK
>1628	>MMa08776	QSNSFMDAVLQNVKTEGR
>1629	>MMa27185	YYAVGIVSFGK
>1630	>MMa17864	SELWAYETNK
>1631	>MMa05656	CPASLGHFMGSR
>1632	>MMa05656	ILGLAYPGTLCNK
>1633	>MMa05656	VAIIGIIK
>1634	>MMa05656	VQTIMDSLDLGLK
>1635	>MMa17865	VWCVINNESCNSECK
>1636	>MMa31039	FVLTAAHCVYDDER
>1637	>MMa31039	FVLTAAHCVYDDERR
>1638	>MMa31039	GNQKISAGVLSFSYENDCILEK
>1639	>MMa31039	IISNEELNVIAGDVSPGK
>1640	>MMa31039	IISNEELNVIAGDVSPGKGK
>1641	>MMa31039	ISAGVLSFSYENDCILEK
>1642	>MMa31039	KFVLTAAHCVYDDER
>1643	>MMa31039	KIISNEELNVIAGDVSPGK
>1644	>MMa31039	SEGDRTCHGDSGGPMVITK
>1645	>MMa31039	TICLTDEGYDLK
>1646	>MMa31039	TKPAMFTDIR
>1647	>MMa31039	TQTKDNTICWVQK
>1648	>comp38_c0_seq1_5	VSECLLNNYCNNICTK
>1649	>comp1072_c0_seq1_4	LIELAEISR
>1650	>comp20_c0_seq1_3	YAVPEGTLR
>1651	>MMa35583	LIELAEISR
>1652	>MMa33253	QEPGGDGTEIWNGIK
>1653	>comp1172_c0_seq1_1	IPINALISLER
>1654	>comp347_c1_seq1_6	ILGLAYPGTLCNK
>1655	>comp347_c1_seq1_6	VAIIGIIK
>1656	>comp347_c1_seq1_6	VQTIMDSLDLGLK
>1657	>comp105006_c0_seq1_4	LATVISPR
>1658	>comp627_c0_seq1_2	ADLSNNLITR
>1659	>comp627_c0_seq1_2	AENLQIVSLKGNR
>1660	>MMa17865	VWCVINNESCNSECK
>1661	>MMa17864	IWCVINNESCNSECK
>1662	>MMa08776	ISVSGLGPLNSTVK
>1663	>MMa08776	ISVSGLGPLNSTVKK
>1664	>MMa08776	QSNSFMDAVLQNVK
>1665	>MMa08776	QSNSFMDAVLQNVKTEGR
>1666	>comp627_c0_seq1_2	ADLSNNLITR
>1667	>MMa05656	ILGLAYPGTLCNK

>1668	>MMa05656	VAIIGIIK
>1669	>MMa05656	VQTIMDSLDLGLK
>1670	>MMa31039	DETPVIQLGWGEFALDSGFSQTLK
>1671	>MMa31039	FVLTAAHCVYDDER
>1672	>MMa31039	FVLTAAHCVYDDERR
>1673	>MMa31039	GNQKISAGVLSFSYENDCILEK
>1674	>MMa31039	IISNEELNVIAGDVSPGK
>1675	>MMa31039	IISNEELNVIAGDVSPGKGK
>1676	>MMa31039	ISAGVLSFSYENDCILEK
>1677	>MMa31039	KFVLTAAHCVYDDER
>1678	>MMa31039	KIISNEELNVIAGDVSPGK
>1679	>MMa31039	TICLTDEGYDLK
>1680	>MMa31039	TKPAMFTDIR
>1681	>MMa31039	TQTKDNTICWVQK
>1682	>MMa31039	YLPVSEIYTYNGYNGNWVNDIAILK
>1683	>comp265_c0_seq1_1	LSVIIGIHR
>1684	>comp52_c1_seq1_5	GAPAQYLDVNYVR
>1685	>comp52_c1_seq1_5	SHSISYLGLCK
>1686	>comp52_c1_seq1_5	VGCGYVSFEK
>1687	>MMa33253	QEPGGDGTEIWNGIK
>1688	>MMa33253	YDPSVQPQMLAR
>1689	>comp347_c1_seq1_6	ILGLAYPGTLCNK
>1690	>comp347_c1_seq1_6	VAIIGIIK
>1691	>comp347_c1_seq1_6	VQTIMDSLDLGLK
>1692	>comp105006_c0_seq1_4	LATVISPR
>1693	>MMa42691	IGCGVAGYTEANTK
>1694	>MMa42691	VIAGGSVSDSDKETILK
>1695	>MMa27185	EYICAGVSDGTK
>1696	>MMa27185	GTDCGGALITNR
>1697	>MMa27185	YYAVGIVSFGK
>1698	>MMa27185	YYAVGIVSFGKR
>1699	>comp10779_c0_seq1_6	EGQYILSLLNK
>1700	>comp1581_c0_seq1_5	GIVLSGPATCTK
>1701	>comp49065_c0_seq1_5	VALIAVIK
>1702	>MMa05656	ILGLAYPGTLCNK
>1703	>MMa05656	VQTIMDSLDLGLK
>1704	>MMa31039	FVLTAAHCVYDDER
>1705	>MMa31039	GNQKISAGVLSFSYENDCILEK
>1706	>MMa31039	IISNEELNVIAGDVSPGK
>1707	>MMa31039	IISNEELNVIAGDVSPGKGK
>1708	>MMa31039	ISAGVLSFSYENDCILEK
>1709	>MMa31039	KFVLTAAHCVYDDER

>1710	>MMa31039	KIISNEELNVIAGDVSPGK
>1711	>MMa31039	TICLTDEGYDLK
>1712	>MMa31039	TKPAMFTDIR
>1713	>MMa31039	TQTKDNTICWVQK
>1714	>comp1072_c0_seq1_4	LIELAEISR
>1715	>comp627_c0_seq1_2	ADLSNNLITR
>1716	>comp627_c0_seq1_2	AENLQIVSLKGNR
>1717	>MMa35583	LIELAEISR
>1718	>MMa30301	GIVLSGPATCTK
>1719	>comp1172_c0_seq1_1	IPINALISLER
>1720	>comp347_c1_seq1_6	ILGLAYPGTLCNK
>1721	>comp347_c1_seq1_6	VQTIMDSLDLGLK
>1722	>comp105006_c0_seq1_4	LATVISPR
>1723	>MMa27185	YYAVGIVSFGK
>1724	>comp61_c0_seq1_2	SNEANLAPYIPSQPR
>1725	>MMa08776	GNTAFAAFTTSMNR
>1726	>MMa08776	ISVSGLGPLNSTVK
>1727	>comp1581_c0_seq1_5	CYIFIIKMESNESYIINNSGLK
>1728	>comp1581_c0_seq1_5	GIVLSGPATCTK
>1729	>comp1581_c0_seq1_5	VFYEMAPSLDK
>1730	>comp1581_c0_seq1_5	VFYEMAPSLDKTFK
>1731	>comp49065_c0_seq1_5	VALIAVIK
>1732	>MMa05656	ENESSLAPYIPQSPK
>1733	>MMa05656	ILGLAYPGTLCNK
>1734	>MMa05656	VQTIMDSLDLGLK
>1735	>MMa31039	FVLTAAHCVYDDER
>1736	>MMa31039	FVLTAAHCVYDDERR
>1737	>MMa31039	IISNEELNVIAGDVSPGK
>1738	>MMa31039	IISNEELNVIAGDVSPGKGK
>1739	>MMa31039	ISAGVLSFSYENDCILEK
>1740	>MMa31039	KFVLTAAHCVYDDER
>1741	>MMa31039	KIISNEELNVIAGDVSPGK
>1742	>MMa31039	TICLTDEGYDLK
>1743	>MMa31039	TKPAMFTDIR
>1744	>MMa31039	TQTKDNTICWVQK
>1745	>comp627_c0_seq1_2	ADLSNNLITR
>1746	>comp627_c0_seq1_2	AENLQIVSLKGNR
>1747	>comp52_c1_seq1_5	VGCGYVSFEK
>1748	>MMa33253	YDPSVQPQMLAR
>1749	>MMa30301	GIVLSGPATCTK
>1750	>MMa30301	MESNESYIINNSGLK
>1751	>comp347_c1_seq1_6	ENESSLAPYIPQSPK

>1752	>comp347_c1_seq1_6	ILGLAYPGTLCNK
>1753	>comp347_c1_seq1_6	VQTIMDSLDLGLK
>1754	>comp105006_c0_seq1_4	LATVISPR
>1755	>comp8549_c0_seq1_5	TEQICNVISK
>1756	>MMa27185	DSCQGDSGGPLMLPSEEPNSDISR
>1757	>MMa27185	EYICAGVSDGTK
>1758	>MMa27185	YYAVGIVSFGK
>1759	>MMa27185	YYAVGIVSFGKR
>1760	>MMa28760	YSFTYALVR
>1761	>comp1024_c0_seq1_4	LLETFLLDR
>1762	>comp1024_c0_seq1_4	VLLISFDGFR
>1763	>comp1024_c0_seq1_4	WWGGEPIWVTTR
>1764	>MMa17865	KSELWNYNTNK
>1765	>MMa17865	VWCVINNESCNSECK
>1766	>MMa17864	IWCVINNESCNSECK
>1767	>comp1678_c0_seq2_6	KQVLQIPK
>1768	>MMa05656	ILGLAYPGTLCNK
>1769	>MMa42689	CSEEYPGLCK
>1770	>MMa55199	IGGIGTVPVGR
>1771	>comp38_c0_seq1_5	VSECLLNNYCNNICTK
>1772	>comp545_c0_seq1_1	ESYLCTSFK
>1773	>comp545_c0_seq1_1	IEQPVTLHPFAFR
>1774	>comp1072_c0_seq1_4	AVISLKFLYNLK
>1775	>comp1072_c0_seq1_4	ITELKDSLQELR
>1776	>comp1072_c0_seq1_4	LGISSNLFNK
>1777	>comp1072_c0_seq1_4	LGISSNLFNKLDVGK
>1778	>comp1072_c0_seq1_4	LIELAEISR
>1779	>comp1072_c0_seq1_4	MLTMIYNDLK
>1780	>comp1072_c0_seq1_4	TLTNELTGLK
>1781	>comp1072_c0_seq2_4	CPAYTGPCDCVFSR
>1782	>comp544_c0_seq1_5	LTLVCEDFENINQVHSALR
>1783	>comp1072_c0_seq3_6	LYLPLHDLPDTIFANQGNCLR
>1784	>comp52_c1_seq1_5	GAPAQYLDVNYVR
>1785	>comp52_c1_seq1_5	LADQCVFKHDCDECR
>1786	>comp52_c1_seq1_5	VGCGYVSFEK
>1787	>MMa31039	ISAGVLSFSYENDCILEK
>1788	>comp1072_c0_seq4_6	NFEDINQVQNALR
>1789	>MMa35583	LIELAEISR
>1790	>comp105006_c0_seq1_4	LATVISPR
>1791	>comp197_c0_seq1_4	AKHPDWSPAQIEK
>1792	>comp197_c0_seq1_4	DQPSEYFCK
>1793	>comp197_c0_seq1_4	DVIAPVASTVIQNVNR

>1794	>comp197_c0_seq1_4	HPDWSPAQIEK
>1795	>comp197_c0_seq1_4	ILVNQEETFNGDK
>1796	>comp197_c0_seq1_4	IVIFYESQLGK
>1797	>comp197_c0_seq1_4	LSHPNTLIYPYINYILPGTK
>1798	>comp197_c0_seq1_4	NDIQEANDKLSWLWK
>1799	>comp197_c0_seq1_4	QSTALCPSIYMQESHITK
>1800	>comp197_c0_seq1_4	TVPSMDFKR
>1801	>comp197_c0_seq1_4	TYVKDVIAPVASTVIQNVNR
>1802	>comp197_c0_seq1_4	VAIEEWENSAK
>1803	>comp197_c0_seq1_4	VAIEEWENSAKEWMLK
>1804	>comp197_c0_seq1_4	VVWEVPSIMCSK
>1805	>MMa42691	AMEDGIVYSGK
>1806	>MMa42691	APSTDDYLLYCQFSDKDSK
>1807	>MMa42691	CSEEYPGLCK
>1808	>MMa42691	DASPQYFEFKEYGVR
>1809	>MMa42691	FGPNDASEKPGSFVSFEFSAPGLIFPK
>1810	>MMa42691	IGCGVAGYTEANTK
>1811	>MMa42691	IGCGVAGYTEANTKK
>1812	>MMa42691	KDASPQYFEFK
>1813	>MMa42691	LSKDHTFCMASTCK
>1814	>MMa42691	TSSSWNTIGILMK
>1815	>MMa42691	VIAGGSVSDSDKETILK
>1816	>comp1024_c0_seq1_4	FGPLSQAVGK
>1817	>comp1024_c0_seq1_4	IMPLIAVADPGYLIAR
>1818	>comp1024_c0_seq1_4	LLETFLLDR
>1819	>comp1024_c0_seq1_4	MQEEVKWMPLSGPVAGILPYEGK
>1820	>comp1024_c0_seq1_4	MVETIDKLVGFLLEK
>1821	>comp1024_c0_seq1_4	RIMPLIAVADPGYLIAR
>1822	>comp1024_c0_seq1_4	SGVYFWPASDIPIK
>1823	>comp1024_c0_seq1_4	VLLISFDGFR
>1824	>comp1024_c0_seq1_4	WMPLSGPVAGILPYEGK
>1825	>comp1024_c0_seq1_4	WMPLSGPVAGILPYEGKR
>1826	>comp1024_c0_seq1_4	WWGGEPIWVTTR
>1827	>comp1024_c0_seq1_4	WWGGEPIWVTTRK
>1828	>MMa17865	KSELWNYNTNK
>1829	>MMa17865	VWCVINNESCNSECK
>1830	>MMa42691	AMEDGIVYSGK
>1831	>MMa42691	APSTDDYLLYCQFSDKDSK
>1832	>MMa42691	CSEEYPGLCK
>1833	>MMa42691	DASPQYFEFK
>1834	>MMa42691	DASPQYFEFKEYGVR
>1835	>MMa42691	IGCGVAGYTEANTK

>1836	>MMa42691	IGCGVAGYTEANTKK
>1837	>MMa42691	KDASPQYFEFK
>1838	>MMa42691	KVLYTCNYGPAGNMIGSEAYQVGSPCSACPENTK
>1839	>MMa42691	LSKDHTFCMASTCK
>1840	>MMa42691	NGICAYLIGR
>1841	>MMa42691	TSSSWNTIGILMK
>1842	>MMa42691	VIAGGSVSDSDKETILK
>1843	>MMa42691	VLYTCNYGPAGNMIGSEAYQVGSPCSACPENTK
>1844	>MMa31930	EYQPSQVIVASVQITANHK
>1845	>MMa05656	VQTIMDSLDLGLK
>1846	>MMa17864	SELWAYETNK
>1847	>MMa42689	CSEEYPGLCK
>1848	>MMa42689	NGICAYLIGR
>1849	>MMa55199	IGGIGTVPVGR
>1850	>comp545_c0_seq1_1	ESYLCTSFK
>1851	>comp545_c0_seq1_1	FNMLMPDVQPLQK
>1852	>comp545_c0_seq1_1	IEQPVTLHPFAFR
>1853	>comp1072_c0_seq1_4	ITELKDSLQELR
>1854	>comp1072_c0_seq1_4	LGISSNLFNK
>1855	>comp1072_c0_seq1_4	LGISSNLFNKLDVGK
>1856	>comp1072_c0_seq1_4	LIELAEISR
>1857	>comp1072_c0_seq1_4	MLTMIYNDLK
>1858	>comp1072_c0_seq1_4	TLTNELTGLK
>1859	>MMa35583	LGISSDLFNKLDVGK
>1860	>MMa35583	LIELAEISR
>1861	>comp52_c1_seq1_5	GAPAQYLDVNYVR
>1862	>comp52_c1_seq1_5	KGANVAGSNSNNQFEVQLDR
>1863	>comp52_c1_seq1_5	LADQCVFKHDCDECR
>1864	>comp52_c1_seq1_5	SHSISYLGLCK
>1865	>MMa31039	ISAGVLSFSYENDCILEK
>1866	>comp1072_c0_seq4_6	NFEDINQVQNALR
>1867	>comp1172_c0_seq1_1	CPANTGPCDCFHVSSR
>1868	>comp1172_c0_seq1_1	ELSLAYNPLK
>1869	>comp1172_c0_seq1_1	IPINALISLER
>1870	>comp1172_c0_seq1_1	LCLYDLTIISGK
>1871	>comp1172_c0_seq1_1	MEKIPINALISLER
>1872	>comp1172_c0_seq1_1	MNLNLPLLNIPDSLFSNQK
>1873	>comp1172_c0_seq1_1	MYYKDFEDLSNLK
>1874	>comp1172_c0_seq1_1	NELSSIAYGLEDLNNLEK
>1875	>comp1172_c0_seq1_1	NFKNIIQVQQALR

>1876	>comp1172_c0_seq1_1	NQIDSIDDSLYGLTK
>1877	>comp1172_c0_seq1_1	NVENIIIGEDNYVINK
>1878	>comp105006_c0_seq1_4	LATVISPR
>1879	>comp197_c0_seq1_4	DVIAPVASTVIQNVNR
>1880	>comp197_c0_seq1_4	HPDWSPAQIEK
>1881	>comp197_c0_seq1_4	ILVNQEETFNGDK
>1882	>comp197_c0_seq1_4	IVIFYESQLGK
>1883	>comp197_c0_seq1_4	LSHPNTLIYPYINYILPGTK
>1884	>comp197_c0_seq1_4	TVPSMDFKR
>1885	>comp197_c0_seq1_4	TYVKDVIAPVASTVIQNVNR
>1886	>comp197_c0_seq1_4	VAIEEWENSAK
>1887	>comp197_c0_seq1_4	VAIEEWENSAKEWMLK
>1888	>comp1072_c0_seq2_4	CPAYTGPCDCVFSR
>1889	>comp1024_c0_seq1_4	FGPLSQAVGK
>1890	>comp1024_c0_seq1_4	IMPLIAVADPGYLIAR
>1891	>comp1024_c0_seq1_4	LLETFLLDR
>1892	>comp1024_c0_seq1_4	MVETIDKLVGFLLEK
>1893	>comp1024_c0_seq1_4	RIMPLIAVADPGYLIAR
>1894	>comp1024_c0_seq1_4	VLLISFDGFR
>1895	>comp1024_c0_seq1_4	WMPLSGPVAGILPYEGK
>1896	>comp1024_c0_seq1_4	WMPLSGPVAGILPYEGKR
>1897	>comp1024_c0_seq1_4	WWGGEPIWVTTR
>1898	>MMa42691	APSTDDYLLYCQFSDKDSK
>1899	>MMa42691	IGCGVAGYTEANTKK
>1900	>comp31795_c0_seq1_5	NIIQVKQIVK
>1901	>MMa31930	EYQPSQVIVASVQITANHK
>1902	>comp41_c0_seq1_5	DVNNLTYLIK
>1903	>MMa48694	LTEVIADISER
>1904	>comp1072_c0_seq1_4	IKTLTNELTGLK
>1905	>comp1072_c0_seq1_4	ITELKDSLQELR
>1906	>comp1072_c0_seq1_4	LGISSNLFNK
>1907	>comp1072_c0_seq1_4	LGISSNLFNKLDVGK
>1908	>comp1072_c0_seq1_4	LIELAEISR
>1909	>comp1072_c0_seq1_4	MLTMIYNDLK
>1910	>comp1072_c0_seq1_4	TLTNELTGLK
>1911	>comp1072_c0_seq2_4	CPAYTGPCDCVFSR
>1912	>comp1072_c0_seq3_6	LYLPLHDLPDTIFANQGNCLR
>1913	>comp52_c1_seq1_5	GAPAQYLDVNYVR
>1914	>comp52_c1_seq1_5	LADQCVFKHDCDECR
>1915	>comp52_c1_seq1_5	SHSISYLGLCK
>1916	>comp52_c1_seq1_5	YLFNCDFK
>1917	>MMa31039	IISNEELNVIAGDVSPGK

>1918	>MMa31039	ISAGVLSFSYENDCILEK
>1919	>comp1072_c0_seq4_6	NFEDINQVQNALR
>1920	>MMa35583	LGISSDLFNKLDVGK
>1921	>MMa35583	LIELAEISR
>1922	>comp1172_c0_seq1_1	ELSLAYNPLK
>1923	>comp1172_c0_seq1_1	IPINALISLER
>1924	>comp1172_c0_seq1_1	LCLYDLTIISGK
>1925	>comp1172_c0_seq1_1	MEKIPINALISLER
>1926	>comp1172_c0_seq1_1	MNLNLPLLNIPDSLFSNQK
>1927	>comp1172_c0_seq1_1	NELSSIAYGLEDLNNLEK
>1928	>comp1172_c0_seq1_1	NFKNIIQVQQALR
>1929	>comp1172_c0_seq1_1	NQIDSIDDSLYGLTK
>1930	>comp1172_c0_seq1_1	NVENIIIGEDNYVINK
>1931	>comp197_c0_seq1_4	DVIAPVASTVIQNVNR
>1932	>comp197_c0_seq1_4	ILVNQEETFNGDK
>1933	>comp197_c0_seq1_4	TYVKDVIAPVASTVIQNVNR
>1934	>comp197_c0_seq1_4	VAIEEWENSAK
>1935	>comp1024_c0_seq1_4	FGPLSQAVGK
>1936	>comp1024_c0_seq1_4	GIQSTFTTK
>1937	>comp1024_c0_seq1_4	IMPLIAVADPGYLIAR
>1938	>comp1024_c0_seq1_4	VLLISFDGFR
>1939	>comp1024_c0_seq1_4	WWGGEPIWVTTR
>1940	>MMa42691	DASPQYFEFK
>1941	>MMa42691	DASPQYFEFKEYGVR
>1942	>MMa42691	IGCGVAGYTEANTK
>1943	>MMa42691	IGCGVAGYTEANTKK
>1944	>MMa42691	NGICAYLIGR
>1945	>MMa42691	TSSSWNTIGILMK
>1946	>MMa42691	VIAGGSVSDSDKETILK
>1947	>comp163667_c0_seq1_6	INETFRNVK
>1948	>MMa42689	NGICAYLIGR
>1949	>MMa55199	IGGIGTVPVGR
>1950	>MMa55199	THINIVVIGHVDSGK
>1951	>comp31795_c0_seq1_5	NIIQVKQIVK
>1952	>comp545_c0_seq1_1	ESYLCTSFK
>1953	>comp1072_c0_seq1_4	ITELKDSLQELR
>1954	>comp1072_c0_seq1_4	LGISSNLFNK
>1955	>comp1072_c0_seq1_4	LGISSNLFNKLDVGK
>1956	>comp1072_c0_seq1_4	LIELAEISR
>1957	>comp1072_c0_seq1_4	TLTNELTGLK
>1958	>comp1072_c0_seq2_4	CPAYTGPCDCVFSR
>1959	>comp1072_c0_seq3_6	LYLPLHDLPDTIFANQGNCLR

>1960	>comp52_c1_seq1_5	DGFCLTLSFR
>1961	>comp52_c1_seq1_5	EGVSKDEQQTILELHNK
>1962	>comp52_c1_seq1_5	GANVAGSNSNNQFEVQLDR
>1963	>comp52_c1_seq1_5	GAPAQYLDVNYVR
>1964	>comp52_c1_seq1_5	KGANVAGSNSNNQFEVQLDR
>1965	>comp52_c1_seq1_5	KSHSISYLGLCK
>1966	>comp52_c1_seq1_5	LADQCVFKHDCDECR
>1967	>comp52_c1_seq1_5	QIISGVYKTVTLNGGETTTLK
>1968	>comp52_c1_seq1_5	SHSISYLGLCK
>1969	>comp52_c1_seq1_5	TESSSDCQSQVEGANKWQTR
>1970	>comp52_c1_seq1_5	TVTLNGGETTTLK
>1971	>comp52_c1_seq1_5	VGCGYVSFEK
>1972	>MMa31039	IISNEELNVIAGDVSPGK
>1973	>comp1072_c0_seq4_6	NFEDINQVQNALR
>1974	>comp1172_c0_seq1_1	ELSLAYNPLK
>1975	>comp1172_c0_seq1_1	IPINALISLER
>1976	>comp1172_c0_seq1_1	LCLYDLTIISGK
>1977	>comp1172_c0_seq1_1	LDLSYNSFTK
>1978	>comp1172_c0_seq1_1	NFKNIIQVQQALR
>1979	>comp1172_c0_seq1_1	NQIDSIDDSLYGLTK
>1980	>comp1172_c0_seq1_1	NQIDSIDDSLYGLTKLR
>1981	>comp1172_c0_seq1_1	NVENIIIGEDNYVINK
>1982	>comp105006_c0_seq1_4	LATVISPR
>1983	>comp197_c0_seq1_4	DVIAPVASTVIQNVNR
>1984	>comp197_c0_seq1_4	HPDWSPAQIEK
>1985	>comp197_c0_seq1_4	ILVNQEETFNGDK
>1986	>comp197_c0_seq1_4	IVIFYESQLGK
>1987	>comp197_c0_seq1_4	TYVKDVIAPVASTVIQNVNR
>1988	>comp197_c0_seq1_4	VAIEEWENSAK
>1989	>comp431_c0_seq1_5	ILITYPETIAK
>1990	>comp431_c0_seq1_5	QLNLPDNAR
>1991	>comp804_c0_seq1_5	VNDELNNLFLR
>1992	>comp508_c0_seq1_4	AGVLIGEILK
>1993	>comp508_c0_seq1_4	ILTSPPKTDALLYEGAICPEAK
>1994	>comp508_c0_seq1_4	YSGYLTSNPR
>1995	>MMa04555	VAECLFNNYCNNECTK
>1996	>comp48_c0_seq1_5	LSTPVTFTPHVNR
>1997	>comp774_c0_seq1_5	CHDFENISQVQNALEK
>1998	>comp774_c0_seq1_5	FPSVIDMLDIR
>1999	>comp774_c0_seq1_5	GGIMEEFPINIFLPLK
>2000	>comp774_c0_seq1_5	IHSGILPNTLK
>2001	>comp774_c0_seq1_5	LKCHDFENISQVQNALEK

>2002	>comp774_c0_seq1_5	NIDISKNPLQCTNNVSLVISSLILK
>2003	>comp774_c0_seq1_5	NPLQCTNNVSLVISSLILK
>2004	>comp774_c0_seq1_5	NVQSLNLAGNQLTEIDESFNCLNK
>2005	>comp774_c0_seq1_5	TLNDELTGLR
>2006	>comp105006_c0_seq1_4	LATVISPR
>2007	>MMa04555	VAECLFNNYCNNECTK
>2008	>MMa04555	VYYADKGYCCLLK
>2009	>MMa02192	VSCLWGNEGCNK
>2010	>MMa02192	VSCLWGNEGCNKECR
>2011	>comp105006_c0_seq1_4	LATVISPR
>2012	>comp20_c0_seq1_3	YAVPEGTLR
>2013	>comp19356_c0_seq1_3	TITVLSPR
>2014	>comp106427_c0_seq1_6	VATVPSIR
>2015	>MMa04555	VAECLFNNYCNNECTK
>2016	>MMa04555	VYYADKGYCCLLK
>2017	>comp105006_c0_seq1_4	LATVISPR
>2018	>comp151396_c0_seq1_5	VFWISCIPSANFVLGER
>2019	>comp10876_c0_seq1_2	LSSPASIDKK
>2020	>MMa35530	LTSMSEYACPVIEK
>2021	>comp21_c0_seq1_5	LHLASGGSCQQPAPFVK
>2022	>MMa02192	VSCLWGNEGCNK
>2023	>MMa04555	VAECLFNNYCNNECTK
>2024	>MMa04555	VYYADKGYCCLLK
>2025	>comp105006_c0_seq1_4	LATVISPR
>2026	>MMa35530	LTSMSEYACPVIEK
>2027	>MMa13616	LPDSVPIR
>2028	>comp2261_c0_seq1_6	IGISSIPR
>2029	>MMa04555	VAECLFNNYCNNECTK
>2030	>MMa04555	VYYADKGYCCLLK
>2031	>MMa02192	VSCLWGNEGCNK
>2032	>MMa02192	VSCLWGNEGCNKECR
>2033	>comp105006_c0_seq1_4	LATVISPR
>2034	>MMa20191	YGNACWCIDLPDKVPIR
>2035	>MMa02192	VSCLWGNEGCNK
>2036	>MMa02192	VSCLWGNEGCNKECR
>2037	>MMa04555	VAECLFNNYCNNECTK
>2038	>MMa04555	VYYADKGYCCLLK
>2039	>comp514_c1_seq77_5	CVLSLGNFLISK
>2040	>comp105006_c0_seq1_4	LATVISPR
>2041	>comp201_c0_seq1_3	DGYCVGDASICFCK
>2042	>comp201_c0_seq1_3	IEDGYYLSDENGVFVTCK
>2043	>comp20_c0_seq1_3	YAVPEGTLR

>2044	>MMa15121	SFDYALTCSR
>2045	>MMa15121	VMDDFVDLSCNK
>2046	>MMa15121	YSDCLTTNIDVK
>2047	>comp16_c0_seq1_5	YYCTILGENEYCR
>2048	>comp53_c0_seq1_4	CNSWNDNLITK
>2049	>comp53_c0_seq1_4	ISCIPSEHDDLCDQFCK
>2050	>comp53_c0_seq1_4	ISCIPSEHDDLCDQFCKK
>2051	>comp21_c0_seq1_5	LHLASGGSCQQPAPFVK
>2052	>comp105006_c0_seq1_4	LATVISPR
>2053	>MMa04555	VAECLFNNYCNNECTK
>2054	>MMa02192	VSCLWGNEGCNK
>2055	>MMa02192	VSCLWGNEGCNKECR
>2056	>MMa04555	VAECLFNNYCNNECTK
>2057	>comp201_c0_seq1_3	DGYCVGDASICFCK
>2058	>comp105006_c0_seq1_4	LATVISPR
>2059	>MMa13619	LCTDNGAESGYCQWGGR
>2060	>comp21_c0_seq1_5	LHLASGGSCQQPAPFVK
>2061	>comp144019_c0_seq1_5	NSSPGDKITK
>2062	>comp105849_c0_seq1_6	GLFIIDDKGILR
>2063	>comp16_c0_seq1_5	LHGVTYGYCYNSR
>2064	>comp16_c0_seq1_5	YYCTILGENEYCR
>2065	>comp16_c0_seq1_5	YYCTILGENEYCRK
>2066	>comp20_c0_seq1_3	YAVPEGTLR
>2067	>MMa17865	VWCVINNESCNSECK
>2068	>MMa17864	SELWAYETNK
>2069	>comp58234_c0_seq1_4	IVLSAGTIINMR
>2070	>MMa15121	AKYSDCLTTNIDVK
>2071	>MMa15121	EYKSFDYALTCSR
>2072	>MMa15121	NAVSEFQLHEILK
>2073	>MMa15121	NAVSEFQLHEILKK
>2074	>MMa15121	SFDYALTCSR
>2075	>MMa15121	VMDDFVDLSCNK
>2076	>MMa15121	VMDDFVDLSCNKLHPSSDEK
>2077	>MMa15121	YSDCLTTNIDVK
>2078	>comp129172_c0_seq1_4	FQIYSKICLNK
>2079	>comp16_c0_seq1_5	LEDKDVTIWNAVK
>2080	>comp16_c0_seq1_5	LHGVTYGYCYNSR
>2081	>comp16_c0_seq1_5	YYCTILGENEYCR
>2082	>comp16_c0_seq1_5	YYCTILGENEYCRK
>2083	>comp20_c0_seq1_3	YAVPEGTLR
>2084	>comp105006_c0_seq1_4	LATVISPR
>2085	>MMa15121	VMDDFVDLSCNK

>2086	>MMa15121	YSDCLTTNIDVK
>2087	>MMa17865	VWCVINNESCNSECK
>2088	>MMa17864	IWCVINNESCNSECK
>2089	>MMa17864	SELWAYETNK
>2090	>comp106427_c0_seq1_6	VATVPSIR
>2091	>comp20_c0_seq1_3	YAVPEGTLR
>2092	>comp16_c0_seq1_5	YYCTILGENEYCR
>2093	>comp16_c0_seq1_5	YYCTILGENEYCRK
>2094	>comp21_c0_seq1_5	LHLASGGSCQQPAPFVK
>2095	>comp105006_c0_seq1_4	LATVISPR
>2096	>MMa02192	VSCLWGNEGCNKECR
>2097	>MMa13616	LPDSVPIR
>2098	>MMa17864	SELWAYETNK
>2099	>MMa20191	YGNACWCIDLPDKVPIR
>2100	>comp105006_c0_seq1_4	LATVISPR
>2101	>MMa13619	LCTDNGAESGYCQWGGR
>2102	>MMa02192	VSCLWGNEGCNK
>2103	>MMa02192	VSCLWGNEGCNKECR
>2104	>MMa13616	LCTENGAESGYCQWGGK
>2105	>MMa13616	LPDSVPIR
>2106	>MMa17864	IWCVINNESCNSECK
>2107	>comp7_c0_seq4_4	LCTDNGAESGYCQWGGK
>2108	>comp105006_c0_seq1_4	LATVISPR
>2109	>comp2175_c0_seq2_3	DVLSIPSSEK
>2110	>MMa13619	LCTDNGAESGYCQWGGR
>2111	>MMa02192	VSCLWGNEGCNK
>2112	>MMa02192	VSCLWGNEGCNKECR
>2113	>MMa13616	DAYIAKPENCVYECGITQDCNK
>2114	>MMa13616	LPDSVPIR
>2115	>MMa13616	YGNACWCIK
>2116	>MMa20191	YGNACWCIDLPDKVPIR
>2117	>MMa17864	IWCVINNESCNSECK
>2118	>comp7_c0_seq4_4	DAYIAKPENCVYHCATNEGCNK
>2119	>comp7_c0_seq4_4	YGNACWCIK
>2120	>comp514_c1_seq77_5	CVLSLGNFLISK
>2121	>comp105006_c0_seq1_4	LATVISPR
>2122	>MMa34629	LPDKVPIR
>2123	>comp34_c2_seq1_2	LACWCDDIHNWVPTWSR
>2124	>MMa13619	DAYIAKPENCVYHCATNEGCNK
>2125	>MMa13619	LCTDNGAESGYCQWGGR
>2126	>MMa13619	YGNACWCIK
>2127	>MMa02192	GSNGCKVSCLWGNEGCNK

>2128	>MMa02192	VSCLWGNEGCNK
>2129	>MMa02192	VSCLWGNEGCNKECR
>2130	>MMa13616	DAYIAKPENCVYECGITQDCNK
>2131	>MMa13616	LPDSVPIR
>2132	>MMa13616	LPDSVPIRVPGK
>2133	>MMa17864	IWCVINNESCNSECK
>2134	>MMa17864	SELWAYETNK
>2135	>MMa55372	YGNACWCYKLPDDAR
>2136	>comp7_c0_seq4_4	DAYIAKPENCVYHCATNEGCNK
>2137	>MMa35530	LTSMSEYACPVIEK
>2138	>comp105006_c0_seq1_4	LATVISPR
>2139	>MMa34629	LPDKVPIR
>2140	>MMa13619	DAYIAKPENCVYHCATNEGCNK
>2141	>MMa13619	LCTDNGAESGYCQWGGR
>2142	>MMa02192	VSCLWGNEGCNK
>2143	>MMa02192	VSCLWGNEGCNKECR
>2144	>MMa13616	LCTENGAESGYCQWGGK
>2145	>MMa13616	LPDSVPIR
>2146	>comp106427_c0_seq1_6	VATVPSIR
>2147	>comp7_c0_seq4_4	LCTDNGAESGYCQWGGK
>2148	>comp173778_c0_seq1_6	ISCLWFQTRIGG
>2149	>comp10876_c0_seq1_2	LSSPASIDKK
>2150	>comp514_c1_seq77_5	CVLSLGNFLISK
>2151	>MMa20191	YGNACWCIDLPDK
>2152	>MMa55199	THINIVVIGHVDSGK
>2153	>comp21_c0_seq1_5	LHLASGGSCQQPAPFVK
>2154	>comp105006_c0_seq1_4	LATVISPR
>2155	>comp140369_c0_seq1_5	VRSSPSVK
>2156	>MMa04555	VAECLFNNYCNNECTK
>2157	>MMa13619	LCTDNGAESGYCQWGGR
>2158	>MMa02192	VSCLWGNEGCNK
>2159	>MMa02192	VSCLWGNEGCNKECR
>2160	>MMa02192	VSCLWGNEGCNK
>2161	>MMa02192	VSCLWGNEGCNKECR
>2162	>MMa20191	YGNACWCIDLPDKVPIR
>2163	>MMa04555	VYYADKGYCCLLK
>2164	>comp105006_c0_seq1_4	LATVISPR
>2165	>MMa55199	THINIVVIGHVDSGK
>2166	>MMa13616	LPDSVPIR
>2167	>MMa04555	VAECLFNNYCNNECTK
>2168	>MMa04555	VYYADKGYCCLLK
>2169	>MMa55199	THINIVVIGHVDSGK

>2170	>comp514_c1_seq77_5	CVLSLGNFLISK
>2171	>MMa20191	YGNACWCIDLPDKVPIR
>2172	>comp48577_c0_seq2_3	NITFMNGSVIGFK
>2173	>comp105006_c0_seq1_4	LATVISPR
>2174	>comp34_c2_seq1_2	LACWCDDIHNWVPTWSR
>2175	>MMa02192	VSCLWGNEGCNK
>2176	>MMa02192	VSCLWGNEGCNKECR
>2177	>MMa20191	YGNACWCIDLPDKVPIR
>2178	>MMa02192	VSCLWGNEGCNK
>2179	>MMa02192	VSCLWGNEGCNKECR
>2180	>comp97316_c0_seq1_5	MIILGDIE
>2181	>comp105006_c0_seq1_4	LATVISPR
>2182	>comp33184_c0_seq4_1	IMGDIINK
>2183	>MMa20191	YGNACWCIDLPDKVPIR
>2184	>MMa02192	VSCLWGNEGCNK
>2185	>MMa02192	VSCLWGNEGCNKECR
>2186	>comp105006_c0_seq1_4	LATVISPR
>2187	>MMa13616	LPDSVPIR
>2188	>MMa20191	YGNACWCIDLPDKVPIR
>2189	>MMa02192	VSCLWGNEGCNKECR
>2190	>comp105006_c0_seq1_4	LATVISPR
>2191	>MMa20191	YGNACWCIDLPDKVPIR
>2192	>MMa02192	VSCLWGNEGCNK
>2193	>MMa02192	VSCLWGNEGCNKECR
>2194	>MMa13616	DAYIAKPENCVYECGITQDCNK
>2195	>MMa17865	GGYYGYCYFWK
>2196	>MMa17865	KSELWNYNTNK
>2197	>MMa17865	VWCVINNESCNSECKIR
>2198	>MMa17864	IWCVINNESCNSECK
>2199	>MMa17864	IWCVINNESCNSECKLR
>2200	>MMa55372	AESGYCQWASK
>2201	>MMa55372	YGNACWCYKLPDDAR
>2202	>MMa35530	LTSMSEYACPVIEK
>2203	>MMa20191	YGNACWCIDLPDKVPIR
>2204	>comp105006_c0_seq1_4	LATVISPR
>2205	>MMa34629	DGYIADDKNCAYFCGR
>2206	>MMa34629	LPDKVPIR
>2207	>MMa13619	LCTDNGAESGYCQWGGR
>2208	>MMa02192	VSCLWGNEGCNK
>2209	>MMa02192	VSCLWGNEGCNKECR
>2210	>comp6939_c0_seq1_1	TCLLGGGSR
>2211	>MMa02192	VSCLWGNEGCNKECR

>2212	>comp33184_c0_seq4_1	IMGDIINK
>2213	>MMa02192	VSCLWGNEGCNK
>2214	>MMa02192	VSCLWGNEGCNKECR
>2215	>comp105006_c0_seq1_4	LATVISPR
>2216	>MMa34629	LPDKVPIR
>2217	>MMa35530	LTSMSEYACPVIEK
>2218	>MMa13616	LPDSVPIR
>2219	>comp141_c0_seq1_4	GFLLGFCDQR
>2220	>MMa02192	VSCLWGNEGCNK
>2221	>MMa02192	VSCLWGNEGCNKECR
>2222	>comp20_c0_seq1_3	YAVPEGTLR
>2223	>MMa20191	YGNACWCIDLPDKVPIR
>2224	>MMa17864	SELWAYETNK
>2225	>comp7_c0_seq5_6	NEYCNDLCTK
>2226	>comp105006_c0_seq1_4	LATVISPR
>2227	>MMa34629	LPDKVPIR
>2228	>MMa13619	LCTDNGAESGYCQWGGR
>2229	>MMa13616	DAYIAKPENCVYECGITQDCNK
>2230	>MMa13616	LCTENGAESGYCQWGGK
>2231	>comp404_c0_seq1_2	TCESFIYGGVGGNK
>2232	>comp105006_c0_seq1_4	LATVISPR
>2233	>comp7_c0_seq4_4	LCTDNGAESGYCQWGGK
>2234	>MMa02192	VSCLWGNEGCNK
>2235	>MMa02192	VSCLWGNEGCNKECR
>2236	>MMa55372	YGNACWCYK
>2237	>MMa55372	YGNACWCYKLPDDAR
>2238	>comp20_c0_seq1_3	YAVPEGTLR
>2239	>MMa20191	YGNACWCIDLPDK
>2240	>MMa20191	YGNACWCIDLPDKVPIR
>2241	>MMa17864	IWCVINNESCNSECK
>2242	>MMa34629	LPDKVPIR
>2243	>comp141_c0_seq1_4	GFLLGFCDQR
>2244	>MMa35530	LTSMSEYACPVIEK
>2245	>MMa13619	LCTDNGAESGYCQWGGR
>2246	>MMa13616	DAYIAKPENCVYECGITQDCNK
>2247	>MMa13616	LCTENGAESGYCQWGGK
>2248	>comp141_c0_seq1_4	GFLLGFCDQR
>2249	>MMa17864	GNYGYCYFWK
>2250	>MMa17864	IWCVINNESCNSECK
>2251	>comp111960_c0_seq1_5	MALFLEYIR
>2252	>MMa55372	YGNACWCYK
>2253	>MMa55372	YGNACWCYKLPDDAR

>2254	>comp7_c0_seq4_4	LCTDNGAESGYCQWGGK
>2255	>MMa35530	LTSMSEYACPVIEK
>2256	>comp45246_c0_seq1_4	VASISPLR
>2257	>MMa20191	YGNACWCIDLPDK
>2258	>MMa20191	YGNACWCIDLPDKVPIR
>2259	>comp105006_c0_seq1_4	LATVISPR
>2260	>comp404_c0_seq1_2	TCESFIYGGVGGNK
>2261	>MMa34629	LPDKVPIR
>2262	>comp1465_c0_seq1_3	KGCSIINK
>2263	>MMa13619	LCTDNGAESGYCQWGGR
>2264	>MMa02192	VSCLWGNEGCNK
>2265	>MMa02192	VSCLWGNEGCNKECR
>2266	>MMa12627	DLPDNVPIR
>2267	>MMa12627	DLPDNVPIRVPGK
>2268	>comp38_c0_seq1_5	VSECLLNNYCNNICTK
>2269	>comp105006_c0_seq1_4	LATVISPR
>2270	>comp34_c1_seq4_6	YGNACWCINLPDDKPIR
>2271	>comp7_c0_seq5_6	DAYIAKPHNCVYECAR
>2272	>comp7_c0_seq5_6	IGYCNIQGK
>2273	>comp3855_c0_seq4_5	MIQVSYTR
>2274	>MMa02192	VSCLWGNEGCNKECR
>2275	>comp11842_c0_seq20_3	GGLKDVMR
>2276	>MMa20191	YGNACWCIDLPDKVPIR
>2277	>MMa02192	VSCLWGNEGCNK
>2278	>MMa37864	HLGGWSGYCYAFNCK
>2279	>comp16_c0_seq1_5	LEDKDVTIWNAVK
>2280	>comp16_c0_seq1_5	LHGVTYGYCYNSR
>2281	>comp16_c0_seq1_5	YYCTILGENEYCRK
>2282	>comp20_c0_seq1_3	YAVPEGTLR
>2283	>comp45246_c0_seq1_4	VASISPLR
>2284	>MMa17865	SELWNYNTNK
>2285	>comp105006_c0_seq1_4	LATVISPR
>2286	>comp20115_c0_seq1_6	NDYLLQSTKHK
>2287	>comp10876_c0_seq1_2	LSSPASIDKK
>2288	>comp106427_c0_seq1_6	VATVPSIR
>2289	>comp20_c0_seq1_3	YAVPEGTLR
>2290	>comp26_c0_seq1_5	LCPSSTAICHR
>2291	>comp105006_c0_seq1_4	LATVISPR
>2292	>MMa28760	YSFTYALVR
>2293	>MMa35530	LTSMSEYACPVIEK
>2294	>MMa35530	HSWNKLTSMSEYACPVIEK
>2295	>MMa35530	LTSMSEYACPVIEK

>2296	>MMa17865	KSELWNYNTNK
>2297	>MMa17865	VWCVINNESCNSECKIR
>2298	>comp105006_c0_seq1_4	LATVISPR
>2299	>comp20_c0_seq1_3	TQFGCPAYQGYCDDHCQDIKK
>2300	>comp20_c0_seq1_3	YAVPEGTLR
>2301	>comp105006_c0_seq1_4	LATVISPR
>2302	>comp20_c0_seq1_3	YAVPEGTLR
>2303	>comp26_c0_seq1_5	LCPSSTAICHR
>2304	>comp5130_c0_seq2_3	TPSVSLPR
>2305	>comp105006_c0_seq1_4	LATVISPR
>2306	>comp156076_c0_seq1_3	EDTTEVCRR
>2307	>MMa29185	IYCSLTNMK
>2308	>MMa29185	TLDEMTDFYCNK
>2309	>comp404_c0_seq1_2	TCESFIYGGVGGNK
>2310	>MMa20191	YGNACWCIDLPDKVPIR
>2311	>comp20_c0_seq1_3	YAVPEGTLR
>2312	>MMa17865	GGYYGYCYFWK
>2313	>MMa17865	KSELWNYNTNK
>2314	>MMa17865	VWCVINNESCNSECK
>2315	>MMa17864	IWCVINNESCNSECK
>2316	>comp105006_c0_seq1_4	LATVISPR
>2317	>comp105006_c0_seq1_4	LATVISPR
>2318	>comp66_c0_seq1_4	LATKIIPSLFR
>2319	>comp105006_c0_seq1_4	LATVISPR
>2320	>comp105006_c0_seq1_4	LATVISPR
>2321	>comp106427_c0_seq1_6	VATVPSIR
>2322	>comp20_c0_seq1_3	TIIQTAVHKLGK
>2323	>comp20_c0_seq1_3	YAVPEGTLR
>2324	>comp11643_c0_seq1_1	GNRGSLPR
>2325	>comp20_c0_seq1_3	YAVPEGTLR
>2326	>MMa17865	GGYYGYCYFWK
>2327	>MMa17864	SELWAYETNK
>2328	>MMa35530	LTSMSEYACPVIEK
>2329	>comp20_c0_seq1_3	YAVPEGTLR
>2330	>comp404_c0_seq1_2	NNFLNIENCCK
>2331	>comp404_c0_seq1_2	TCESFIYGGVGGNK
>2332	>MMa34629	LPDKVPIR
>2333	>MMa17865	GGYYGYCYFWK
>2334	>MMa17865	KSELWNYNTNK
>2335	>MMa17865	SELWNYNTNK
>2336	>MMa17865	VWCVINNESCNSECK
>2337	>MMa17865	VWCVINNESCNSECKIR

>2338	>MMa17864	IWCVINNESCNSECK
>2339	>MMa55372	YGNACWCYKLPDDAR
>2340	>comp23761_c0_seq3_4	LPSDVQVGSMTK
>2341	>MMa35530	LTSMSEYACPVIEK
>2342	>comp20_c0_seq1_3	YAVPEGTLR
>2343	>comp84321_c0_seq1_1	RGGSVFIR
>2344	>comp5568_c0_seq1_6	KCSDGALNISNR
>2345	>comp105006_c0_seq1_4	LATVISPR
>2346	>comp404_c0_seq1_2	DCSLPVDTGR
>2347	>comp404_c0_seq1_2	NNFLNIENCCK
>2348	>comp404_c0_seq1_2	NSKTCESFIYGGVGGNK
>2349	>comp404_c0_seq1_2	TCESFIYGGVGGNK
>2350	>comp404_c0_seq1_2	TCESFIYGGVGGNKNNFLNIENCCK
>2351	>MMa04555	VAECLFNNYCNNECTK
>2352	>MMa02192	VSCLWGNEGCNK
>2353	>MMa17865	GGYYGYCYFWK
>2354	>MMa17865	KSELWNYNTNK
>2355	>MMa17865	SELWNYNTNK
>2356	>MMa17865	VWCVINNESCNSECK
>2357	>MMa17865	VWCVINNESCNSECKIR
>2358	>MMa17864	IWCVINNESCNSECK
>2359	>MMa17864	IWCVINNESCNSECKLR
>2360	>MMa55372	YGNACWCYKLPDDAR
>2361	>MMa35530	HSWNKLTSMSEYACPVIEK
>2362	>MMa35530	LTSMSEYACPVIEK
>2363	>comp20_c0_seq1_3	TIIQTAVHKLGK
>2364	>comp162_c0_seq1_6	HGYCMNYDCFCTALRDEIK
>2365	>comp105006_c0_seq1_4	LATVISPR
>2366	>comp404_c0_seq1_2	DCSLPVDTGR
>2367	>comp404_c0_seq1_2	NNFLNIENCCK
>2368	>comp404_c0_seq1_2	NSKTCESFIYGGVGGNK
>2369	>comp404_c0_seq1_2	TCESFIYGGVGGNK
>2370	>comp404_c0_seq1_2	TCESFIYGGVGGNKNNFLNIENCCK
>2371	>MMa12627	DLPDNVPIRVPGK
>2372	>comp38_c0_seq1_5	VSECLLNNYCNNICTK
>2373	>comp105006_c0_seq1_4	LATVISPR
>2374	>comp810_c0_seq1_5	NLKMMVVLIIMVIK
>2375	>comp74_c0_seq1_1	DAYCNELCTK
>2376	>MMa12627	DAYCNELCTK
>2377	>MMa12627	DLPDNVPIRVPGK
>2378	>comp38_c0_seq1_5	VSECLLNNYCNNICTK
>2379	>comp105006_c0_seq1_4	LATVISPR

>2380	>MMa20191	YGNACWCIDLPDK
>2381	>MMa20191	YGNACWCIDLPDKVPIR
>2382	>comp38_c0_seq1_5	VSECLLNNYCNNICTK
>2383	>comp105006_c0_seq1_4	LATVISPR
>2384	>comp105006_c0_seq1_4	LATVISPR
>2385	>MMa17865	GGYYGYCYFWK
>2386	>MMa17865	KSELWNYNTNK
>2387	>MMa17865	LACFCQGAR
>2388	>MMa17865	SELWNYNTNK
>2389	>MMa17865	VWCVINNESCNSECK
>2390	>MMa17865	VWCVINNESCNSECKIR
>2391	>MMa17865	YTGCKVWCVINNESCNSECK
>2392	>MMa17864	IWCVINNESCNSECK
>2393	>MMa17864	IWCVINNESCNSECKLR
>2394	>MMa17864	SELWAYETNK
>2395	>MMa17864	YTGCKIWCVINNESCNSECK
>2396	>MMa55372	AESGYCQWASK
>2397	>MMa55372	YGNACWCYKLPDDAR
>2398	>MMa04555	VAECLFNNYCNNECTK
>2399	>MMa35530	HSWNKLTSMSEYACPVIEK
>2400	>MMa35530	LTSMSEYACPVIEK
>2401	>MMa20191	YGNACWCIDLPDKVPIR
>2402	>comp20_c0_seq1_3	YAVPEGTLR
>2403	>comp162_c0_seq1_6	HGYCMNYDCFCTALRDEIK
>2404	>comp105006_c0_seq1_4	LATVISPR
>2405	>comp404_c0_seq1_2	DCSLPVDTGR
>2406	>comp404_c0_seq1_2	NNFLNIENCCK
>2407	>comp404_c0_seq1_2	TCESFIYGGVGGNK
>2408	>comp404_c0_seq1_2	TCESFIYGGVGGNKNNFLNIENCCK
>2409	>MMa13619	LCTDNGAESGYCQWGGR
>2410	>MMa17865	GGYYGYCYFWK
>2411	>MMa17865	KSELWNYNTNK
>2412	>MMa17865	LACFCQGAR
>2413	>MMa17865	SELWNYNTNK
>2414	>MMa17865	VWCVINNESCNSECK
>2415	>MMa17865	VWCVINNESCNSECKIR
>2416	>MMa17865	YTGCKVWCVINNESCNSECK
>2417	>MMa17864	GNYGYCYFWK
>2418	>MMa17864	IWCVINNESCNSECK
>2419	>MMa17864	IWCVINNESCNSECKLR
>2420	>MMa17864	SELWAYETNK
>2421	>MMa17864	YTGCKIWCVINNESCNSECK

>2422	>MMa55372	AESGYCQWASK
>2423	>MMa55372	DGYIADDRNCPYFCGR
>2424	>MMa55372	YGNACWCYK
>2425	>MMa55372	YGNACWCYKLPDDAR
>2426	>comp38_c0_seq1_5	VSECLLNNYCNNICTK
>2427	>MMa35530	HSWNKLTSMSEYACPVIEK
>2428	>MMa35530	LTSMSEYACPVIEK
>2429	>comp20_c0_seq1_3	TIIQTAVHKLGK
>2430	>comp20_c0_seq1_3	YAVPEGTLR
>2431	>comp105006_c0_seq1_4	LATVISPR
>2432	>comp404_c0_seq1_2	DCSLPVDTGR
>2433	>comp404_c0_seq1_2	NNFLNIENCCK
>2434	>comp404_c0_seq1_2	TCESFIYGGVGGNK
>2435	>MMa34629	DGYIADDKNCAYFCGR
>2436	>comp18681_c0_seq4_5	VPLCNKVILLR
>2437	>MMa13619	LCTDNGAESGYCQWGGR
>2438	>MMa02192	VSCLWGNEGCNK
>2439	>MMa17865	GGYYGYCYFWK
>2440	>MMa17865	KSELWNYNTNK
>2441	>MMa17865	LACFCQGAR
>2442	>MMa17865	VWCVINNESCNSECK
>2443	>MMa17865	VWCVINNESCNSECKIR
>2444	>MMa17865	YTGCKVWCVINNESCNSECK
>2445	>MMa17864	IWCVINNESCNSECK
>2446	>MMa17864	IWCVINNESCNSECKLR
>2447	>MMa17864	LACYCEGAPKSELWAYETNK
>2448	>MMa17864	SELWAYETNK
>2449	>MMa55372	YGNACWCYK
>2450	>MMa55372	YGNACWCYKLPDDAR
>2451	>MMa35530	LTSMSEYACPVIEK
>2452	>MMa20191	YGNACWCIDLPDKVPIR
>2453	>comp7_c0_seq4_4	DAYIAKPENCVYHCATNEGCNK
>2454	>comp20_c0_seq1_3	TIIQTAVHKLGK
>2455	>comp20_c0_seq1_3	YAVPEGTLR
>2456	>comp16_c0_seq1_5	LHGVTYGYCYNSR
>2457	>comp16_c0_seq1_5	YYCTILGENEYCR
>2458	>comp105006_c0_seq1_4	LATVISPR
>2459	>comp404_c0_seq1_2	TCESFIYGGVGGNK
>2460	>comp404_c0_seq1_2	TCESFIYGGVGGNKNNFLNIENCCK
>2461	>MMa34629	DGYIADDKNCAYFCGR
>2462	>MMa13619	DAYIAKPENCVYHCATNEGCNK
>2463	>MMa13619	LCTDNGAESGYCQWGGR

>2464	>MMa13619	LCTDNGAESGYCQWGGR
>2465	>MMa17865	KSELWNYNTNK
>2466	>MMa17865	VWCVINNESCNSECK
>2467	>MMa17864	IWCVINNESCNSECK
>2468	>MMa17864	IWCVINNESCNSECKLR
>2469	>MMa17864	LACYCEGAPKSELWAYETNK
>2470	>MMa17864	SELWAYETNK
>2471	>MMa55372	AESGYCQWASK
>2472	>MMa55372	DGYIADDRNCPYFCGR
>2473	>MMa55372	NRAESGYCQWASK
>2474	>MMa55372	YGNACWCYK
>2475	>MMa55372	YGNACWCYKLPDDAR
>2476	>MMa04555	VAECLFNNYCNNECTK
>2477	>MMa35530	LTSMSEYACPVIEK
>2478	>MMa35530	LTSMSEYACPVIEKWCEDHCAAK
>2479	>MMa20191	YGNACWCIDLPDKVPIR
>2480	>comp105006_c0_seq1_4	LATVISPR
>2481	>comp404_c0_seq1_2	DCSLPVDTGR
>2482	>comp404_c0_seq1_2	NNFLNIENCCK
>2483	>comp404_c0_seq1_2	NSKTCESFIYGGVGGNK
>2484	>comp404_c0_seq1_2	TCESFIYGGVGGNK
>2485	>comp404_c0_seq1_2	TCESFIYGGVGGNKNNFLNIENCCK
>2486	>MMa34629	DGYIADDKNCAYFCGR
>2487	>MMa34629	LPDKVPIR
>2488	>comp34_c2_seq1_2	LACWCDDIHNWVPTWSR
>2489	>MMa02192	VSCLWGNEGCNK
>2490	>MMa02192	VSCLWGNEGCNKECR
>2491	>MMa17865	GGYYGYCYFWK
>2492	>MMa17865	KSELWNYNTNK
>2493	>MMa17865	LACFCQGAR
>2494	>MMa17865	SELWNYNTNK
>2495	>MMa17865	VWCVINNESCNSECK
>2496	>comp38_c0_seq1_5	VSECLLNNYCNNICTK
>2497	>MMa55372	AESGYCQWASK
>2498	>MMa55372	DGYIADDRNCPYFCGR
>2499	>MMa55372	NRAESGYCQWASK
>2500	>MMa55372	YGNACWCYK
>2501	>MMa55372	YGNACWCYKLPDDAR
>2502	>MMa17864	IWCVINNESCNSECK
>2503	>MMa35530	HSWNKLTSMSEYACPVIEK
>2504	>MMa35530	LTSMSEYACPVIEK
>2505	>MMa35530	LTSMSEYACPVIEKWCEDHCAAK

>2506	>MMa20191	YGNACWCIDLPDKVPIR
>2507	>comp105006_c0_seq1_4	LATVISPR
>2508	>comp404_c0_seq1_2	NNFLNIENCCK
>2509	>comp404_c0_seq1_2	TCESFIYGGVGGNK
>2510	>comp404_c0_seq1_2	TCESFIYGGVGGNKNNFLNIENCCK
>2511	>MMa34629	DGYIADDKNCAYFCGR
>2512	>comp34_c2_seq1_2	LACWCDDIHNWVPTWSR
>2513	>MMa13619	LCTDNGAESGYCQWGGR
>2514	>MMa02192	VSCLWGNEGCNK
>2515	>MMa02192	VSCLWGNEGCNKECR
>2516	>comp7_c0_seq4_4	DAYIAKPENCVYHCATNEGCNK
>2517	>comp7_c0_seq4_4	YGNACWCIK
>2518	>comp404_c0_seq1_2	NNFLNIENCCK
>2519	>comp404_c0_seq1_2	TCESFIYGGVGGNK
>2520	>comp404_c0_seq1_2	TCESFIYGGVGGNKNNFLNIENCCK
>2521	>MMa55372	AESGYCQWASK
>2522	>MMa55372	DGYIADDRNCPYFCGR
>2523	>MMa55372	NRAESGYCQWASK
>2524	>MMa55372	YGNACWCYKLPDDAR
>2525	>MMa02192	VSCLWGNEGCNKECR
>2526	>comp20_c0_seq1_3	TIIQTAVHKLGK
>2527	>comp20_c0_seq1_3	YAVPEGTLR
>2528	>MMa20191	YGNACWCIDLPDKVPIR
>2529	>MMa17865	GGYYGYCYFWK
>2530	>MMa17865	IRGGYYGYCYFWK
>2531	>MMa17865	KSELWNYNTNK
>2532	>MMa17865	LACFCQGAR
>2533	>MMa17865	SELWNYNTNK
>2534	>MMa17865	VWCVINNESCNSECK
>2535	>MMa17865	VWCVINNESCNSECKIR
>2536	>MMa17865	YTGCKVWCVINNESCNSECK
>2537	>MMa17864	GNYGYCYFWK
>2538	>MMa17864	IWCVINNESCNSECK
>2539	>MMa17864	IWCVINNESCNSECKLR
>2540	>MMa17864	LACYCEGAPK
>2541	>MMa17864	LACYCEGAPKSELWAYETNK
>2542	>MMa17864	SELWAYETNK
>2543	>MMa17864	SELWAYETNKCNGR
>2544	>MMa17864	YTGCKIWCVINNESCNSECK
>2545	>MMa34629	DGYIADDKNCAYFCGR
>2546	>MMa34629	LPDKVPIR
>2547	>MMa13616	YGNACWCIK

>2548	>comp34_c2_seq1_2	LACWCDDIHNWVPTWSR
>2549	>MMa35530	LTSMSEYACPVIEK
>2550	>MMa35530	LTSMSEYACPVIEKWCEDHCAAK
>2551	>MMa13619	DAYIAKPENCVYHCATNEGCNK
>2552	>MMa13619	LCTDNGAESGYCQWGGR
>2553	>MMa13619	YGNACWCIK
>2554	>MMa13619	YGNACWCIKLPDR
>2555	>MMa17865	KSELWNYNTNK
>2556	>MMa17865	LACFCQGARK
>2557	>MMa17865	SELWNYNTNK
>2558	>MMa17865	VWCVINNESCNSECK
>2559	>MMa17865	VWCVINNESCNSECKIR
>2560	>MMa17864	GNYGYCYFWKLACYCEGAPK
>2561	>MMa17864	IWCVINNESCNSECKLR
>2562	>MMa17864	SELWAYETNKCNGR
>2563	>MMa55372	AESGYCQWASK
>2564	>MMa55372	NRAESGYCQWASK
>2565	>MMa55372	YGNACWCYK
>2566	>MMa55372	YGNACWCYKLPDDAR
>2567	>comp7_c0_seq4_4	DAYIAKPENCVYHCATNEGCNK
>2568	>comp38_c0_seq1_5	VSECLLNNYCNNICTK
>2569	>MMa35530	LTSMSEYACPVIEK
>2570	>MMa20191	YGNACWCIDLPDKVPIR
>2571	>comp20_c0_seq1_3	KEEGFCHGFK
>2572	>comp20_c0_seq1_3	TIIQTAVHK
>2573	>comp20_c0_seq1_3	YAVPEGTLR
>2574	>comp162_c0_seq1_6	IKCSILGTNNK
>2575	>comp105006_c0_seq1_4	LATVISPR
>2576	>comp404_c0_seq1_2	DCSLPVDTGR
>2577	>MMa34629	DGYIADDKNCAYFCGR
>2578	>MMa34629	LPDKVPIR
>2579	>MMa34629	NGAESGYCQWAGVYGNACWCYK
>2580	>comp34_c2_seq1_2	LACWCDDIHNWVPTWSR
>2581	>MMa13619	DAYIAKPENCVYHCATNEGCNK
>2582	>MMa13619	LCTDNGAESGYCQWGGR
>2583	>MMa13619	YGNACWCIKLPDR
>2584	>MMa02192	VSCLWGNEGCNK
>2585	>MMa02192	VSCLWGNEGCNKECR
>2586	>MMa13616	YGNACWCIK
>2587	>MMa17865	KSELWNYNTNK
>2588	>MMa17865	SELWNYNTNK
>2589	>MMa17865	VWCVINNESCNSECK

>2590	>MMa17864	GNYGYCYFWK
>2591	>MMa17864	IWCVINNESCNSECK
>2592	>MMa17864	IWCVINNESCNSECKLR
>2593	>MMa17864	SELWAYETNKCNGR
>2594	>MMa55372	AESGYCQWASK
>2595	>MMa55372	DGYIADDRNCPYFCGR
>2596	>MMa55372	NRAESGYCQWASK
>2597	>MMa55372	YGNACWCYK
>2598	>MMa55372	YGNACWCYKLPDDAR
>2599	>comp7_c0_seq4_4	DAYIAKPENCVYHCATNEGCNK
>2600	>comp7_c0_seq4_4	LCTDNGAESGYCQWGGK
>2601	>comp7_c0_seq4_4	YGNACWCIK
>2602	>comp38_c0_seq1_5	VSECLLNNYCNNICTK
>2603	>MMa35530	LTSMSEYACPVIEK
>2604	>MMa35530	LTSMSEYACPVIEKWCEDHCAAK
>2605	>comp10876_c0_seq1_2	LSSPASIDKK
>2606	>comp141_c0_seq1_4	GFLLGFCDQR
>2607	>comp141_c0_seq1_4	KGFLLGFCDQR
>2608	>MMa20191	YGNACWCIDLPDK
>2609	>MMa20191	YGNACWCIDLPDKVPIR
>2610	>comp20_c0_seq1_3	YAVPEGTLR
>2611	>comp7_c0_seq8_6	CITIEGCNNFCTK
>2612	>comp105006_c0_seq1_4	LATVISPR
>2613	>MMa12627	DLPDNVPIRVPGK
>2614	>comp54_c0_seq1_5	GTCHDFNDIGCK
>2615	>comp404_c0_seq1_2	TCESFIYGGVGGNK
>2616	>MMa34629	LPDKVPIR
>2617	>comp34_c2_seq1_2	LACWCDDIHNWVPTWSR
>2618	>MMa13619	DAYIAKPENCVYHCATNEGCNK
>2619	>MMa13619	LCTDNGAESGYCQWGGR
>2620	>MMa13619	YGNACWCIK
>2621	>MMa02192	VSCLWGNEGCNK
>2622	>MMa02192	VSCLWGNEGCNKECR
>2623	>MMa13616	LCTENGAESGYCQWGGK
>2624	>MMa13616	LPDSVPIR
>2625	>MMa13616	LPDSVPIRVPGK
>2626	>MMa13619	LCTDNGAESGYCQWGGR
>2627	>comp7_c0_seq2_6	YGNACWCIELPDNVPIRVPGK
>2628	>MMa17865	KSELWNYNTNK
>2629	>MMa17865	LACFCQGAR
>2630	>MMa17865	SELWNYNTNK
>2631	>MMa17865	VWCVINNESCNSECKIR

>2632	>MMa17864	IWCVINNESCNSECK
>2633	>MMa17864	IWCVINNESCNSECKLR
>2634	>MMa17864	SELWAYETNKCNGR
>2635	>MMa55372	AESGYCQWASK
>2636	>MMa55372	DGYIADDRNCPYFCGR
>2637	>MMa55372	YGNACWCYK
>2638	>MMa55372	YGNACWCYKLPDDAR
>2639	>comp23761_c0_seq3_4	LPSDVQVGSMTK
>2640	>comp7_c0_seq4_4	LCTDNGAESGYCQWGGK
>2641	>comp7_c0_seq5_6	DAYIAKPHNCVYECAR
>2642	>comp7_c0_seq5_6	NEYCNDLCTK
>2643	>comp38_c0_seq1_5	VSECLLNNYCNNICTK
>2644	>MMa35530	HSWNKLTSMSEYACPVIEK
>2645	>MMa35530	LTSMSEYACPVIEK
>2646	>MMa35530	LTSMSEYACPVIEKWCEDHCAAK
>2647	>comp141_c0_seq1_4	GFLLGFCDQR
>2648	>comp141_c0_seq1_4	KGFLLGFCDQR
>2649	>MMa20191	YGNACWCIDLPDKVPIR
>2650	>comp24810_c0_seq1_2	CFCKSVQR
>2651	>comp20_c0_seq1_3	TQFGCPAYQGYCDDHCQDIK
>2652	>comp20_c0_seq1_3	YAVPEGTLR
>2653	>comp7_c0_seq8_6	CITIEGCNNFCTK
>2654	>comp16_c0_seq1_5	LHGVTYGYCYNSR
>2655	>comp34_c2_seq1_2	LACWCDDIHNWVPTWSR
>2656	>comp34_c2_seq1_2	NWCVLNHSCGILCEGYGGSGYCYFWK
>2657	>comp105006_c0_seq1_4	LATVISPR
>2658	>MMa12627	DLPDNVPIRVPGK
>2659	>comp404_c0_seq1_2	TCESFIYGGVGGNK
>2660	>MMa34629	DGYIADDKNCAYFCGR
>2661	>MMa34629	LPDKVPIR
>2662	>MMa34629	NGAESGYCQWAGVYGNACWCYK
>2663	>comp199_c0_seq1_5	ELCLLPHK
>2664	>MMa02192	VSCLWGNEGCNK
>2665	>MMa02192	VSCLWGNEGCNKECR
>2666	>MMa17864	IWCVINNESCNSECK
>2667	>comp7_c0_seq4_4	DAYIAKPENCVYHCATNEGCNK
>2668	>comp7_c0_seq4_4	LCTDNGAESGYCQWGGK
>2669	>comp38_c0_seq1_5	VSECLLNNYCNNICTK
>2670	>MMa20191	YGNACWCIDLPDK
>2671	>MMa20191	YGNACWCIDLPDKVPIR
>2672	>comp105006_c0_seq1_4	LATVISPR
>2673	>MMa34629	LPDKVPIR

>2674	>comp34_c2_seq1_2	LACWCDDIHNWVPTWSR
>2675	>MMa13619	DAYIAKPENCVYHCATNEGCNK
>2676	>MMa13619	LCTDNGAESGYCQWGGR
>2677	>MMa02192	VSCLWGNEGCNK
>2678	>MMa02192	VSCLWGNEGCNKECR
>2679	>comp24134_c0_seq1_2	LASILSPR
>2680	>comp7_c0_seq4_4	LCTDNGAESGYCQWGGK
>2681	>MMa55372	YGNACWCYKLPDDAR
>2682	>MMa02192	VSCLWGNEGCNKECR
>2683	>MMa20191	YGNACWCIDLPDKVPIR
>2684	>MMa17864	IWCVINNESCNSECK
>2685	>MMa17864	SELWAYETNK
>2686	>comp112_c0_seq1_5	TITLEVEPSDTIENVK
>2687	>comp7_c0_seq2_6	YGNACWCIELPDNVPIRVPGK
>2688	>MMa34629	LPDKVPIR
>2689	>comp141_c0_seq1_4	GFLLGFCDQR
>2690	>comp34_c2_seq1_2	LACWCDDIHNWVPTWSR
>2691	>MMa13619	LCTDNGAESGYCQWGGR
>2692	>comp38_c0_seq1_5	VSECLLNNYCNNICTK
>2693	>MMa04118	CFASSECWTACK
>2694	>comp7_c0_seq5_6	DAYIAKPHNCVYECAR
>2695	>comp7_c0_seq5_6	NEYCNDLCTK
>2696	>comp191_c0_seq1_3	CVIEAEGSLIYHLCK
>2697	>comp191_c0_seq1_3	TATFCTQSICQESCK
>2698	>MMa20191	YGNACWCIDLPDK
>2699	>MMa20191	YGNACWCIDLPDKVPIR
>2700	>comp7_c0_seq8_6	CITIEGCNNFCTK
>2701	>MMa12627	DLPDNVPIRVPGK
>2702	>comp105006_c0_seq1_4	LATVISPR
>2703	>comp74_c0_seq1_1	DAYIAQNYNCVYHCAR
>2704	>comp7_c0_seq2_6	YGNACWCIELPDNVPIR
>2705	>comp7_c0_seq8_6	CITIEGCNNFCTK
>2706	>MMa20191	YGNACWCIDLPDKVPIR
>2707	>comp38_c0_seq1_5	VSECLLNNYCNNICTK
>2708	>MMa17865	VWCVINNESCNSECK
>2709	>comp191_c0_seq1_3	CVIEAEGSLIYHLCK
>2710	>comp191_c0_seq1_3	TATFCTQSICQESCK
>2711	>comp105006_c0_seq1_4	LATVISPR
>2712	>comp7_c0_seq5_6	DAYIAKPHNCVYECAR
>2713	>MMa04118	CFASSECWTACK
>2714	>MMa04118	CFASSECWTACKK
>2715	>comp34_c2_seq1_2	LACWCDDIHNWVPTWSR

>2716	>MMa35530	LTSMSEYACPVIEK
>2717	>comp74_c0_seq1_1	DAYCNELCTK
>2718	>MMa12627	DAYCNELCTK
>2719	>MMa12627	DLPDNVPIR
>2720	>MMa12627	DLPDNVPIRVPGK
>2721	>MMa12627	FACYCKDLPDNVPIR
>2722	>MMa20191	YGNACWCIDLPDKVPIR
>2723	>comp38_c0_seq1_5	VSECLLNNYCNNICTK
>2724	>MMa17864	IWCVINNESCNSECK
>2725	>comp191_c0_seq1_3	CVIEAEGSLIYHLCK
>2726	>comp105006_c0_seq1_4	LATVISPR
>2727	>comp7_c0_seq2_6	YGNACWCIELPDNVPIR
>2728	>comp7_c0_seq5_6	DAYIAKPHNCVYECAR
>2729	>comp7_c0_seq5_6	IGYCNIQGK
>2730	>MMa04118	CFASSECWTACK
>2731	>MMa04118	CFASSECWTACKK
>2732	>comp74_c0_seq1_1	DAYIAQNYNCVYHCAR
>2733	>MMa12627	FACYCKDLPDNVPIR
>2734	>MMa20191	YGNACWCIDLPDK
>2735	>MMa20191	YGNACWCIDLPDKVPIR
>2736	>comp7_c0_seq2_6	YGNACWCIELPDNVPIR
>2737	>comp38_c0_seq1_5	VSECLLNNYCNNICTK
>2738	>comp191_c0_seq1_3	CVIEAEGSLIYHLCK
>2739	>comp105006_c0_seq1_4	LATVISPR
>2740	>comp7_c0_seq5_6	DAYIAKPHNCVYECAR
>2741	>comp7_c0_seq5_6	IGYCNIQGK
>2742	>comp105006_c0_seq1_4	LATVISPR
>2743	>MMa12627	DLPDNVPIR
>2744	>comp7_c0_seq2_6	YGNACWCIELPDNVPIR
>2745	>comp34_c1_seq4_6	YGNACWCINLPDDKPIR
>2746	>comp38_c0_seq1_5	VSECLLNNYCNNICTK
>2747	>MMa04118	CFASSECWTACK
>2748	>MMa00982	CFGPQCLCNR
>2749	>comp191_c0_seq1_3	CVIEAEGSLIYHLCK
>2750	>comp74_c0_seq1_1	DAYCNELCTK
>2751	>comp16672_c0_seq10_5	TLLDLDKDK
>2752	>MMa12627	DAYCNELCTK
>2753	>comp105006_c0_seq1_4	LATVISPR
>2754	>comp105006_c0_seq1_4	LATVISPR
>2755	>comp105006_c0_seq1_4	LATVISPR
>2756	>MMa04118	CFASSECWTACK
>2757	>MMa04118	CFASSECWTACKK

>2758	>comp105006_c0_seq1_4	LATVISPR
>2759	>comp16672_c0_seq10_5	TLLDLDKDK
>2760	>comp105006_c0_seq1_4	LATVISPR
>2761	>comp12098_c0_seq1_6	NIEVTIDDK
>2762	>comp105006_c0_seq1_4	LATVISPR
>2763	>comp105006_c0_seq1_4	LATVISPR
>2764	>comp141042_c0_seq1_1	EASPAVDSDR
>2765	>comp105006_c0_seq1_4	LATVISPR
>2766	>comp47462_c0_seq1_4	LNTVLSPR
>2767	>MMa01489	FDPSSEAGAGR
>2768	>MMa01489	FDPTSEAGAGR
>2769	>comp105006_c0_seq1_4	LATVISPR
>2770	>MMa01489	FDPTSEAGAGR
>2771	>MMa01489	FDPASEAGAGR
>2772	>comp105006_c0_seq1_4	LATVISPR
>2773	>MMa01489	FDPASAAGAGR
>2774	>comp12_c2_seq3_1	DCQQYCLTPDR
>2775	>MMa00460	KCPGNPPCR
>2776	>comp12_c2_seq3_1	CSYGTCYCK
>2777	>comp12_c2_seq3_1	DCQQYCLTPDR
>2778	>comp105006_c0_seq1_4	LATVISPR
>2779	>comp105006_c0_seq1_4	LATVISPR
>2780	>comp12_c2_seq3_1	DCQQYCLTPDR
>2781	>MMa01489	FDPTLEAGAGR
>2782	>MMa01489	FDPVSAAGAGR
>2783	>comp105006_c0_seq1_4	LATVISPR
>2784	>comp47462_c0_seq1_4	LNTVLSPR
>2785	>comp12_c2_seq3_1	DCQQYCLTPDR
>2786	>comp150641_c0_seq1_3	LASPLSIFVYHQMWQKGSR
>2787	>MMa00982	CFGPQCLCNR
>2788	>comp20_c0_seq1_3	YAVPEGTLR
>2789	>comp55_c0_seq1_6	CQGGSCASVCR
>2790	>comp105006_c0_seq1_4	LATVISPR
>2791	>comp562_c0_seq1_6	NFDCLKPCR
>2792	>MMa01489	FDPTLEAGAGR
>2793	>MMa04118	CFASSECWTACK
>2794	>comp392_c0_seq1_3	CSASYQCFPVCK
>2795	>comp42_c0_seq1_6	NDIVEEPFK
>2796	>comp191_c0_seq1_3	TATFCTQSICQESCK
>2797	>MMa01489	FNPTSEAGAGR
>2798	>MMa01489	FNPALEAGAGR
>2799	>comp55_c0_seq1_6	CQGGSCASVCR

>2800	>MMa21259	IDAAACYSSDCR
>2801	>comp105006_c0_seq1_4	LATVISPR
>2802	>comp562_c0_seq1_6	NFDCLKPCR
>2803	>comp130124_c0_seq1_5	LLEMQENMVKLVETIFNR
>2804	>MMa04118	CFASSECWTACK
>2805	>MMa00982	CFGPQCLCNR
>2806	>comp7_c0_seq5_6	NEYCNDLCTK
>2807	>comp191_c0_seq1_3	TATFCTQSICQESCK
>2808	>MMa01489	FDPVSAAGAGR
>2809	>MMa43404	YVQSTCGPGTVFNEAR
>2810	>MMa04118	CFASSECWTACK
>2811	>MMa00982	CFGPQCLCNR
>2812	>comp74_c0_seq1_1	DAYCNELCTK
>2813	>MMa12627	DAYCNELCTK
>2814	>MMa12627	DLPDNVPIR
>2815	>comp7_c0_seq5_6	NEYCNDLCTK
>2816	>comp404_c0_seq1_2	DCSLPVDTGR
>2817	>comp404_c0_seq1_2	NNFLNIENCCK
>2818	>comp404_c0_seq1_2	TCESFIYGGVGGNK
>2819	>comp47462_c0_seq1_4	LNTVLSPR
>2820	>MMa04118	CFASSECWTACK
>2821	>MMa55372	AESGYCQWASK
>2822	>MMa55372	YGNACWCYK
>2823	>MMa00982	CFGPQCLCNR
>2824	>comp9340_c1_seq8_6	PATGVPIR
>2825	>comp7_c0_seq5_6	NEYCNDLCTK
>2826	>comp74_c0_seq1_1	DAYCNELCTK
>2827	>comp17079_c0_seq1_4	SSFGLLEGR
>2828	>MMa12627	DAYCNELCTK
>2829	>MMa12627	DLPDNVPIR
>2830	>comp404_c0_seq1_2	DCSLPVDTGR
>2831	>comp404_c0_seq1_2	NNFLNIENCCK
>2832	>comp404_c0_seq1_2	TCESFIYGGVGGNK
>2833	>MMa13616	YGNACWCIK
>2834	>MMa00982	CFGPQCLCNR
>2835	>MMa18140	LPDPVDLR
>2836	>comp7_c0_seq4_4	DAYIAKPENCVYHCATNEGCNK
>2837	>comp7_c0_seq4_4	LCTDNGAESGYCQWGGK
>2838	>comp7_c0_seq4_4	YGNACWCIK
>2839	>MMa20191	YGNACWCIDLPDK
>2840	>MMa20191	YGNACWCIDLPDKVPIR
>2841	>comp7482_c0_seq2_5	MNLEVICDQR

>2842	>comp7_c0_seq5_6	NEYCNDLCTK
>2843	>comp105006_c0_seq1_4	LATVISPR
>2844	>MMa34629	DGYIADDKNCAYFCGR
>2845	>MMa34629	LPDKVPIR
>2846	>comp17568_c0_seq1_6	IGVLKVLPR
>2847	>comp14858_c0_seq1_2	SMQIDIPSR
>2848	>MMa13619	DAYIAKPENCVYHCATNEGCNK
>2849	>MMa13619	LCTDNGAESGYCQWGGR
>2850	>MMa13619	YGNACWCIK
>2851	>MMa13616	DAYIAKPENCVYECGITQDCNK
>2852	>MMa13616	LCTENGAESGYCQWGGK
>2853	>MMa13616	LPDSVPIR
>2854	>MMa13619	LCTDNGAESGYCQWGGR
>2855	>MMa00982	CFGPQCLCNR
>2856	>MMa20191	YGNACWCIDLPDK
>2857	>MMa20191	YGNACWCIDLPDKVPIR
>2858	>comp86_c0_seq1_2	YCSENPLDCNEHCLK
>2859	>MMa34629	LPDKVPIR
>2860	>comp105006_c0_seq1_4	LATVISPR
>2861	>comp11050_c0_seq1_5	NIPQLVSPR
>2862	>comp20_c0_seq1_3	YAVPEGTLR
>2863	>comp105006_c0_seq1_4	LATVISPR
>2864	>comp7_c0_seq5_6	IGYCNIQGK
>2865	>MMa02192	VSCLWGNEGCNK
>2866	>comp105006_c0_seq1_4	LATVISPR
>2867	>comp164574_c0_seq1_2	KSEYISIR
>2868	>MMa17865	KSELWNYNTNK
>2869	>MMa17865	LACFCQGAR
>2870	>MMa17865	VWCVINNESCNSECK
>2871	>comp33184_c0_seq4_1	IMGDIINK
